# The Important Role of Alloy–Oxide Interfaces in Controlling Methanol Formation in CO_2_ Hydrogenation

**DOI:** 10.1021/acscatal.5c06703

**Published:** 2026-01-13

**Authors:** Naomi Lawes, Igor Kowalec, Sofia Mediavilla-Madrigal, Kieran J. Aggett, Louise R. Smith, Malcolm Dearg, Thomas J. A. Slater, Eimear McCarthy, Herzain I. Rivera-Arrieta, Matthias Scheffler, David J. Morgan, David J. Willock, Andrew M. Beale, Andrew J. Logsdail, Nicholas F. Dummer, Michael Bowker, C. Richard A. Catlow, Stuart H. Taylor, Graham J. Hutchings

**Affiliations:** † Max Planck-Cardiff Centre on the Fundamentals of Heterogeneous Catalysis FUNCAT, Translational Research Hub, 2112 Cardiff University, Maindy Road, Cardiff CF24 4HQ, U.K.; ‡ UK Catalysis Hub, Research Complex at Harwell, Rutherford Appleton Laboratory, Didcot OX11 0FA, U.K.; § School of Physics, Engineering and Technology, University of York, York YO10 5DD, U.K.; ∥ The NOMAD Laboratory at the Fritz-Haber-Institut of the Max-Planck-Gesellschaft and IRIS-Adlershof of the Humboldt-Universität zu Berlin, 14195 Berlin, Germany; ⊥ HarwellXPS—EPSRC National Facility for Photoelectron Spectroscopy, Research Complex at Harwell (RCaH), Didcot OX11 0FA, U.K.; # Chemistry Department, 4919 University College London, 20 Gordon Street, London WC1H 0AJ, U.K.

**Keywords:** CO_2_ hydrogenation, Pd Zn catalysts, alloy formation, PdZn/ZnO interface, methanol

## Abstract

A series of PdZn/TiO_2_ catalysts prepared by chemical vapor impregnation (CVI) were tested for CO_2_ hydrogenation at 20 bar pressure and at temperatures of 230–270 °C. Changing the Pd and Zn molar ratio (Zn:Pd = 0–20) in a PdZn/TiO_2_ catalyst has a dramatic effect on selectivity for the CO_2_ hydrogenation reaction. Pd alone shows three main products: methanol, CO, and methane. Addition of small quantities of Zn results in the formation of a PdZn alloy, preventing methanation. At equimolar ratios of Pd and Zn, a 1:1 β-PdZn alloy is formed and a reverse water gas shift catalyst is produced. Adding Zn in excess relative to the Pd loading results in the formation of ZnO on the TiO_2_ surface in addition to the PdZn alloy, dramatically increasing methanol selectivity from 5% at Zn:Pd = 1 to 55% for Zn:Pd = 2. Through a combination of theory and experiment, the active site for methanol synthesis is concluded to be the interface between PdZn nanoparticles and the ZnO overlayer on the TiO_2_, where interfacial formate can react with hydrogen dissociated by the metal nanoparticle.

## Graphical abstract

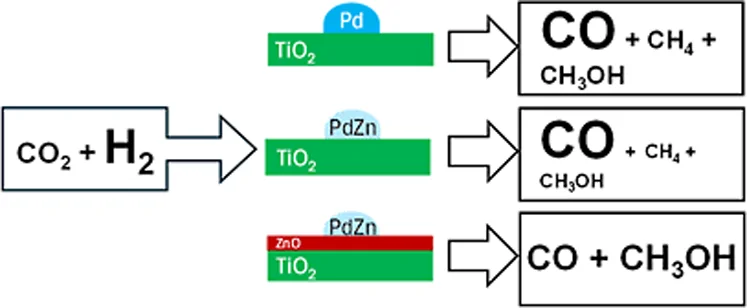

## Introduction

The rise in anthropogenic CO_2_ and the impact of the greenhouse effect on the climate demand action to prevent long-term damage to global ecosystems and societies. Global CO_2_ emissions from the energy sector and other industrial processes reached a record annual level of 37.8 gigatonnes in 2024, as estimated in the International Energy Agency’s analysis report.^1^ This carbon-intensive growth is a stress for sustainable investments and clean energy technologies. However, carbon capture and utilization will be important to tackle the continuing rise of CO_2_ emissions. Valuable utilization can be provided through methanol production, opening a nonfossil fuel route to value-added chemicals, fuels, and an energy storage medium.^2,3^ When combined with hydrogen supplied from sustainable resources, such as water splitting from electrolysis or photocatalysis, the hydrogenation of CO_2_ to methanol offers a portable energy solution that can help to reduce fossil fuel and natural gas usage and facilitate the transition to net zero.

Commercially, methanol is commonly produced from syngas, a mixture of CO_2_, CO, and H_2_, using a Cu/ZnO/Al_2_O_3_ catalyst. Cu/ZnO/Al_2_O_3_ produces favorable methanol selectivity because of a synergy between Cu and ZnO.^4–6^ Al_2_O_3_ is also thought to accelerate CO activation.^7^ More recently, the Cu/ZnO/Al_2_O_3_ catalyst has been applied to the direct hydrogenation of carbon dioxide to methanol, to reduce dependence on syngas, which is typically derived from fossil resources, in the hope that Cu/ZnO/Al_2_O_3_ could be utilized for control of CO_2_ emissions.^8^ However, various concerns have been reported with this catalytic system, for CO_2_ hydrogenation, regarding Cu sintering and reduced stability when in the presence of water, a byproduct of methanol synthesis from CO_2_.^9–12^ Optimizing the activity, methanol selectivity, water tolerance, and stability are all crucial components of a successful catalyst for the hydrogenation of CO_2_ to methanol. Selective CO_2_ hydrogenation to methanol is constrained by thermodynamic and kinetic limitations, as direct CO_2_ hydrogenation (eq 1) is an exothermic process, with methanol synthesis favored at low temperatures and high pressures. Additional challenges include competing reactions, such as methanation (eq 2)^13^ and the reverse water gas shift reaction (RWGS, eq 3), which produces CO. Distinct from methanol synthesis, RWGS is endothermic, so it is favored at high temperatures. Despite the desire to produce catalysts with high methanol selectivity, CO is industrially a less disadvantageous byproduct compared to methane and water, as CO can also be converted into methanol (eq 4) and so any unreacted CO could be recycled back into the catalyst bed.^14^

1 $${CO}_{2}+3H_{2}⇌{CH}_{3}OH+H_{2}O;,\Delta H_{298K}=-49.5\;kJ\;{mol}^{-1}$$

2 $${CO}_{2}+4H_{2}⇌{CH}_{4}+{2H}_{2}O;,\Delta H_{298K}=-252.9\;kJ\;{mol}^{-1}$$

3 $${CO}_{2}+H_{2}⇌CO+H_{2}O;,\Delta H_{298K}=41.3\;kJ\;{mol}^{-1}$$

4 $$CO+2H_{2}⇌{CH}_{3}OH;,\Delta H_{298K}=-90.6\;kJ\;{mol}^{-1}$$

Pd catalysts are well-known for hydrogenation reactions but, under CO_2_ hydrogenation reaction conditions, CO formation is favorable via the RWGS reaction^15^ (eq 3) and methane and methanol can also be produced. Previous studies have shown an increase in methanol selectivity, attributed to the formation of the 1:1 β-PdZn alloy, when Pd/ZnO is reduced above 300 °C.^16–19^ Peterson et al. combined X-ray and neutron powder diffraction data analysis to precisely describe the nature of the tetragonal β-PdZn and showed that it is the most prevalent alloy phase, despite the complicated PdZn phase diagram.^20^ Friedrich et al., Heggen et al., and Miyazaki et al. studied PdZn nanoparticle catalysts and identified the tetragonal structure also, with (101) surfaces as the most abundant facets, using various microscopy methods.^21–23^ Similarly, tetragonal PdZn was the most abundant phase in the nanoalloy particles synthesized by Nowicka et al., with the (110) facets being especially prominent.^24^ The PdZn alloy shows features of a perfectly ordered (o-) material with a pattern of alternating rows of Pd and Zn on the (110) surfaces.^21–24^ Moreover, the 1:1 composition of the alloy appears to be obtained regardless of the starting Pd and Zn precursor ratio, which is attributed to the high exothermic heat of mixing of Pd and Zn metals in the 1:1 alloy.^18^ Following the naming convention of Crawley et al., the specific highly symmetric ordering for the 1:1 PdZn alloy warrants recognition as the o-Pd_0.5_Zn_0.5_ phase, but hereafter, we refer to PdZn for simplicity.^25^

Temperature-programmed desorption (TPD) showed that, under the right conditions, CO_2_ can adsorb strongly on the ZnO powder alone;^26^ however, more recently, van Bokhoven et al.^27^ demonstrated that methanol selectivity is improved when both ZnO and the PdZn alloy are present, when comparing PdZn/SiO_2_ with PdZn/ZnO/SiO_2_. The 1:1 β-PdZn alloy has usually been assigned as the active phase.^10,11^ However, through in situ and operando studies, Bokhoven et al.^27^ demonstrated a multifunctional mechanism, whereby CO_2_ was activated by ZnO and the PdZn alloy functioned to dissociate hydrogen. Formate formation was enabled at the interface of PdZn and ZnO, which, in turn, resulted in an increase in methanol production. Quilis et al. synthesized PdZn–ZnO catalysts on anatase TiO_2_ with Zn:Pd molar ratios at 2.5, 5, and 7.5, whereby increased methanol yield was attributed to improved PdZn–ZnO interfaces.^28^ Previous work also demonstrated changes in methanol selectivity when examined at 230 °C for CO_2_ hydrogenation with reduced Zn quantities in a PdZn/TiO_2_ catalyst.^15^ The Zn:Pd ratios investigated included those falling below the threshold for complete 1:1 PdZn alloy formation.

The current work aims to further investigate the relationship between Pd and ZnO using a series of catalysts based on Pd/TiO_2_ with increasing quantities of added Zn. By supporting Pd and Zn on TiO_2_, the Zn:Pd ratio can be varied to understand the influence of excess Zn on catalyst performance and to effectively afford a higher surface area for the ZnO. We then link catalyst activity to detailed analysis, by a range of approaches including in situ methods, and combine this with theoretical calculations of the CO_2_ adsorption structure and energies on the various surfaces involved. This approach leads to a detailed understanding of the catalytic reactivity. We further link our observations to the behavior of CO_2_ adsorption on ZnO surfaces and CO_2_, CO, and hydrogen on tetragonal β-PdZn(101) and β-PdZn(110) alloy surfaces in the context of CO_2_ hydrogenation to methanol.^29–31^

## Materials and Methods

### Materials

The following chemicals and reagents were purchased from Sigma-Aldrich: palladium acetylacetonate (Pd­(acac)_2_, 99%), zinc acetylacetonate (Zn­(acac)_2_, 99%), ZnO (99.9%, nanopowder, <100 nm particle size), and TiO_2_ (P25, Evonik). The chemicals were used as provided without further purification.

### Chemical Vapor Impregnation (CVI) Synthesis

Catalysts were prepared using CVI with a fixed Pd loading of 5 wt %. The procedure for preparing 5 wt % Pd_*x*_Zn_*y*_/TiO_2_ commenced with the desired amounts of Pd­(acac)_2_, Zn­(acac)_2_, and TiO_2_ being physically mixed in a glass vial for 1 min. The mixture was transferred to a Schlenk flask and sealed. The tube was connected to a Schlenk line and evacuated (∼10^–3^ mbar). The tube was then lowered into an oil bath preheated to 80 °C, and the temperature was increased to 133 °C and maintained for 1 h. The material was then recovered and annealed in static air (500 °C, 5 °C min^–1^, 16 h). Varying the Zn precursor quantities allowed the preparation of the catalyst with various Zn loadings compared with 5 wt % Pd. It is noted that the descriptor Pd_*x*_Zn_*y*_/TiO_2_ hereafter refers to the total Zn:Pd molar ratio of *y*/*x* on TiO_2_ and not the β-PdZn alloy that is formed following reduction.

### CO_2_ Hydrogenation Catalyst Testing

Catalytic testing for CO_2_ hydrogenation used a 16-bed high throughput catalytic reactor unit operating at a pressure of 20 bar and at temperatures from 230 to 270 °C. Prior to testing, the catalysts were reduced in situ in a flow of 5% H_2_/N_2_ gas (400 °C, 1 h, and 5 °C/min) and subsequently cooled to 125 °C under N_2_. The gas composition was switched to the reactant gas (20% CO_2_, 60% H_2_, 5% Ar, 15% N_2_) with a flow rate of 30 mL min^–1^ at atmospheric pressure and then pressurized. Further details of the reactor and its operation are given in the Supporting Information (Section S1).

### Catalyst Characterization

Powder X-ray diffraction (XRD) patterns were measured on two PANalytical X’pert Pro powder diffractometers operating at 40 kV, 40 mA using Cu Kα radiation (λ = 1.54 × 10^–10^ m) with a Ge(111) single crystal monochromator, one for ambient measurements and one for in situ XRD.

X-ray photoelectron spectra (XPS) were recorded on a Kratos Axis Ultra-DLD fitted with a monochromatic Al Kα (75–150 W) source and an analyzer using a pass energy of 40 eV. XPS data were analyzed using Casa XPS software.

Brunauer–Emmett–Teller (BET) surface areas were measured by using nitrogen adsorption at −196 °C on a Quantachrome Nova 2200e instrument. Prior to N_2_ adsorption, samples were degassed in situ (150 °C, 3 h), and the results are shown in Table S1.

An Agilent 7900 inductively coupled plasma mass spectroscopy (ICP–MS), with an Agilent integrated autosampler (I-AS), was used to quantify total metal loading. The catalysts were digested (50 mg of catalyst, 5 mL of aqua regia) using a Milestone ETHOS LEAN, EASY, and UP microwave digestion system. Pd loadings were found to be between 5.0 and 5.8 wt % compared to the target loading of 5.0 wt %, and observed Zn loading values were generally within 20% of the target used in synthesis. These variations meant that the observed Zn:Pd molar ratio is generally within 6% of the values quoted based on preparation target values, although there are outliers at Zn:Pd = 1.5 (1.23 observed) and at large excesses of Zn; e.g., Zn:Pd = 20 has a molar ratio of 15.9, as observed from the measured data (Table S1).

Transmission electron microscopy (TEM) was performed on a JEOL JEM-2100 operating at 200 kV. Energy-dispersive X-ray analysis (EDX) was done using an Oxford Instruments X-MaxN 80 detector, and the data were analyzed using the Aztec software. Samples were deposited on 300 mesh copper grids coated with a holey carbon film. Scanning transmission electron microscopy (STEM) utilized a JEOL ARM200F microscope at the electron Physical Sciences Imaging Centre (ePSIC) at Diamond Light Source. EDX acquisition was performed using a JEOL Centurio dual-detector. Samples were subsequently also imaged using a probe-corrected 200 kV Thermo-Scientific Spectra 200 Scanning Transmission Electron Microscope. An acceleration voltage of 200 kV, a convergence semiangle of 23 mrad, a high-angle annular dark field (HAADF) inner angle of 80 mrad, and an outer angle of approximately 200 mrad were used for data collection. EDX spectrum images were acquired on a Super-X detector using all four detectors.

In situ X-ray Absorption Spectroscopy (XAS) studies were conducted at the Swiss-Norwegian BM31 beamline at the European Synchrotron Radiation Facility.^32,33^

Further details for each characterization method are given in Supporting Information (Section S2).

### Computational Methods

The Fritz Haber Institute ab initio molecular simulations (FHI-aims) software package was used for full potential all-electron density functional theory (DFT) calculations, with the Pythonic Atomic Simulation Environment (ASE) used for the management of calculation geometries.^33,34^ We used the FHI-aims’ Force Correction functionality to speed up the convergence of the DFT simulations without loss of accuracy. This implementation eliminates the fluctuations of the Hellmann–Feynman forces for loosely converged electron densities.^35^ For kinetic studies, a machine learning nudged elastic band (MLNEB) method was used to identify saddle points and minimum energy paths (MEPs).^36,37^ A spring constant of 0.05 eV Å^–1^ was deemed sufficient for well-converged MEPs in our previous work and has been used throughout, the convergence criterion of forces on all unconstrained atoms of below 0.05 eV Å^–1^, with energy uncertainty below 0.03 eV.^31^ The activation energy of a reaction step, *E*_act_(species), is defined as the difference between the total energy of the transition state at the surface and the total energy of the initial geometry of the species at the surface. *E*_act_(species)_d_ and *E*_act_(species)_h_ denote dissociation and hydrogenation processes, respectively. Further details of these calculations, including surface site definitions, can be found in the Supporting Information (Section S3, Figures S1–S3).

## Results and Discussion

### Catalytic Performance

Figure 1 shows the relationship between the CO_2_ conversion and selectivity for Pd/TiO_2_ and Pd_*x*_Zn_*y*_/TiO_2_ catalysts with varying Zn:Pd molar ratios (Zn:Pd = 0.1–20). The series of catalysts were prepared by CVI and calcined in static air at 500 °C for 16 h to ensure the complete decomposition of the acetylacetonate precursors. The samples were prereduced in situ (400 °C, 1 h, 5% H_2_/Ar) prior to catalytic testing. The data shown are an average of four data points taken separately every 8 h at a reaction temperature of 230 °C. All catalysts show activity for CO_2_ hydrogenation with varying selectivity toward the main products: methanol, CO, and CH_4_. Only trace amounts of DME (<0.2%) and negligible levels of other products were observed. A carbon balance over 99% was achieved for all catalysts, and the stability was maintained for the duration of the testing (ca. 100 h). Figure S7 shows representative results over the testing period for high and low Pd:Zn ratio catalysts.

**Figure 1:**
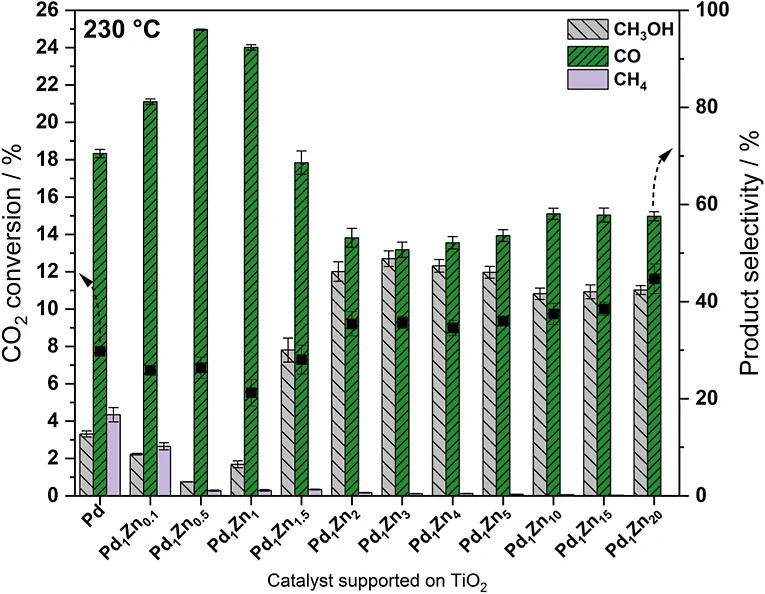
CO_2_ conversion and product selectivity at 230 °C for Pd/TiO_2_ and Pd_*x*_Zn_*y*_/TiO_2_ catalysts with varying Zn:Pd molar ratios (see SI for 250 and 270 °C). ■ represents CO_2_ conversion (right axis); histogram bars represent selectivity of products indicated in key (left axis).

The P25 support for these reactions was chosen as it is known that Pd/TiO_2_ is a RWGS catalyst, which will reduce CO_2_ to methane when larger Pd nanoparticles are present. Accordingly, very low methanol production would be expected from the monometallic catalyst prior to the addition of Zn. Figure 1 confirms that the Pd/TiO_2_ used in this study has CO as the major product, as supported by a recent report by Quilis Romero et al.,^38^ but produces significant amounts of methane (17%) and some methanol (13%), at 230 °C. At the lowest Zn:Pd ratio of 0.1, (Pd_1_Zn_0.1_), there is already a dramatic effect on methane selectivity, dropping to 10% at a comparable CO_2_ conversion (Figure 1 and Table S2) compared to Pd/TiO_2_. As the Zn:Pd ratio is increased from 0.1 to 0.5, methane selectivity decreases to 1% and methanol selectivity also decreases, from 13% for Pd/TiO_2_ to 3% for Pd_1_Zn_0.5_/TiO_2_. At Zn:Pd = 0.5, the RWGS performance peaks, with a selectivity for CO of 96%. On further increasing the ratio, i.e., Zn:Pd ≥ 1, methane productivity continues to decline, but methanol selectivity sharply increases, and CO selectivity reduces. For 2 ≤ Zn:Pd ≤ 5, the CO selectivity remains reasonably constant (within experimental uncertainty) and is matched by the methanol selectivity that accounts for up to 50% of the products (Pd_1_Zn_3_/TiO_2_ catalyst). For the very highest Zn:Pd ratios studied here, 5 ≤ Zn:Pd ≤ 20, the major product is again CO, but methanol selectivity remains above 40%.

Data for CO_2_ reduction using the same range of catalyst compositions at 250 and 270 °C are given in the Supporting Information (Figures S4 and S5) and show similar trends in reactivity. Throughout the temperature range, when considering Zn:Pd ratios above 1.5, methane selectivity remains low (≤1%). The main differences seen with increasing temperature are (i) an increase in conversion and (ii) an increase in methane production for the Pd-only catalysts, reaching a methane selectivity of 34% at 270 °C, at the expense of methanol and CO production.

Previously, methane was thought to be a product of methanol decomposition on exposed titania surface sites^13^ or through methanation on Pd metal.^39,40^ Here, we note that both methanol and methane selectivity decrease upon doping Pd/TiO_2_ with 0 ≤ Zn:Pd ≤ 0.5, concurrent with increased CO selectivity, while at higher ratios, increased methanol production coincides with a sharp reduction in CO selectivity. It is therefore proposed that methane and CO are produced through the dissociation of CO_2_ on the surface of the metallic component of the catalyst, as discussed in detail below, with a negligible contribution from methanol decomposition via steam reforming. The resulting adsorbed CO species is associated with Pd atoms and can either desorb or dissociate further. The latter option leads to hydrogenation of adsorbed carbon species to give methane production. The proposed reactions are given in eqs 5–8, where (*g*) and (*a*) refer to the gas phase and adsorbed species, respectively.

5 $${CO}_{2}\left(g\right)+H_{2}\left(g\right)\to CO\left(a\right)+H_{2}O\left(a\right)$$

6 CO(a)→CO(g)

7 CO(a)→C(a)+O(a)

8 $$C\left(a\right)+O\left(a\right)+3H_{2}\left(g\right)\to {CH}_{4}\left(g\right)+H_{2}O\left(g\right)$$

Figure 1 also shows that upon the initial increase of Zn (from Zn:Pd = 0 to Zn:Pd = 1.0), there is a small decrease in CO_2_ conversion at 230 °C, from 8 ± 1% (Zn:Pd = 0) to 6 ± 1% (Zn:Pd = 0) despite significant variation in selectivity. For Zn:Pd > 1.5, however, CO_2_ conversion increases to around 10%. The increase in CO_2_ conversion may be attributed to the presence of excess ZnO.^15,28^ The performance of these TiO_2_-supported catalysts at high Zn:Pd ratios, i.e., with Zn excess, is only slightly inferior to that of a range of Pd/ZnO catalysts reported previously.^18^

It is important to note that the catalyst surface area changes at higher loadings of Zn (Figure S11 and Table S1). The area for Pd/TiO_2_ (52 m^2^ g^–1^) is dictated by the TiO_2_ surface area, but as the Zn loading increases, especially above a Pd:Zn ratio of 1, the surface area begins to decrease, eventually reaching 30 m^2^ g^–1^ at the highest Zn loading considered (Pd_1_Zn_20_). This is due to the decreasing amount of titania in the catalysts as the presence of Zn (such as PdZn and ZnO) increases. For 5% Pd/TiO_2_, there is 95 wt % TiO_2_, but for Zn:Pd = 20, there is only 34 wt %.

The dependence of methanol productivity of Pd_*x*_Zn_*y*_/TiO_2_ on the variation of the Zn:Pd ratio is shown in Figure S6 for temperatures of 230 °C, 250°C, and 270 °C. The dependence of productivity follows the trend expected from the methanol selectivity in Figures 1, S4, and S5. At low Zn levels, there is a decrease in productivity compared to the Pd/TiO_2_ catalyst, but then, when the Zn:Pd ratio exceeds the equimolar ratio, a sharp increase in productivity is observed, with each temperature showing a plateau within the experimental error for Zn:Pd ≥ 2. The methanol productivity seen in this region exceeds 1500 mmol h^–1^ kg_cat_^–1^ for the experiments conducted at 250 °C.

### XRD Analysis

X-ray diffraction (XRD) was used to gain information about alloy formation in the reduced catalysts (5% H_2_/Ar, 400 °C, 1 h). XRD patterns as a function of catalyst composition are given in Figure 2a in the diffraction angle range 39–50° with the broader scan pattern included in Figure S8 (Supporting Information). The β-PdZn 1:1 alloy has previously been shown to form upon reduction of a Pd/ZnO catalyst at temperatures above 250 °C.^16,27,41,42^ The characteristic (111) and (200) β-PdZn alloy reflections are located at 41.2° and 44.1°. However, in these samples at low Zn:Pd ratios, these reflections are hard to distinguish due to the overlap with the (111) and (210) planes of rutile TiO_2_. The prominence of the (111) reflection of Pd metal and the reflections from the TiO_2_ phases clearly decrease with increasing Zn loading, coinciding with the appearance of β-PdZn alloy reflections at 41.2° and 44.1°, which become more prominent. Additionally, as the Zn:Pd ratio is increased above 3, the development of the (100), (002), and (101) reflections for ZnO can be observed at 31.8°, 34.4°, and 36.3°, respectively. It appears that Zn can be incorporated to form a β-PdZn alloy, and once Zn loadings are above that required for the stoichiometric formation of the 1:1 β-PdZn (Zn:Pd > 1), ZnO is likely to be formed.^15^ Below Zn:Pd = 3, the ZnO reflections are not visible, which may be because the ZnO layer is too thin to be observed by XRD.

**Figure 2:**
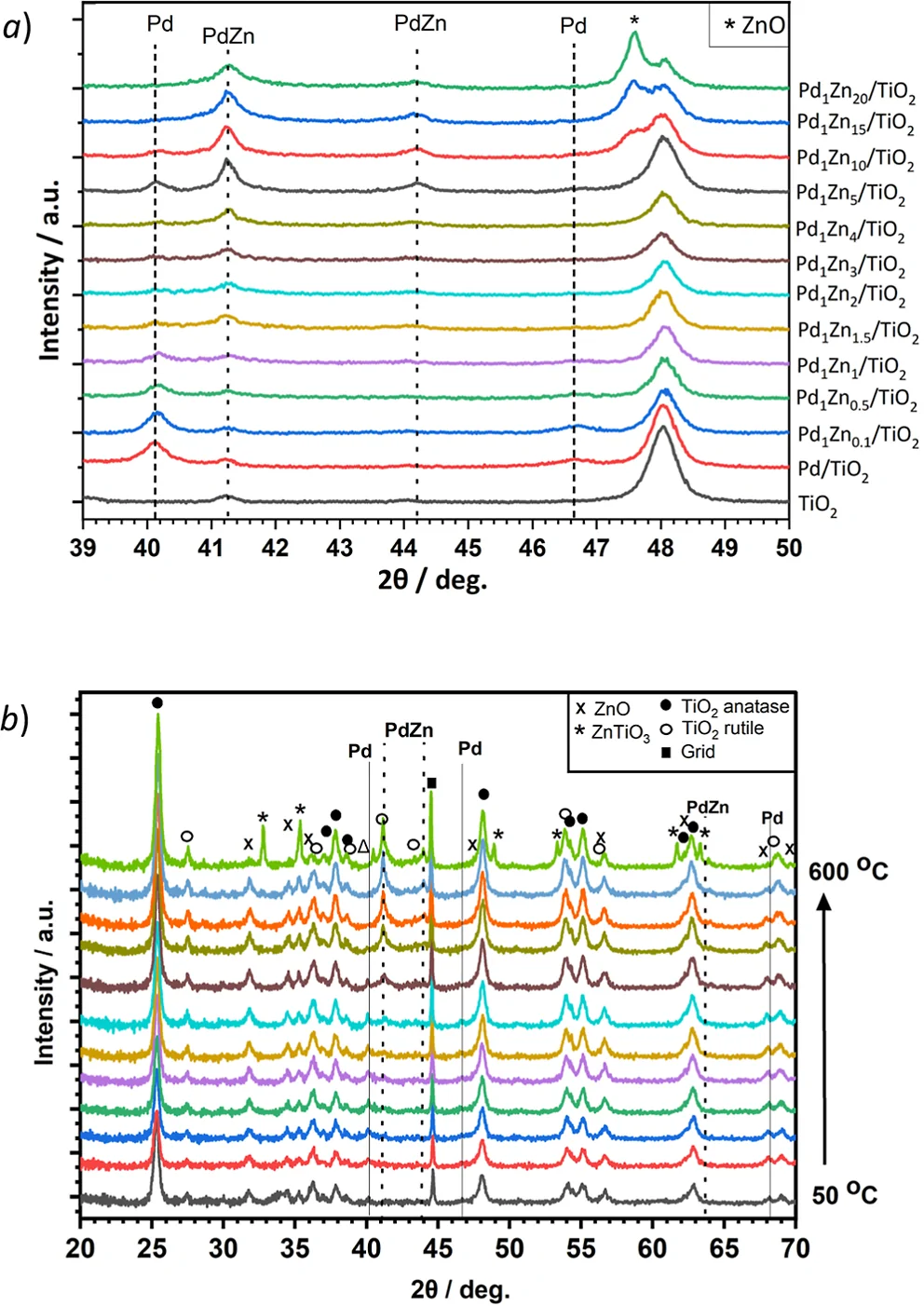
(a) Powder X-ray diffraction patterns at 2θ (deg.) between 39° and 50° for TiO_2_, Pd/TiO_2_, and Pd_*x*_Zn_*y*_/TiO_2_ catalysts with varying Zn:Pd ratios after calcination at 500 °C for 16 h, followed by H_2_ reduction at 400 °C. (b) In situ powder X-ray diffraction patterns of Pd_1_Zn_5_/TiO_2_ under reducing conditions (5% H_2_/Ar, 50–600 °C, 50 °C steps). Reflections observed in the in situ XRD experiments at 35.5° and 44.7° were a result of the grid/sample holder; see Figure S9.

To follow alloy formation as a function of temperature, in situ XRD measurements were performed for the Pd_1_Zn_5_/TiO_2_ catalyst under reducing conditions (5% H_2_/Ar) over a temperature range from 50 to 600 °C in 50 °C steps (Figure 2b). Some anatase and rutile reflections overlap with those of PdO, PdZn, and ZnO (Table S3). The PdO reflection at 33.6° is lost almost instantly as the temperature increases from 50 to 100 °C. The reduction of PdO to Pd at low temperatures probably facilitates hydrogen spillover to the ZnO phase, facilitating reduction and the formation of the PdZn alloy.^41,43,44^ The reflections observed at 41.2° and 44.2° are characteristic of the 1:1 β-PdZn alloy (111) and (200) planes, respectively, which become prominent from 350 °C, though with some Pd still present. As heating continues, Pd reflections can no longer be seen at 450 °C. ZnO is already formed following calcination, as can be seen from the reflections at 34.4°, 36.3°, and 47.5° at the lowest temperature. These peaks persist as the sample is heated, but at 600 °C, a noticeable phase change occurs, where the ZnO reflections diminish and new reflections are observed at 33°, 35°, 49°, 53°, and 62°, most likely corresponding to the formation of a ZnTiO_3_ phase, as observed previously by Bahruji et al.^45^ Indeed, from the loss of ZnO features, most of the ZnO appears to be converted to zinc titanate.

### XPS Analysis

To further study the extent of PdZn alloying and surface properties, Pd_1_Zn_5_/TiO_2_ and Pd_1_Zn_15_/TiO_2_ catalysts were characterized by XPS analysis comparing the calcined, reduced, and postreaction samples (Figure S10). Both samples show a peak at 336.3 eV following calcination (Figure S10a,d), corresponding to Pd^2+^ present as PdO. After reduction at 400 °C (Figure S10 b and e), Pd_1_Zn_5_/TiO_2_ and Pd_1_Zn_15_/TiO_2_ show a shift to lower binding energy corresponding to Pd metal at 334.4 eV and Pd alloying with Zn at 335.4 eV.^19,46^ Samples were retrieved after catalytic testing (Figure S10c,f), corresponding to Pd_1_Zn_5_/TiO_2_ and Pd_1_Zn_15_/TiO_2_. Interestingly, the ratios between Pd metal and Pd alloy after reduction and postreaction are similar for Pd_1_Zn_5_/TiO_2_ but a slightly higher signal for the Pd alloy is observed after reaction for Pd_1_Zn_15_/TiO_2_, suggesting that the PdZn alloy may be completed under CO_2_ hydrogenation testing conditions. More details from the XPS analysis are given in the Supporting Information (Section S5).

### Nanoparticle Structure and Morphology

The diameter and shape of nanoparticles in Pd/TiO_2_ and Pd_*x*_Zn_*y*_/TiO_2_ series were evaluated, following reduction of the samples at 400 °C, using a combination of TEM and HAADF-STEM with EDX mapping. HAADF-STEM images are given in Figure 3, while representative micrographs, particle diameter distributions, and mean particle diameters are shown in the Supporting Information, Section S6 and Table S4. For the Pd_*x*_Zn_*y*_/TiO_2_ catalysts with the higher Zn loadings (ZnPd > 2), formation of ZnO clusters makes analysis of particle diameters very challenging without elemental mapping.

**Figure 3:**
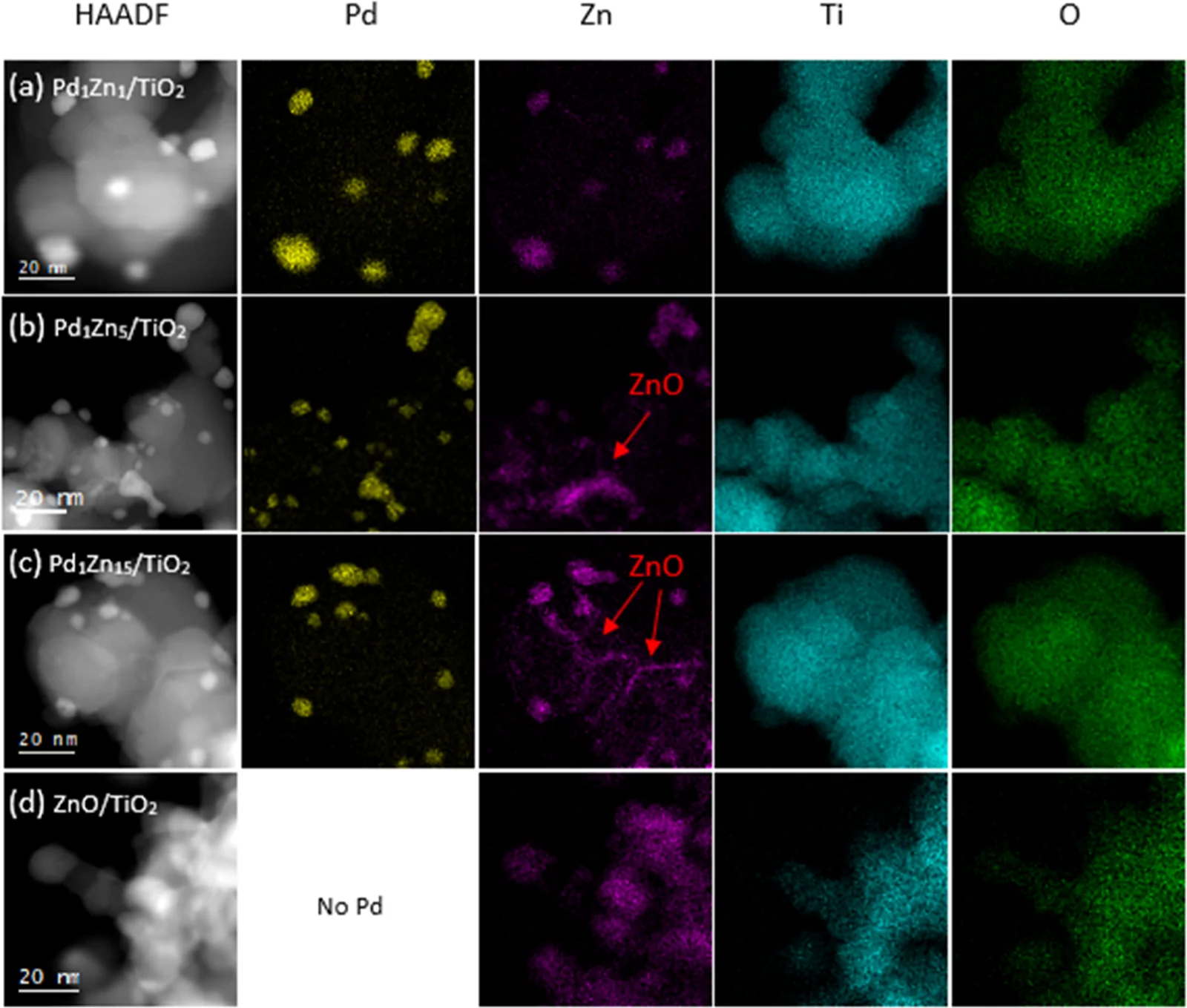
HAADF-STEM imaging and EDX elemental mapping, with the scale bar presenting 20 nm, demonstrating ZnO coverage and/or PdZn alloy formation after reduction at 400 °C for (a) Pd_1_Zn_1_/TiO_2_, (b) Pd_1_Zn_5_/TiO_2_, (c) Pd_1_Zn_15_/TiO_2_, and (d) ZnO/TiO_2_. EDX maps are produced from a sum of all the counts in the Kα peak for Zn, O, and Ti and the Lα_1_ peak for Pd.

Pd/TiO_2_ has a mean particle diameter of 5.7 nm, while Pd_*x*_Zn_*y*_/TiO_2_ has a range of particle diameters between 4.3 and 8.3 nm (Table S4), and from TEM and HAADF-STEM images, the nanoparticles generally appear to be approximately part-spherical in shape (Figure 3). Surprisingly, the mean particle sizes show no clear trend with Zn loading, the average of the mean particle diameters for all catalyst compositions being 6.6 nm with a standard deviation of ±0.9 nm. CVI has previously been shown to result in well-dispersed metal particles on a range of supports.^19,47,48^ Bahruji et al. reported PdZn nanoparticles supported on ZnO, TiO_2,_, and Al_2_O_3_ with particle diameters between 3 and 6 nm.^19^ Therefore, the systematic change in catalytic selectivity observed as Zn loading is increased is unlikely to be linked to a particle diameter effect. This indicates that excess ZnO is likely to lead to more methanol production rather than any effect on the metal particle size.^15,27,28^

EDX elemental maps of Pd_1_Zn_1_/TiO_2_, Pd_1_Zn_5_/TiO_2_, and Pd_1_Zn_15_/TiO_2_ post reduction (5% H_2_/Ar, 400 °C, 1 h) are also shown in Figure 3 alongside images for a ZnO/TiO_2_ material prepared using our standard synthesis method but without the inclusion of Pd. For the Pd_1_Zn_1_/TiO_2_ sample (Figure 3a), most of the Zn appears associated with Pd nanoparticles, likely in the form of the β-PdZn alloy in an equimolar ratio.^15,18,49^ For Zn:Pd > 1 (Figure 3b and c), the EDX maps again show Zn associated with Pd but also more widely distributed, likely as ZnO present at the surface of the sample, as suggested by XRD analysis. ZnO is not as evident at Zn:Pd ≤ 1, which is presumed to be an absence of bulk formation, but the formation of a thin layer on Pd and/or the TiO_2_ support cannot be ruled out.^15^ These results suggest that the change in catalytic activity observed for Zn:Pd ratios ≥2 (Figures 1 and S4–S6) could be correlated with significant levels of ZnO at these Zn:Pd ratios. The ZnO surface layers appear essential for producing methanol, with methanol selectivity increasing for Zn:Pd > 1 and a plateau in productivity when Zn is in excess (Zn:Pd ≥ 2), as discussed earlier.

To further understand the growth of ZnO on the surface of these catalysts, CVI was used to add Zn to TiO_2_ at a level matching the wt % of Zn in a Pd_1_Zn_15_/TiO_2_ catalyst (∼40 wt % Zn). HAADF imaging and EDX mapping (Figure 3d) indicate that ZnO covers the majority of TiO_2_. In Section S8, we show that the Zn loading in Pd_1_Zn_18_/TiO_2_ should be sufficient to form a PdZn alloy and evenly distribute ZnO at a monolayer coverage. So, the mapping in Figure 3d is consistent with an evenly spread thin film of ZnO on the TiO_2_ support. However, when we compare the EDX map for Pd_1_Zn_15_/TiO_2_, the same level of Zn is clearly colocating with Pd. It is likely that the favorable heat of mixing for Zn and Pd (∼0.6 eV/atom) identified previously^18^ is a driving force for the β-PdZn alloy formation. It is also apparent that the metals are mobile on the surface and so can move to form the alloy particles with the ZnO component of the material closely associated with these nanoparticles.

### X-ray Absorption Spectroscopy

Figure 4a shows Pd K-edge XANES spectra for Pd_1_Zn_1_/TiO_2_ and Pd_1_Zn_20_/TiO_2_ collected after a reduction treatment in 5% H_2_/Ar compared with Pd foil and PdO standards. The red shift of the edge energy compared to the Pd foil reference and the emergence of a feature at ca. 24,400 eV have previously been reported to originate from the formation of a PdZn alloy.^16,50,51^ In addition, both samples show features indicating a metallic state for Pd including features at ca. 24,390 eV and 24,430 eV characteristic of nonalloyed metallic Pd nanoparticles. These features are lower in amplitude for Pd_1_Zn_20_/TiO_2_ than for Pd_1_Zn_1_/TiO_2_. Thus, the Pd_1_Zn_1_/TiO_2_ spectrum indicates a mix of metallic Pd and some PdZn alloy phases, with the metallic Pd XANES features still quite prominent, whereas the XANES of Pd_1_Zn_20_/TiO_2_ is dominated by the alloy phase. Figure 4b shows the Fourier transform of the Pd XANES spectra, which also highlights these differences. The Pd_1_Zn_1_/TiO_2_ plot contains a very broad peak at around 2.4 Å, corresponding to the main peak of the Pd foil reference. This is accompanied by a shoulder at a lower radial distance (≈1.9 Å), which has previously been assigned to PdZn in the literature.^16,27,50,52^ For Pd_1_Zn_20_/TiO_2_, the main peak is shifted to 2.2 Å with a skew toward shorter distances consistent with an increased contribution from the 1.9 Å shoulder (PdZn) and a decrease in the contribution from pure metallic Pd. This is consistent with higher Zn content leading to a greater extent of alloying.^27^

**Figure 4:**
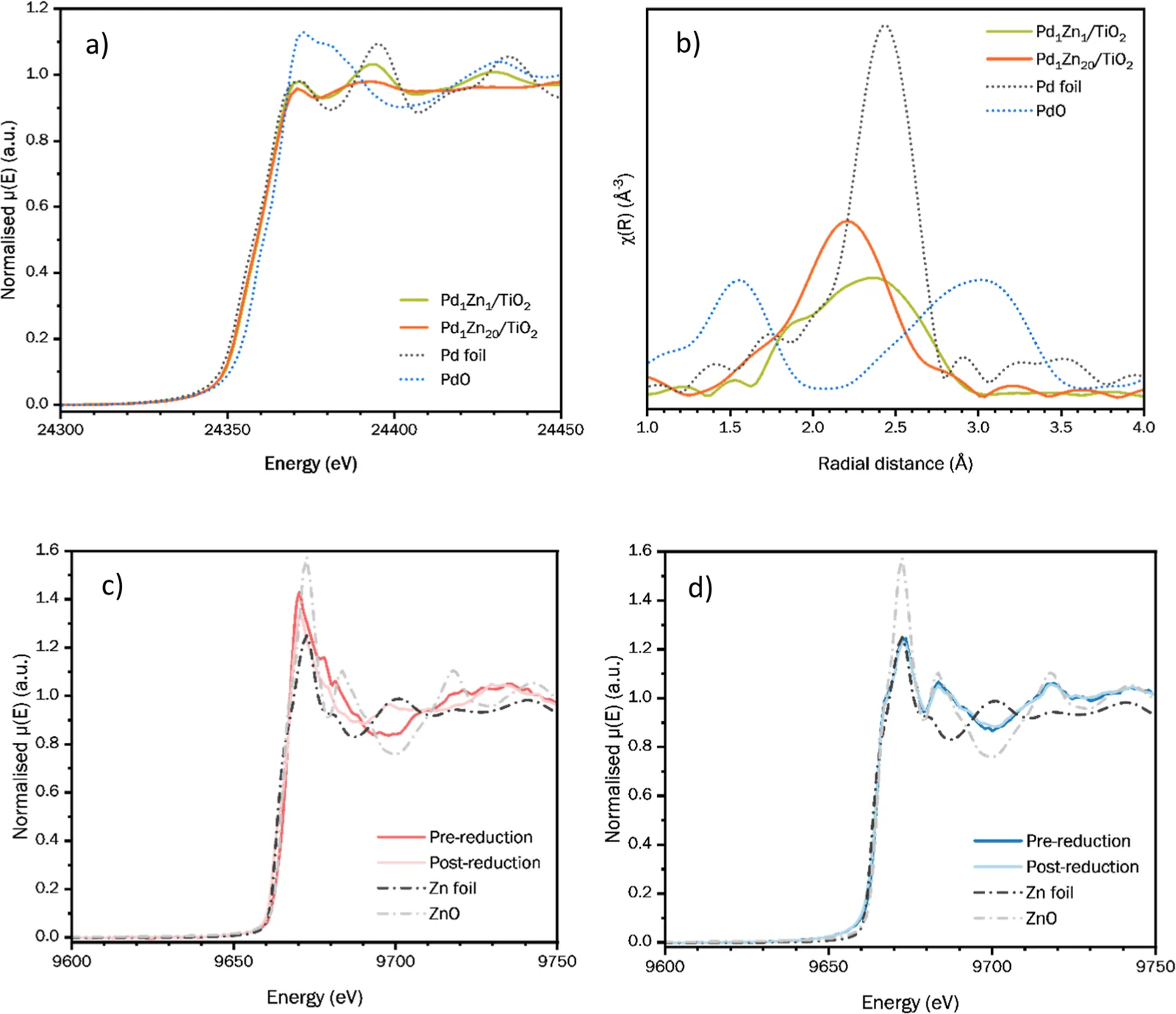
(a) Pd K-edge XANES and (b) the corresponding Fourier transform phase uncorrected of the k^3^-weighted spectra of Pd_1_Zn_1_/TiO_2_ (green) and Pd_1_Zn_20_/TiO_2_ (orange) collected in situ after reduction in 5% H_2_ in Ar at 400 °C, along with reference spectra from Pd metal foil (orange dots) and PdO (blue dots). (c,d) Zn K-edge XANES spectra of (c) Pd_1_Zn_1_/TiO_2_ and (d) Pd_1_Zn_20_/TiO_2_, collected in situ before and after reduction in 5% H_2_ in Ar at 400 °C.

The equivalent Zn K-edge XANES spectra are presented in Figure 4c and d, which also compares spectra from prereduction and postreduction samples. We note that the white line positions for the Zn foil and ZnO reference spectra are very similar, but that the higher energy region of the spectra show clearer differences between metallic and oxidized states. The XANES spectra for the prereduction Pd_1_Zn_1_/TiO_2_ sample shown in Figure 4c has a white line intensity intermediate between the Zn foil and ZnO references; however, the higher energy features follow closely the ZnO reference. The spectra for postreduction Pd_1_Zn_1_/TiO_2_ is very similar in the white line region but, at higher energies, has features indicative of Zn in the metal state, for example at ca. 9700 eV. This is consistent with the majority Zn species being incorporated into the metallic alloy phase. The prereduction Pd_1_Zn_20_/TiO_2_ spectra (Figure 4d) show a white line intensity similar to Zn foil with a slight red shift, possibly as a result of the presence of some reduced Zn and/or oxygen vacancies in ZnO.^27^ However, at higher energies, the spectra follow closely the ZnO reference. There is no effect of reduction on the XANES spectra for Pd_1_Zn_20_/TiO_2_. These Zn XANES spectra further support the observation of alloy formation when this system is subjected to a reductive atmosphere and high temperatures, which was also concluded from the Pd K-edge spectrum (Figure 4a).

In summary, for both catalyst compositions, the XANES spectra are indicative of alloying between Pd and Zn, which becomes more prominent at high Zn loadings. In addition, for Pd_1_Zn_1_/TiO_2_, the major Zn environment is within the metal alloy, while for Pd_1_Zn_20_/TiO_2_, the majority of Zn is present as ZnO. This is consistent with alloy particles forming at both catalyst compositions. When Zn loadings are higher than that required for the 1:1 stoichiometry of the β-PdZn alloy, the excess Zn is present as ZnO, and this dominates the XANES spectra for Pd_1_Zn_20_/TiO_2_. The formation of ZnO at the higher Zn:Pd ratios will also lead to the creation of a significant interfacial area between alloy and ZnO.

## CO_2_ Activation and Hydrogenation—DFT Calculations

To obtain insight into the origin of the chemical selectivity and activity of the catalysts, computational simulation has been applied using the periodic DFT approach given in the Materials and Methods section and Supporting Information Section S3.

The major products seen in the experimental work presented here are CO (from RWGS; eq 5) and methanol. Over Pd surfaces, we have previously found that CO_2_ hydrogenation to formate is the preferred initial step in the hydrogenation of CO_2_ to methanol. Accordingly, the branching between these products, which controls selectivity to methanol, can take place following the elementary steps:

9 $${CO}_{2}\left(a\right)\to CO\left(a\right)+O\left(a\right)$$

10 $${CO}_{2}\left(a\right)+H\left(a\right)\to HCOO\left(a\right)$$

where “(a)” refers to adsorbed species. Equation 9 is the dissociation of CO_2_ to produce CO­(a) and an adsorbed oxygen atom, which is subsequently hydrogenated to complete the RWGS reaction. Equation 10 shows the hydrogenation of CO_2_(a) to produce adsorbed formate, which is further hydrogenated to methanol. Our DFT calculations cover these two steps and the preceding adsorption of H_2_(g) and CO_2_(g) over relevant model systems of Pd and β-PdZn surfaces. We also consider the adsorption of C*O*_2_(*g*) on the ZnO component of the catalyst found when Zn is in excess, which leads to the most productive catalysts.

### Hydrogen Adsorption

The interaction of hydrogen with the Pd and PdZn surfaces was studied by calculating the adsorption energy of a hydrogen atom relative to 1/2 H_2_(*g*), with the H atom adsorbed in each of the symmetry unique adsorption sites shown in Figures S1 and S2. The details of these calculations are given in the Supporting Information (Sections S3 and S7, Table S5).

On the Pd(110) surface, the most favorable site is the face-centered cubic (FCC) site (*E*_ads_(H) = −0.47 eV), in which H has the maximum number of Pd neighbors for the surface (three). This result is in good agreement with previous work by Dong et al., who find *E*_ads_(H) = −0.50 eV on the same surface.^53^ In contrast, on Zn metal surfaces, others have found hydrogen adsorption to be highly endothermic.^54^ The only site on β-PdZn surfaces offering a significantly negative energy for hydrogen atom adsorption is the Pd_2_Zn hollow site on β-PdZn(101), which is designated as site G in Figure S2b. At this site, the H atom sits in a bridge-like configuration, coordinated to two Pd atoms and has a calculated *E*_ads_(H) of −0.25 eV. At all other sites, the co-ordination environment either involves more Zn atoms and single Pd atoms or the arrangement of surface atoms must distort to interact with the adsorbate, leading to positive or near-zero *E*_ads_(H) values (Table S5).

### CO_2_ Adsorption on Pd and PdZn

We studied two binding modes for CO_2_ on Pd and β-PdZn surfaces. First, a weakly exothermic physisorbed structure with *E*_ads_ values of −0.14 to −0.17 eV is observed. In the optimized physisorbed structure, the molecule is around 3.3 Å from the surface in a horizontal, linear geometry. Second, we find a bidentate chemisorbed geometry, with the carbon atom and one oxygen atom each interacting with a different surface metal atom. Bidentate chemisorption results in a nonlinear structure with O–C–O angles significantly away from 180°.^31^ In this case, there is also a partial negative charge transfer from Pd to the C atom to produce the CO_2_^δ−^. Despite the closer interaction with the surface, the calculated *E*_ads_ of the CO_2_^δ−^ species on Pd is quite weak and is found to be endothermic (0.15 eV) on Pd(111) and only mildly exothermic on Pd(100) and Pd(110) (−0.08 eV and −0.16 eV, respectively). On β-PdZn surfaces, the bidentate CO_2_^δ−^ species binds on intermetallic bridging sites, with C preferentially binding to Pd and O coordinating with Zn, as would be expected from the charge distribution in the alloy (Figure 5a,b). However, the chemisorption of CO_2_^δ−^ is endothermic on both β-PdZn(101) and β-PdZn(110) surfaces, with *E*_ads_ of 0.22 and 0.18 eV, respectively. This matches previous calculations on the same system performed using a different density functional.^31^ Accordingly, CO_2_^δ−^ is probably a very short-lived intermediate on the pathway between CO_2_ and formate (first hydrogenation intermediate) or CO­(*a*) + O­(*a*) (part of RWGS). For completeness, we also considered CO_2_ adsorption to the Zn(0001) surface. The calculation for Zn(0001) gave *E*_ads_ = −0.10 eV, with no chemisorption, matching the experimentally observed limited interaction of CO_2_ with metallic Zn unless at high electrostatic potential.^55,56^

**Figure 5:**
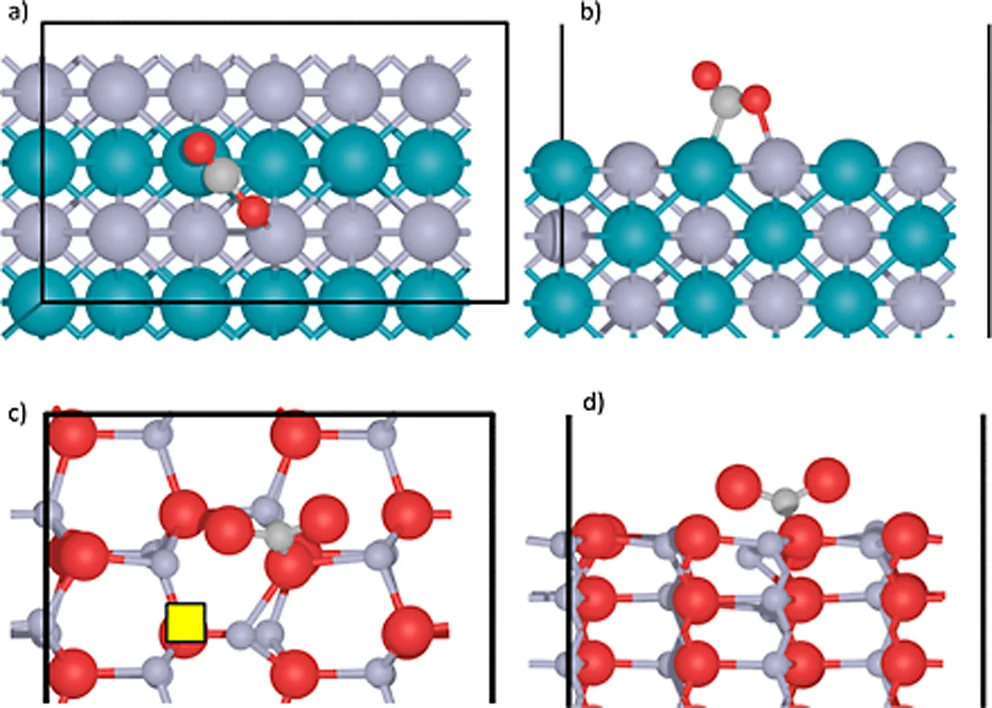
Calculated chemisorbed structure *of* CO_2_ on the β-PdZn(110) surface in (a) plan view and (b) side view and on the ZnO (112̅0) surface in (c) plan view and (d) side view. Atom colors: C, light gray; Zn, dark gray; Pd, blue; and O, red; the black lines show periodic boundaries, and in (c), the yellow square shows the location of the oxygen defect.

The highest CO_2_ conversions and selectivities for methanol in the experimental studies here occur for catalyst compositions in which Zn is present in excess compared to that of Pd (Figure 1). These catalysts have PdZn alloy particles in contact with ZnO. Accordingly, the role of ZnO in CO_2_ adsorption was also investigated using DFT calculations of CO_2_ on stoichiometric ZnO nonpolar surfaces. The effect on the adsorption of introducing a single oxygen anion vacancy on the surfaces was also considered. The adsorption of CO_2_ on the nonpolar surfaces of ZnO to form a surface carbonate (CO_3_^2–^) species was found to give a more favorable adsorption energy than seen on Pd or β-PdZn surfaces. For the stoichiometric surfaces, *E*_ads_ values of −0.21 eV and −1.14 eV were calculated for the ZnO (112̅0) and the ZnO (101̅0) surfaces, respectively.

Defect formation energies, *E*_def_(H_2_O–H_2_), were calculated with respect to reaction of surface oxygen with *H*_2_(*g*) to produce H_2_O. For this process, we obtain *E*_def_(H_2_O–H_2_) = 0.60 eV for the ZnO (112̅0) surface and *E*_def_(H_2_O–H_2_) = 1.28 and 0.76 eV for site A and site B (Figure S3) on ZnO (101̅0), respectively. When a defect is introduced, the calculated adsorption energy for CO_2_ is enhanced further. On the ZnO (112̅0), the carbonate forms on an oxygen anion neighboring the vacancy (Figure 5c,d), giving a calculated adsorption energy of −0.78 eV, almost four times more favorable than seen for the corresponding stoichiometric surface. For the ZnO (101̅0), the enhancement of the CO_2_ adsorption is marginal with calculated *E*_ads_ values of −1.21 eV and −1.19 eV with defects at site A and site B (Figure S3), respectively. In these cases, the optimization also results in the carbonate sitting close to the defect location.

These calculations suggest that there will be C_1_ carbon species adsorbed on the ZnO component of the catalyst that could act as a reservoir of reactive species in the methanol synthesis pathway, as has been suggested for methanol synthesis on the polar ZnO(0001) surfaces in earlier DFT studies.^57^ The availability of hydrogen atoms adsorbed on the metal may then enhance the hydrogenation of the C_1_ species to formate at the interface between the oxide and the metal nanoparticles.

### CO_2_ Reaction over Pd and β-PdZn Surfaces

Table 1 shows the calculated forward and reverse barriers for the elementary steps shown in eqs 9 and 10. Over the Pd(111) and Pd(100) surfaces, the RWGS forward barrier is around half that of the hydrogenation reaction. For Pd(110), the barriers for both reactions are relatively high. Pd(111) and Pd(100) are the major facets of Pd nanoparticles at the sizes observed here, which could explain the observed high selectivity toward CO for the Pd/TiO_2_- and Pd-rich catalysts (Figure 1). In contrast, for the β-PdZn surfaces, the forward barrier for the hydrogenation of CO_2_ to formate is much lower than that for the RWGS, again consistent with the higher selectivity toward methanol for catalysts in which the Zn content is sufficient for β-PdZn formation.

**Table 1:** Calculated Barriers for Forward and Reverse Elementary Reactions of CO_2_

surface	*E*_act_^fwd^ (RWGS)/eV^a^	*E*_act_^rev^ (RWGS)/eV	*E*_act_^fwd^ (hyd)/eV	*E*_act_^rev^ (hyd)/eV
Pd(111)	0.62	1.55	1.32	0.99
Pd(100)	0.66	0.78	1.13	1.02
Pd(110)	1.74	1.97	1.00	0.96
PdZn(101)	1.05	1.39	0.73	1.15
PdZn(110)	1.02	1.61	0.61	1.36

a Note: RWGS: CO_2_ dissociation for reverse water gas shift, eq 9; hyd: hydrogenation of CO_2_ to formate, eq 10. In both cases, superscript fwd: forward and rev: reverse reaction direction.

## Discussion of Mechanism

The mechanism of methanol synthesis on oxide supported metals has been widely debated previously, with general support for mechanisms in which methanol is formed via an intermediate formate species.^54,57–59^ In the particular case of catalysts containing both Pd and Zn, an excess of Zn has been linked to the formation of ZnO overlayers on PdZn alloy nanoparticles during the initial hours of the CO_2_ hydrogenation reaction, after which the catalyst achieves a stable activity.^22–24,28^ Several researchers have previously proposed that this ZnO component is responsible for the adsorption of CO_2_ in PdZn catalysts and that CO_2_ hydrogenation happens either at the metal–support interface (MSI) or on the ZnO overlayer via hydrogen spillover from the metal alloy.^16,18,22–24,28,60^ The enhanced CO_2_ adsorption has also been identified as an important role of ZnO observed for the Cu/ZnO catalysts.^61,62^

In the current work, characterization data show that changing the Zn loading used in synthesis gives roughly three classes of catalysts: (i) Pd/TiO_2_, (ii) low Zn content Pd_*x*_Zn_*y*_/TiO_2_ (0 < Zn:Pd ≤ 1), and (iii) high Zn content Pd_*x*_Zn_*y*_/TiO_2_ (Zn:Pd > 2). Electron microscopy (Figure 3) and XAS (Figure 4) confirmed that Pd_1_Zn_1_/TiO_2_ does contain PdZn binary phases and very little ZnO, while there is a significant ZnO content for high Zn content catalysts.

Pd is an effective hydrogenation catalyst due to the ease with which H_2_ is dissociated on the surface of Pd nanoparticles. In addition, CO has a very favorable adsorption energy on the three major surfaces of Pd (*E*_ads_(CO) ≈ −2.00 eV, Table S4), meaning that the CO product from the RWGS on the metal particle component of Pd/TiO_2_ will have a long lifetime, as supported by recent work by Qillis Romero et al.^38^ Methanation has the highest hydrogen demand of the three reactions considered for product formation from CO_2_ (methanol, eq 1, methane, eq 2, and CO, eq 3), and we have proposed that the methanation reaction proceeds through CO via surface carbon on the metal component of the catalyst (eqs 5–7). This process will be favored by high surface concentration of hydrogen and availability of surface sites with high Pd co-ordination. On the β-PdZn surfaces, only 2-fold co-ordination to Pd is possible and the DFT calculated adsorption energies of hydrogen and of CO (*E*_ads_(CO) = −1.10 eV (Table S6) and *E*_ads_(H) = −0.25 eV (Table S5)) are around half of the values seen over Pd. Accordingly, for the low Zn loading catalysts, addition of Zn would be expected to reduce the availability of H_2_ and the affinity of the surface for CO, which leads to lower selectivity to methane as the Zn level is increased from Zn:Pd = 0 to 1 (Figure 1). It is interesting that for all the catalysts in this composition range, the combined selectivity to methane and CO is 92 ± 5%, while the methane selectivity drops from 17% to 1%, consistent with the idea that the RWGS pathway leads to both methane and CO, with the methanation pathway becoming less important as the PdZn alloy is formed.

The most selective catalysts for methanol synthesis are the Pd_*x*_Zn_*y*_/TiO_2_ catalysts with high Zn loadings, Zn:Pd > 2 (Figure 1). The DFT calculations presented here show that CO_2_ adsorbs very weakly on the surfaces of Pd and β-PdZn, but it can adsorb strongly as carbonate on ZnO, in agreement with the idea that ZnO is responsible for the adsorption of CO_2_. Indeed, both CO_2_ conversion and methanol selectivity are seen to be higher for catalysts compositions with Zn in excess (Zn:Pd > 1.5) than for Pd_1_Zn_1_/TiO_2_. For Pd_1_Zn_3_/TiO_2_, methanol selectivity is comparable to CO selectivity at around 50%. This agrees with the idea that surface coverage of ZnO is beneficial for CO_2_ adsorption at the MSI where it can be hydrogenated to formate. Zabilskiy et al.^27^ have employed FTIR spectroscopy and XAS analysis for Pd/ZnO catalysts and also found that formate species are bound to Zn at the MSI. Therefore, the interface between PdZn nanoparticles and ZnO is the probable site for reactions selective for methanol. We note that the CO selectivity is lower when methanol is present as a reaction product than when the Pd/TiO_2_ catalyst or low Zn containing materials are used. This suggests that methanol and CO production are competing products rather than CO being produced from methanol.

To summarize, it is proposed that once the CO_2_ is chemically activated on ZnO and in the vicinity of H activated on PdZn at the MSI, the hydrogenation to the methanol precursor, i.e., formate, can occur. Such activated formate species anchored at the MSI could benefit from the low activation energy barriers toward hydrogenation, which aligns with other investigations^4,18,27,28,63–66^ for different methanol synthesis catalysts involving metal oxide supports. The decrease in methane production is considered to be due to the ensembles of Pd required for CO dissociation being effectively blocked by Zn atoms through alloying with Pd at the surface to form the 1:1 β-PdZn alloy. In addition, the lower supply of hydrogen in the alloy system is essential to form methane.

## Conclusions

This study provides insight into the effect of varying Pd and Zn molar ratios in a PdZn/TiO_2_ catalyst for CO_2_ hydrogenation. Pd/TiO_2_ produces CO as the major product with lower amounts of CH_4_ and CH_3_OH. Even at low Zn loadings, below that required for stoichiometric β-PdZn formation, the presence of the PdZn alloy phase is confirmed and reduces CH_4_ production by reducing the level of CO dissociation and hydrogen surface coverage on the metal. At the stoichiometric 1:1 PdZn ratio, CH_4_ production is very low, and there is also very little methanol production (<∼5%). The selectivity shifts toward methanol only when the Zn:Pd ratio exceeds 1, and at this point, both 1:1 β-PdZn alloy and ZnO are present. DFT reveals that CO_2_ adsorbs weakly on Pd and PdZn surfaces and strongly on some ZnO surfaces. Through the combination of experimental data and computational simulations, we propose that once CO_2_ is activated on ZnO, it reacts with hydrogen supplied by the metal nanoparticle to form the formate intermediate. This intermediate can be stabilized at the metal–support interface, indicating the importance of having both the 1:1 β-PdZn and ZnO present for methanol synthesis.

## Supplementary Material



## Notes

Reactor and catalyst testing methodology, characterization (XRD and XAS) methodology, properties of catalysts, computational methods and visualization of structures, catalyst testing data, XPS analysis, electron microscopy measurements, computational results, and quantification and schematic of PdZn alloy formation (PDF)

## References

[1] IEA . CO_2_ Emissions—Global Energy Review 2025—Analysis. https://www.iea.org/reports/global-energy-review-2025/co2-emissions (accessed June 03, 2025).

[2] Schlögl R. (2010). The Role of
Chemistry in the Energy Challenge. ChemSusChem.

[3] Nguyen V.-H., Nguyen B.-S., Jin Z., Shokouhimehr M., Jang H. W., Hu C., Singh P., Raizada P., Peng W., Shiung Lam S., Xia C., Nguyen C. C., Kim S. Y., Le Q. V. (2020). Towards Artificial Photosynthesis:
Sustainable Hydrogen Utilization for Photocatalytic Reduction of CO_2_ to High-Value Renewable Fuels. Chem.
Eng. J..

[4] Kattel S., Ramírez P. J., Chen J. G., Rodriguez J. A., Liu P. (2017). Active Sites for CO_2_ Hydrogenation to Methanol on Cu/ZnO
Catalysts. Science.

[5] Wang J., Liu H., Wang T., Xi Y., Sun P., Li F. (2023). Boosting CO_2_ Hydrogenation to Methanol via Cu-Zn Synergy over Highly Dispersed
Cu,Zn-Codoped ZrO_2_ Catalysts. Catal.
Today.

[6] Chu Z., Chen H., Yu Y., Wang Q., Fang D. (2013). Surfactant-Assisted
Preparation of Cu/ZnO/Al_2_O_3_ Catalyst for Methanol
Synthesis from Syngas. J. Mol. Catal. Chem..

[7] Liu X.-M., Lu G. Q., Yan Z.-F., Beltramini J. (2003). Recent Advances
in Catalysts for Methanol Synthesis via Hydrogenation of CO and CO_2_. Ind. Eng. Chem. Res..

[8] Alper E., Yuksel Orhan O. (2017). CO_2_ Utilization: Developments in Conversion
Processes. Petroleum.

[9] Sun J. T., Metcalfe I. S., Sahibzada M. (1999). Deactivation of Cu/ZnO/Al_2_O_3_ Methanol Synthesis Catalyst by Sintering. Ind. Eng. Chem. Res..

[10] Liang B., Ma J., Su X., Yang C., Duan H., Zhou H., Deng S., Li L., Huang Y. (2019). Investigation on Deactivation
of Cu/ZnO/Al_2_O_3_ Catalyst for CO_2_ Hydrogenation
to Methanol. Ind. Eng. Chem. Res..

[11] Prašnikar A., Pavlišič A., Ruiz-Zepeda F., Kovač J., Likozar B. (2019). Mechanisms of Copper-Based Catalyst
Deactivation during CO_2_ Reduction to Methanol. Ind. Eng. Chem. Res..

[12] Kasatkin I., Kurr P., Kniep B., Trunschke A., Schlögl R. (2007). Role of Lattice Strain and Defects in Copper Particles
on the Activity of Cu/ZnO/Al_2_O_3_ Catalysts for
Methanol Synthesis. Angew. Chem..

[13] Bahruji H., Armstrong R. D., Ruiz Esquius J., Jones W., Bowker M., Hutchings G. J. (2018). Hydrogenation of CO_2_ to Dimethyl Ether over
Brønsted Acidic PdZn Catalysts. Ind. Eng.
Chem. Res..

[14] Toyir J., Miloua R., Elkadri N. E., Nawdali M., Toufik H., Miloua F., Saito M. (2009). Sustainable Process for the Production
of Methanol from CO_2_ and H_2_ Using Cu/ZnO-Based
Multicomponent Catalyst. Phys. Procedia.

[15] Lawes N., Aggett K. J., Smith L. R., Slater T. J. A., Dearg M., Morgan D. J., Dummer N. F., Taylor S. H., Hutchings G. J., Bowker M. (2024). Zn Loading Effects on the Selectivity of PdZn Catalysts
for CO_2_ Hydrogenation to Methanol. Catal. Lett..

[16] Bahruji H., Bowker M., Hutchings G., Dimitratos N., Wells P., Gibson E., Jones W., Brookes C., Morgan D., Lalev G. (2016). Pd/ZnO Catalysts for Direct CO_2_ Hydrogenation to Methanol. J. Catal..

[17] Lawes N., Gow I. E., Smith L. R., Aggett K. J., Hayward J. S., Kabalan L., Logsdail A. J., Slater T. J. A., Dearg M., Morgan D. J., Dummer N. F., Taylor S. H., Bowker M., Catlow C. R. A., Hutchings G. J. (2023). Methanol Synthesis from CO_2_ and H_2_ Using Supported Pd Alloy Catalysts. Faraday Discuss..

[18] Bowker M., Lawes N., Gow I., Hayward J., Esquius J. R., Richards N., Smith L. R., Slater T. J. A., Davies T. E., Dummer N. F., Kabalan L., Logsdail A., Catlow R. C., Taylor S., Hutchings G. J. (2022). The Critical Role of β-PdZn
Alloy in Pd/ZnO Catalysts for the Hydrogenation of Carbon Dioxide
to Methanol. ACS Catal..

[19] Bahruji H., Bowker M., Jones W., Hayward J., Ruiz Esquius J., Morgan D. J., Hutchings G. J. (2017). PdZn Catalysts for CO_2_ Hydrogenation to Methanol Using Chemical Vapour Impregnation (CVI). Faraday Discuss..

[20] Peterson E. J., Halevi B., Kiefer B., Spilde M. N., Datye A. K., Peterson J., Daemen L., Llobet A., Nakotte H. (2011). Aerosol Synthesis
and Rietveld Analysis of Tetragonal (Β1) PdZn. J. Alloys Compd..

[21] Miyazaki M., Furukawa S., Takayama T., Yamazoe S., Komatsu T. (2019). Surface Modification
of PdZn Nanoparticles via Galvanic Replacement for the Selective Hydrogenation
of Terminal Alkynes. ACS Appl. Nano Mater..

[22] Heggen M., Penner S., Friedrich M., Dunin-Borkowski R. E., Armbrüster M. (2016). Formation of ZnO Patches on ZnPd/ZnO during Methanol
Steam Reforming: A Strong Metal–Support Interaction Effect?. J. Phys. Chem. C.

[23] Friedrich M., Penner S., Heggen M., Armbrüster M. (2013). High CO_2_ Selectivity in Methanol Steam Reforming through ZnPd/ZnO
Teamwork. Angew. Chem., Int. Ed..

[24] Nowicka E., Althahban S. M., Luo Y., Kriegel R., Shaw G., Morgan D. J., He Q., Watanabe M., Armbrüster M., Kiely C. J., Hutchings G. J. (2018). Highly Selective PdZn/ZnO Catalysts
for the Methanol Steam Reforming Reaction. Catal.
Sci. Technol..

[25] Crawley J. W. M., Gow I. E., Lawes N., Kowalec I., Kabalan L., Catlow C. R. A., Logsdail A. J., Taylor S. H., Dummer N. F., Hutchings G. J. (2022). Heterogeneous Trimetallic Nanoparticles as Catalysts. Chem. Rev..

[26] Bowker M., Houghton H., Waugh K. C. (1981). Mechanism and Kinetics of Methanol
Synthesis on Zinc Oxide. J. Chem. Soc. Faraday
Trans. 1 Phys. Chem. Condens. Phases.

[27] Zabilskiy M., Sushkevich V. L., Newton M. A., Krumeich F., Nachtegaal M., van Bokhoven J. A. (2021). Mechanistic Study of Carbon Dioxide Hydrogenation over
Pd/ZnO-Based Catalysts: The Role of Palladium–Zinc Alloy in
Selective Methanol Synthesis. Angew. Chem.,
Int. Ed..

[28] Quilis C., Mota N., Pawelec B., Millán E., Navarro Yerga R. M. (2023). Intermetallic PdZn/TiO_2_ Catalysts for Methanol
Production from CO_2_ Hydrogenation: The Effect of ZnO Loading
on PdZn-ZnO Sites and Its Influence on Activity. Appl. Catal. B Environ..

[29] Higham M. D., Quesne M. G., Catlow C. R. A. (2020). Mechanism of CO_2_ Conversion
to Methanol over Cu(110) and Cu(100) Surfaces. Dalton Trans..

[30] Kabalan L., Kowalec I., Catlow C. R. A., Logsdail A. J. (2021). A Computational
Study of the Properties of Low- and High-Index Pd, Cu and Zn Surfaces. Phys. Chem. Chem. Phys..

[31] Kowalec I., Kabalan L., Catlow C. R. A., Logsdail A. J. (2022). A Computational
Study of Direct CO_2_ Hydrogenation to Methanol on Pd Surfaces. Phys. Chem. Chem. Phys..

[32] van
Beek W., Safonova O. V., Wiker G., Emerich H. (2011). SNBL, a Dedicated Beamline
for Combined in Situ X-Ray Diffraction, X-Ray Absorption and Raman
Scattering Experiments. Phase Transit..

[33] Blum V., Gehrke R., Hanke F., Havu P., Havu V., Ren X., Reuter K., Scheffler M. (2009). *Ab Initio* Molecular
Simulations with Numeric Atom-Centered Orbitals. Comput. Phys. Commun..

[34] Hjorth
Larsen A., Jørgen Mortensen J., Blomqvist J., Castelli I. E., Christensen R., Dułak M., Friis J., Groves M. N., Hammer B., Hargus C., Hermes E. D., Jennings P. C., Bjerre Jensen P., Kermode J., Kitchin J. R., Leonhard Kolsbjerg E., Kubal J., Kaasbjerg K., Lysgaard S., Bergmann
Maronsson J., Maxson T., Olsen T., Pastewka L., Peterson A., Rostgaard C., Schiøtz J., Schütt O., Strange M., Thygesen K. S., Vegge T., Vilhelmsen L., Walter M., Zeng Z., Jacobsen K. W. (2017). The Atomic
Simulation Environment Python Library for Working with Atoms. J. Phys.: Condens. Matter.

[35] Chan C. T., Bohnen K. P., Ho K. M. (1993). Accelerating the Convergence of Force
Calculations in Electronic-Structure Computations. Phys. Rev. B.

[36] Hansen M. H., Torres J. A. G., Jennings P. C., Wang Z., Boes J. R., Mamun O. G., Bligaard T. (2019). An Atomistic Machine Learning Package
for Surface Science and Catalysis. arXiv.

[37] Henkelman G., Uberuaga B. P., Jónsson H. (2000). A Climbing Image Nudged Elastic Band
Method for Finding Saddle Points and Minimum Energy Paths. J. Chem. Phys..

[38] Quilis
Romero C., Mota Toledo N., Pawelec B., Navarro
Yerga R. M. (2025). Unveiling Key Factors Governing the Activity of Pd/TiO_2_ Catalysts in the Low-Temperature Reverse Water–Gas
Shift Reaction. ChemCatChem.

[39] Wei W., Jinlong G. (2011). Methanation of Carbon Dioxide: An Overview. Front. Chem. Sci. Eng..

[40] Kwak J. H., Kovarik L., Szanyi J. (2013). Heterogeneous Catalysis on Atomically
Dispersed Supported Metals: CO_2_ Reduction on Multifunctional
Pd Catalysts. ACS Catal..

[41] Tew M. W., Emerich H., van Bokhoven J. A. (2011). Formation and Characterization of
PdZn Alloy: A Very Selective Catalyst for Alkyne Semihydrogenation. J. Phys. Chem. C.

[42] Yin Y., Hu B., Li X., Zhou X., Hong X., Liu G. (2018). Pd@zeolitic
Imidazolate Framework-8 Derived PdZn Alloy Catalysts for Efficient
Hydrogenation of CO_2_ to Methanol. Appl. Catal. B Environ..

[43] Iwasa N., Takezawa N. (2003). New Supported Pd and Pt Alloy Catalysts for Steam Reforming
and Dehydrogenation of Methanol. Top. Catal..

[44] Eblagon K. M., Concepción P. H., Silva H., Mendes A. (2014). Ultraselective Low
Temperature Steam Reforming of Methanol over PdZn/ZnO Catalysts Influence
of Induced Support Defects on Catalytic Performance. Appl. Catal. B Environ..

[45] Bahruji H., Esquius J. R., Bowker M., Hutchings G., Armstrong R. D., Jones W. (2018). Solvent Free Synthesis of PdZn/TiO_2_ Catalysts for the Hydrogenation of CO_2_ to Methanol. Top. Catal..

[46] Liu S., Takahashi K., Fuchigami K., Uematsu K. (2006). Hydrogen Production
by Oxidative Methanol Reforming on Pd/ZnO: Catalyst Deactivation. Appl. Catal. Gen..

[47] Forde M. M., Kesavan L., bin Saiman M. I., He Q., Dimitratos N., Lopez-Sanchez J. A., Jenkins R. L., Taylor S. H., Kiely C. J., Hutchings G. J. (2014). High Activity Redox Catalysts Synthesized by Chemical
Vapor Impregnation. ACS Nano.

[48] Kanuri S., Roy S., Chakraborty C., Datta S. P., Singh S. A., Dinda S. (2022). An Insight
of CO_2_ Hydrogenation to Methanol Synthesis: Thermodynamics,
Catalysts, Operating Parameters, and Reaction Mechanism. Int. J. Energy Res..

[49] Ojelade O. A., Zaman S. F., Daous M. A., Al-Zahrani A. A., Malik A. S., Driss H., Shterk G., Gascon J. (2019). Optimizing
Pd:Zn Molar Ratio in PdZn/CeO_2_ for CO_2_ Hydrogenation
to Methanol. Appl. Catal. Gen..

[50] Föttinger K., van Bokhoven J. A., Nachtegaal M., Rupprechter G. (2011). Dynamic Structure
of a Working Methanol Steam Reforming Catalyst: In Situ Quick-EXAFS
on Pd/ZnO Nanoparticles. J. Phys. Chem. Lett..

[51] Childers D. J., Schweitzer N. M., Shahari S. M. K., Rioux R. M., Miller J. T., Meyer R. J. (2014). Modifying Structure-Sensitive Reactions by Addition
of Zn to Pd. J. Catal..

[52] Veligzhanin A. A., Zubavichus Ya. V., Kozitsyna N. Yu., Murzin V. Yu., Khramov E. V., Chernyshov A. A. (2013). Investigation of PdZn Nanoparticle Formation upon the
Thermal Decomposition of Acetate Precursors by in Situ XRD and XAFS. J. Surf. Investig. X-Ray Synchrotron Neutron Technol..

[53] Vitos L., Ruban A. V., Skriver H. L., Kollár J. (1998). The Surface
Energy of Metals. Surf. Sci..

[54] Bowker M. (2019). Methanol Synthesis
from CO_2_ Hydrogenation. ChemCatChem.

[55] Kuhl K. P., Hatsukade T., Cave E. R., Abram D. N., Kibsgaard J., Jaramillo T. F. (2014). Electrocatalytic Conversion of Carbon Dioxide to Methane
and Methanol on Transition Metal Surfaces. J.
Am. Chem. Soc..

[56] Blaziak K., Tzeli D., Xantheas S. S., Uggerud E. (2018). The Activation of Carbon
Dioxide by First Row Transition Metals (Sc–Zn). Phys. Chem. Chem. Phys..

[57] Medford A. J., Sehested J., Rossmeisl J., Chorkendorff I., Studt F., Nørskov J. K., Moses P. G. (2014). Thermochemistry
and Micro-Kinetic Analysis of Methanol Synthesis on ZnO (0001). J. Catal..

[58] Jiang X., Wang X., Nie X., Koizumi N., Guo X., Song C. (2018). CO_2_ Hydrogenation to Methanol on Pd-Cu Bimetallic Catalysts:
H_2_/CO_2_ Ratio Dependence and Surface Species. Catal. Today.

[59] Larmier K., Liao W.-C., Tada S., Lam E., Verel R., Bansode A., Urakawa A., Comas-Vives A., Copéret C. (2017). CO_2_-to-Methanol Hydrogenation on Zirconia-Supported
Copper Nanoparticles: Reaction Intermediates and the Role of the Metal–Support
Interface. Angew. Chem., Int. Ed..

[60] Song J., Liu S., Yang C., Wang G., Tian H., Zhao Z., Mu R., Gong J. (2020). The Role of Al Doping in Pd/ZnO Catalyst for CO_2_ Hydrogenation to Methanol. Appl. Catal.
B Environ..

[61] Aziz F. F. A., Timmiati S. N., Jalil A. A., Rusdan N. A., Annuar N. H. R., Teh L. P. (2024). Recent Innovation on Heterogeneous ZnO-Based Catalysts
for Enhanced CO2 Hydrogenation. J. Environ.
Chem. Eng..

[62] Han C., Qin L., Wang P., Zhang H., Gao Y., Zhu M., Wang S., Li J. (2024). Tuning CO2 Hydrogenation Selectivity
via Support Interface Types on Cu-Based Catalysts. Fuel.

[63] Lorenz H., Rameshan C., Bielz T., Memmel N., Stadlmayr W., Mayr L., Zhao Q., Soisuwan S., Klötzer B., Penner S. (2013). From Oxide-Supported Palladium to Intermetallic Palladium
Phases: Consequences for Methanol Steam Reforming. ChemCatChem.

[64] Wang H., Zhang G., Fan G., Yang L., Li F. (2021). Fabrication
of Zr–Ce Oxide Solid Solution Surrounded Cu-Based Catalyst
Assisted by a Microliquid Film Reactor for Efficient CO_2_ Hydrogenation to Produce Methanol. Ind. Eng.
Chem. Res..

[65] Senanayake S. D., Ramírez P. J., Waluyo I., Kundu S., Mudiyanselage K., Liu Z., Liu Z., Axnanda S., Stacchiola D. J., Evans J., Rodriguez J. A. (2016). Hydrogenation of CO_2_ to
Methanol on CeOx/Cu(111) and ZnO/Cu(111) Catalysts: Role of the Metal–Oxide
Interface and Importance of Ce^3+^ Sites. J. Phys. Chem. C.

[66] Palomino R. M., Ramírez P. J., Liu Z., Hamlyn R., Waluyo I., Mahapatra M., Orozco I., Hunt A., Simonovis J. P., Senanayake S. D., Rodriguez J. A. (2018). Hydrogenation of CO_2_ on
ZnO/Cu(100) and ZnO/Cu(111) Catalysts: Role of Copper Structure and
Metal–Oxide Interface in Methanol Synthesis. J. Phys. Chem. B.

