# Supplementary material for: The Important Role of Alloy–Oxide Interfaces in Controlling Methanol Formation in CO2 Hydrogenation
Source: ACS Catal. 2026 Jan 13;16(3):2209–21. doi: 10.1021/acscatal.5c06703 (PMC13730434; doi:10.1021/acscatal.5c06703)
Supplement: Supplementary file 1 [file cs5c06703_si_001.pdf]

Supporting Information

The important role of alloy-oxide interfaces in controlling methanol formation in CO_2 hydrogenation.

Naomi Lawes,[a] Igor Kowalec,[a] Sofia Mediavilla-Madrigal,[a][b] Kieran J. Aggett,[a] Louise R. Smith,[a] Malcolm Dearg,[c] Thomas J. A. Slater,[a] Eimear McCarthy,[a] Herzain I. Rivera-Arrieta,[d] Matthias Scheffler,[d] David J. Morgan,[a][e] David J. Willock,[a] Andrew M. Beale,[b][f] Andrew J. Logsdail,[a] Nicholas F. Dummer,[a] Michael Bowker,[a]* C. Richard A. Catlow,[a][b][f] Stuart H. Taylor,[a] and Graham J. Hutchings.[a]*

[a] Max Planck-Cardiff Centre on the Fundamentals of Heterogeneous Catalysis FUNCAT, Translational Research Hub, Cardiff University, Maindy Road, Cardiff CF24 4HQ, UK

[b] UK Catalysis Hub, Research Complex at Harwell, Rutherford Appleton Laboratory, Didcot, OX11 0FA, UK

[c] School of Physics, Engineering and Technology, University of York, York YO10 5DD, United Kingdom

[d] The NOMAD Laboratory at the Fritz-Haber-Institut of the Max-Planck-Gesellschaft and IRIS-Adlershof of the Humboldt-Universität zu Berlin, Germany.

[e] HarwellXPS – EPSRC National Facility for Photoelectron Spectroscopy, Research Complex at Harwell (RCaH), Didcot, OX11 0FA, UK

[f] Chemistry Department, University College London, 20 Gordon Street, London, WC1H 0AJ, UK

*Corresponding authors: bowkerm@cardiff.ac.uk, hutch@cardiff.ac.uk

Supporting Information contains 27 pages (S1 to S26)

It has 8 sections comprised of 6 supporting tables (Table S1 to Table S6) and 12 supporting figures (Figure S1 to Figure S12)

**Section**	**Description/Tables/Figures**	**Page**
**S1 Reactor and Catalytic Testing**	The reactor and operation	S3
**S2 Characterisation Methodology**	XRD and XAS methodology	S4
Table S1.	Properties of TiO_2, Pd/TiO_2 and Pd_xZn_y/TiO_2.	S9
**S3 Computational Methods**	Calculations used for DFT	S5
Figure S1.	A ball and stick visualisation of face-centred cubic Pd surfaces showing adsorption sites considered in the study.	S9
Figure S2.	A ball and stick visualisation of body-centred tetragonal β-PdZn surfaces showing adsorption sites considered in the study.	S10
Figure S3.	A ball and stick visualisation of slabs used for simulation of ZnO surfaces with vacancy sites considered in the study marked.	S10
**S4 Reactivity results**	Catalytic testing data	S11
Figure S4.	CO_2 conversion and product selectivity at 250 °C for Pd/TiO_2 and Pd_xZn_y/TiO_2 catalysts with varying Zn:Pd molar ratios.	S11
Figure S5.	CO_2 conversion and product selectivity at 270 °C for Pd/TiO_2 and Pd_xZn_y/TiO_2 catalysts with varying Zn:Pd molar ratios.	S11
Table S2.	Methane selectivity for all reduced TiO_2-supported catalysts.	S12
Figure S6.	Methanol productivity (mmol h^{-1} kg^{-1}) for reduced Pd/TiO_2 with varying Zn content.	S12
Figure S7.	CO_2 conversion, product selectivity and carbon balance over time for representative Pd_xZn_y/TiO_2 catalysts.	S13
Figure S8.	Powder X-ray diffraction patterns for TiO_2, Pd/TiO_2, and Pd_xZn_y/TiO_2 catalysts with varying Zn:Pd.	S14
Figure S9.	*In situ* XRD showing reflections from grid.	S14
Table S3.	Overlapping reflections at 2θ values for TiO_2 rutile, TiO_2 anatase, PdO, β-PdZn, and ZnO.	S15
**S5 XPS Analysis**	Methodology and further background.	S15
Figure S10.	Pd 3d XPS of calcined, reduced, and post reactor Pd_1Zn_5/TiO_2.	S16
Figure S11.	Pd or PdZn particle size (diameter) determined by TEM and catalyst surface area determined using the BET method by N_2 adsorption.	S16
**S6 Particle diameter distributions**	Electron microscopy measurements.	S17
Table S4.	TEM or HAADF-STEM images and particle diameter distribution histograms	S17
**S7 Computational Results**	Hydrogen adsorption.	S20
Table S5.	E_{ads} of a hydrogen atom on β-PdZn(101) and (110) surfaces	S21
Table S6.	E_{ads} of CO on FCC Pd (111), (100) and (110), and BCT PdZn(101) and (110) across the unique metastable adsorption sites.	S23
**S8 Quantification of Zn in alloy and in ZnO**	Quantification the moles of Zn required to form the alloy and the amount of excess required to form a monolayer coverage across the TiO_2.	S24
Figure S12.	Schematic diagrams of deposition growth modes.	S25
**References**		S26

S1 Reactor and Catalytic Testing.

The reactor and operation. Integrated Workflow manager, based on LabView software, was used to operate the reactor, and was automated using Siemens WinCC software. The high throughput reactor operates all beds under the same reaction conditions and has a fixed bed, continuous flow design. An Equilibar back pressure regulator controlled the pressure in each reactor bed, and gases were fed through a capillary distribution system. To control and measure reactor bed temperature, a thermocouple was positioned inside each of the four heating blocks, containing four reactor beds each. Stainless steel reactor tubes with an internal diameter of 4.0 mm were used. Catalysts were pelleted (0.5 g, 425-600 μm), mixed with F80 silicon carbide (particle size 190 μm) and were supported on a bed of F24 silicon carbide (particle size 750 μm) to limit mass transfer and to ensure the sample was in the centre of the isothermal zone. Prior to testing, the catalysts were reduced *in situ* in a flow of 5% H_2/N_2 gas (400 °C, 1 h, 5 °C/min) and subsequently cooled to 125 °C under N_2. The gas composition was switched to the reactant gas (20% CO_2, 60% H_2, 5 % Ar, 15% N_2), with a flow rate of 30 mL min^{-1} at atmospheric pressure. The system was then pressurised to 20 bar using the gas feed pressure over the Equilibar back pressure regulator and left to stabilise for 4 hours. The reaction temperatures were conducted at 230, 250 and 270 °C. A purge feed of nitrogen equivalent to the reaction feed was used to limit product build-up in the downstream reactor lines, and the downstream oven was set to 120 °C. The products were analysed *via* online gas chromatography (Agilent 7890B system with a flame ionisation detector (FID) and thermal conductivity detector TCD) for which Argon was used as an internal standard. Four injections were taken at each temperature per reactor bed, and a Vici stream selection valve was switched between the reactor beds to allow sampling of the products.

From comparison of the number of moles of CO_2 ($n_{\mathrm{initial\ CO_2}}$) in the calibration at 125 °C against the number of moles of CO_2 at each temperature ($n_{\mathrm{final\ CO_2}}$), CO_2 conversion ($C_{\mathrm{CO_2}}$) was calculated (Equation S1). Methanol, methane, CO, and trace amounts of dimethyl ether (DME) were the products observed. Equation S2 was used to calculate selectivity (S_{x}) for each product, x from the moles of product x, (n_{x}) divided by the total number of moles for all products ($\sum n_{\mathrm{y}}$). Since only C_1 products were observed at quantifiable levels, the carbon balance (CB) was calculated using the

number of moles of initial CO_2 ($n_{\text{initial } CO_2}$) and total moles of products ($\sum n_y$) divided by the number of moles of initial CO_2 (Equation S3).

$$C_{CO_2}(\%) = \frac{n_{\text{initial } CO_2} - n_{\text{final } CO_2}}{n_{\text{initial } CO_2}} \times 100 \qquad \text{Equation S1}$$

$$S_x\ (\%) = \frac{n_x}{\sum n_y} \times 100 \qquad \text{Equation S2}$$

$$CB\ (\%) = \frac{n_{\text{final } CO_2} + \sum n_y}{n_{\text{initial } CO_2}} \times 100 \qquad \text{Equation S3}$$

S2 Characterisation Methodology

XRD. Powder X-ray diffraction (XRD) patterns were measured on two PANalytical X'pert Pro powder diffractometers operating at 40 kV, 40 mA using Cu Kα radiation ($\lambda = 1.54 \times 10^{-10}$ m) with a Ge (111) single crystal monochromator, one for ambient measurements and one for in-situ XRD. The International Centre for Diffraction Data (ICSS) database was used as a reference to assign present phases. *In situ* XRD measurements were ramped from 50 °C - 600 °C, at 40 °C min^{-1}, with measurements conducted at 50 °C intervals, using a (θ-θ) PANalytical X'pert Pro powder diffractometer. A Cu Kα radiation source (40 kV, 40 mA) was fitted with a position sensitive detector and an Anton Parr XRK reaction cell. The gas flow was controlled through a Bronkhorst mass flow controller set to 30 mL min^{-1} (5 % H_2/Ar).

XPS. A Kratos Axis Ultra DLD system was used to collect XPS spectra using monochromatic Al Kα X-ray source operating at 120 W (10 mA x 12 kV). Data was collected with pass energies of 160 eV for survey spectra, and 40 eV for the high-resolution scans with step sizes of 1 eV and 0.1 eV respectively.

The system was operated in the Hybrid mode of operation which uses a combination of magnetic and electrostatic lenses for electron collection over an analysis area of approximately 300×700 μm^2. A magnetically confined charge compensation system was used to minimize charging of the sample surface, and all spectra were taken with a 90° take of angle. A pressure of *ca.* 1×10^{-9} Torr was maintained during collection of the spectra, with base pressure of the system 5×10^{-10} Torr.

Data was quantified using CasaXPS (v2.3.26) using modified Wagner sensitivity factors as supplied by the manufacturer, after subtraction of a Shirley background. Where applicable, peak fitting was achieved using a Voigt functions (LA line shape

option in CasaXPS) and derived from standard bulk materials where possible (*e.g.* Pd foil and PdO). The line shape for PdZn alloy is assumed to be asymmetric in the same way as the metallic Pd.

XAS. *In situ* X-ray Absorption Spectroscopy (XAS) studies were conducted at the Swiss-Norwegian BM31 beamline at the European Synchrotron Radiation Facility.[1] The catalyst was sieved to a particle size within 106-212 μm and placed inside a 1.5 mm quartz capillary microreactor with a wall thickness of 20 μm, fixed between two quartz wool plugs. The capillary was glued with a UV resin onto the metallic frame that fits inside the furnace to allow for homogenous heat distribution in the capillary.[2] A K-type thermocouple was in contact with the furnace to monitor and control the temperature. The gas flow rate was controlled by the beamline's custom gas delivery system, equipped with Bronkhorst mass flow controllers. The catalysts were reduced *in situ* in a flow of 5% H_2/Ar gas at 5°C/min up to 400 °C for 1 h, while the effluent gases of the reactor were analysed by mass spectrometry (Omnistar GSD 300 O, Pfeiffer Vacuum). XAS measurements were collected at the Pd and Zn K-edges in transmission mode in cycles throughout the whole reduction process. Thanks to the small gap between both Si (111) monochromator crystals, both the Pd K-edge and Zn K-edge could be reached with the same indexed crystal, therefore avoiding the need to switch them to reach higher energies and minimising delays when changing edges. Energy calibration was conducted using the Pd and Zn foil standards collected in parallel to the spectra of the sample of interest. XAS data was analysed using Athena programme within the Demeter software package.[3]

Table S1. Properties of TiO_2, Pd/TiO_2 and Pd_xZn_y/TiO_2 including calculated and measured metal loading, particle diameters and surface area values.

Catalyst	Zn:Pd ratio	Pd, Calc / wt.%	[a]Pd, Obs. / wt.%	Zn, Calc. / wt.%	[a]Zn, Obs. / wt.%	[b]Particle dia. / nm	[c]Surf. Area /$m^2 g^{-1}$
**TiO_2**	-	n/a	n/a	n/a	n/a	n/a	55
**Pd/TiO_2**	0	5.0	5.0	n/a	n/a	5.7±1.4	52
**$Pd_1Zn_{0.1}/TiO_2$**	0.1	5.0	5.2	0.3	0.3	6.0±1.6	54
**$Pd_1Zn_{0.5}/TiO_2$**	0.5	5.0	5.3	1.5	1.5	4.3±2.8	52
**Pd_1Zn_1/TiO_2**	1	5.0	5.7	3.1	3.3	7.5±2.3	49
**$Pd_1Zn_{1.5}/TiO_2$**	1.5	5.0	5.8	4.6	4.4	5.9±1.5	48
**Pd_1Zn_2/TiO_2**	2	5.0	5.7	6.1	7.2	7.3±2.8	45
**Pd_1Zn_3/TiO_2**	3	5.0	5.5	9.2	10.1	6.2±1.7	43
**Pd_1Zn_4/TiO_2**	4	5.0	5.5	12.3	13.5	7.1±2.3	45
**Pd_1Zn_5/TiO_2**	5	5.0	5.5	15.4	16.5	8.3±3.0	39
**Pd_1Zn_{10}/TiO_2**	10	5.0	5.1	30.7	31.2	7.7±3.8	37
**Pd_1Zn_{15}/TiO_2**	15	5.0	5.3	46.1	43.7	7.1±2.3	32
**Pd_1Zn_{20}/TiO_2**	20	5.0	5.5	61.4	53.7	6.5±2.3	30

Note: [a]Observed loadings determined by ICP-MS, [b]Particle diameter measured by TEM (100-200 particles) omitting particles > 10 nm which largely consisted of ZnO, [c] Surface Area determined by the BET method by N_2 adsorption.

S3 Computational Methods

The Fritz Haber Institute *ab initio* molecular simulations (FHI-aims) software package was used for full potential all-electron DFT calculations, with the Pythonic Atomic Simulation Environment (ASE) used for management of calculation geometries.[4,5] The default convergence criteria within FHI-aims for self-consistent field (SCF) calculations were used, *i.e.*, the changes between the current and previous SCF iterations in charge density, sum of eigenvalues and total energy were below $N \times 1.67 \times 10^{-5} e\ a_0^{-3}$, 10^{-3} eV and 10^{-6} eV, respectively, where N is the number of atoms in the model. Scalar relativistic treatment of kinetic energy for all elements was achieved by the atomic zero-order regular approximation (ZORA), and a Gaussian-type broadening with width of 0.01 eV was applied to the occupation of electronic states. In the work of Kabalan *et al.*,[6] the accurate semi-local Bayesian error estimation density functional (mBEEF)[7] exchange correlation density functional (DF) as implemented in the LibXC DF library[8] was highlighted as a suitable choice for modelling Cu and Pd surfaces. A

k-grid density of one **k**-point per (0.018 × 2π) $Å^{-1}$ and a default "light" basis set (version: 2020) has been used for geometry optimisations, providing converged structural, energetic and electronic accuracy, with the latter confirmed via Mulliken charge population analysis.[4,6,9] For geometry optimisations, convergence was deemed complete when forces on all unconstrained atoms were less than 0.01 eV $Å^{-1}$. Spin-paired calculations were used for periodic calculations; gas-phase adsorbate structures were calculated both spin-paired and spin-unpaired, and the energy of the more stable system was considered for reference to periodic calculations.

Low-index facets of metal and alloy surfaces are often chosen to study the chemical phenomena occurring at the gas-solid interface between the nanoparticles of the catalyst and reactants in the gas-phase. Monometallic surfaces were constructed from optimised face-centred cubic (FCC) Pd and hexagonal close-packed (HCP) Zn bulk structures, based on the outcomes of the work by Kabalan *et al.*[6] A one-sided surface model was created for Pd(111), Pd(110), Pd(110), and Zn(0001) surfaces using supercells with cell dimensions of (3 x 3 x 7), and a vacuum layer of 40 Å added in the direction perpendicular to the surface plane (*i.e.*, *z*). The bottom three atomic layers were constrained to their bulk positions and the 4 top surface layers were unconstrained; and, due to the one-sided nature of the slab models considered, a dipole-correction was used in all calculations. For the PdZn alloy surface, the body-centred tetragonal (BCT) PdZn bulk cell was used to create PdZn(101) and PdZn(110) surfaces in a similar manner to the monometallic surfaces; however, a (2 x 3 x 7) supercell was used, as the BCT primitive cell contains two atoms. The calculated surface energies of low-index PdZn surfaces follow the trend: (101) < (110) < (111), whereas Pd surface energies are in the order: (111) < (100) < (110). The bimetallic alloy ordering was obtained from the experimental studies of Peterson *et al.*[10] and Nowicka *et al.*[11] The lower the surface energy, the less chemically labile the surface and the higher the expected representation as a facet on the nanoparticle.[10,11] For ZnO, an optimised wurtzite cell was used to create $(10\bar{1}0)$ and $(11\bar{2}0)$ surfaces, with supercell dimensions of (4 x 2 x 10) and (2 x 2 x 6) respectively.

For catalytic surface reactions, the adsorption energy (E_{ads}) in Equation S4 measures the interaction between a surface and an adsorbate, and is deduced from the

comparison of energies of optimised gas-phase adsorbate (E_{gas}), pristine surface ($E_{pristine}$) and the optimised combined system (E_{slab}).

$$E_{ads} = E_{slab} - (E_{pristine} + E_{gas}) \qquad \text{Equation S4}$$

The definition used here is such that negative E_{ads} indicates exothermic interactions. For the case of H atom adsorption E_{gas}(H) is taken to be ½ E_{gas}(H_2). For Pd and PdZn surfaces, the adsorbates are placed at 1 and 2 Å above the surface for hydrogen atom and other species, respectively, at adsorption sites inequivalent by symmetry. The adsorption sites considered for Pd facets are shown in Figure S1 and for PdZn surfaces in Figure S2.

The location of the surface sites and placement of defects on the ZnO surfaces are shown in Figure S3. A ball and stick visualisation of slabs used for simulation of ZnO surfaces with vacancy sites considered in the study marked. a) ZnO($1010\_$): $A - lower\ surface\ oxygen\ vacancy, B - upper\ surface\ oxygen\ vacancy.$ b) ZnO($\_$ 1120): $A - surface\ oxygen\ vacancy. Grey\ and\ red\ spheres\ represent\ Zn\ and\ O\ atoms,$ $respectively; in\ each\ case\ the\ view\ is$ *down* onto the surface ab-plane and black lines represent unit cell boundaries of the simulation cell. Atomic radii are Shannon crystal radii for tetracoordinate Zn2^{+} and O2- . On the stoichiometric surfaces CO_2 was initially configured to form a carbonate by placing the carbon atom at a suitable distance from a surface oxygen anion and changing the O-C-O angle so that the three O atoms co-ordinated to C formed a rough equilateral triangle with the C atom at the centre.

For ZnO$(11\bar{2}0)$ all surface oxygen anions are symmetry equivalent and so there is only one choice for the creation of a surface oxygen vacancy. Whereas for ZnO$(10\bar{1}0)$ there is less than 1 Å in the z-direction between the top two oxygen anion layers. So, it is feasible that oxygen could be abstracted from either the lower surface layer (defect site 1), or from the upper surface layer (defect site 2) and both have been considered here. For the defective surfaces the adsorbed CO_2 molecule was arranged in a similar way to that described for the stoichiometric surfaces but with the additional requirement that one of the O atoms originating from the molecule was placed near to the surface vacancy.

For transition state calculations, a machine learning nudged elastic band (MLNEB) method was used to identify saddle points and minimum energy paths (MEPs).[12,13] A spring constant of 0.05 eV Å^{-1} was deemed sufficient for well-converged MEPs in our previous work and has been used throughout; the convergence criterion of forces on all unconstrained atoms of below 0.05 eV Å^{-1}, with energy uncertainty below 0.03 eV.[14] The activation energy of a reaction step, E_{act}(species), is defined as the difference between the total energy of the transition state at the surface and the total energy of the initial geometry of the species at the surface. $E_{act}(species)_d$ and $E_{act}(species)_h$ denote dissociation and hydrogenation processes, respectively.

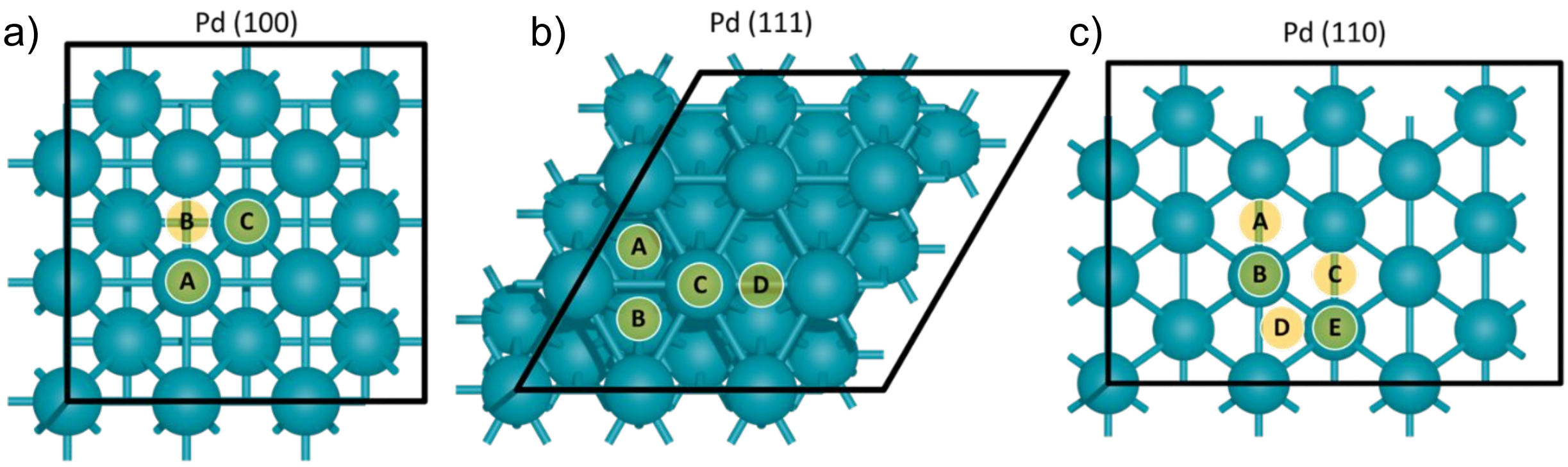

Figure S1. A ball and stick visualisation of face-centred cubic Pd surfaces showing adsorption sites considered in the study. a) Pd(100): A - atop, B – bridge, C – hollow. b) Pd (111): A – FCC, B – HCP, C – atop, D – bridge. c) Pd (110): A – short-bridge, B – atop, C – long-bridge, D – “FCC”, E – hollow. In all cases, the view is down onto the surface *ab*-plane and black lines represent unit cell boundaries of the simulation cell, Pd atoms are coloured blue.

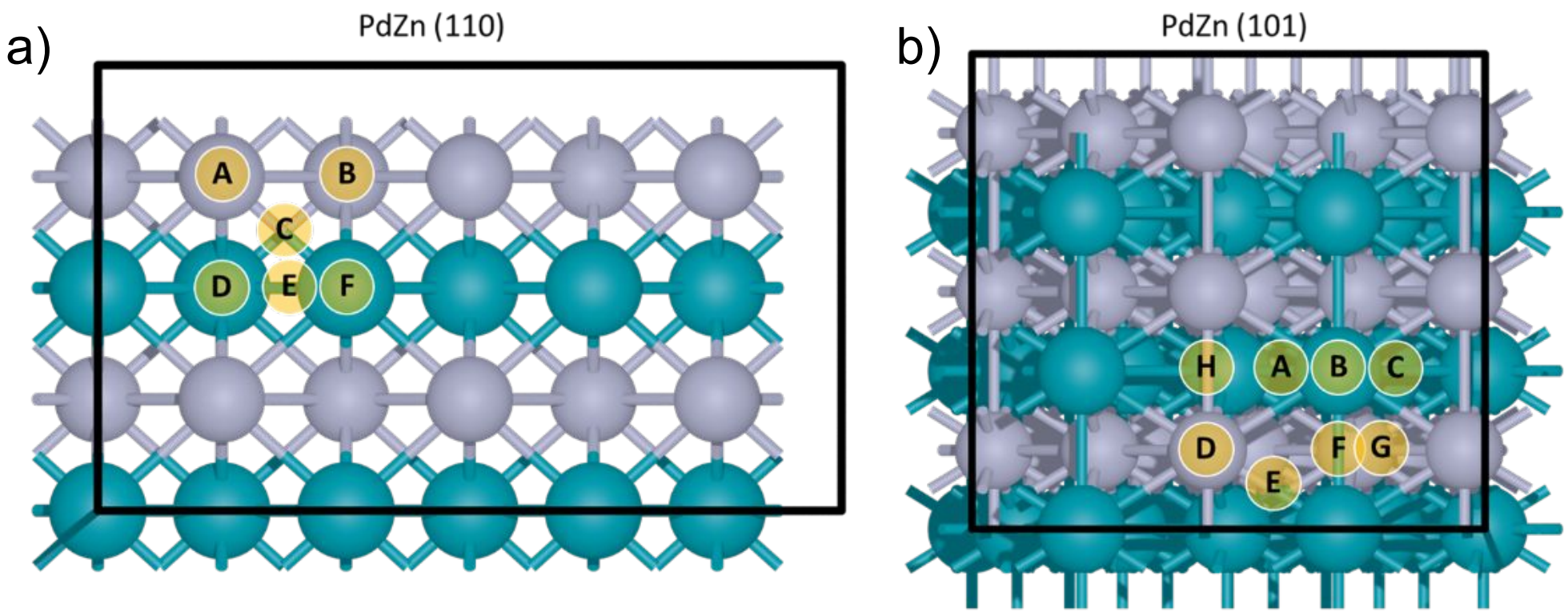

Figure S2. A ball and stick visualisation of body-centred tetragonal β-PdZn surfaces showing adsorption sites considered in the study. a) PdZn (110): A – Zn atop, B – Pd_2Zn_3 hollow, C – Pd-Zn bridge, D - Pd_3Zn_2 hollow, E – Pd off-top, F – Pd atop. b) PdZn (101): A – Pd off-top 1, B – Pd atop, C – Pd off-top 2, D – Zn atop, E – Pd-Zn bridge, F – Pd-Pd bridge, G – Pd_2Zn hollow, H – Zn-Zn bridge. In all cases, the view is down onto the surface *ab*-plane and black lines represent unit cell boundaries of the simulation cell, Zn and Pd atoms are coloured grey and blue, respectively.

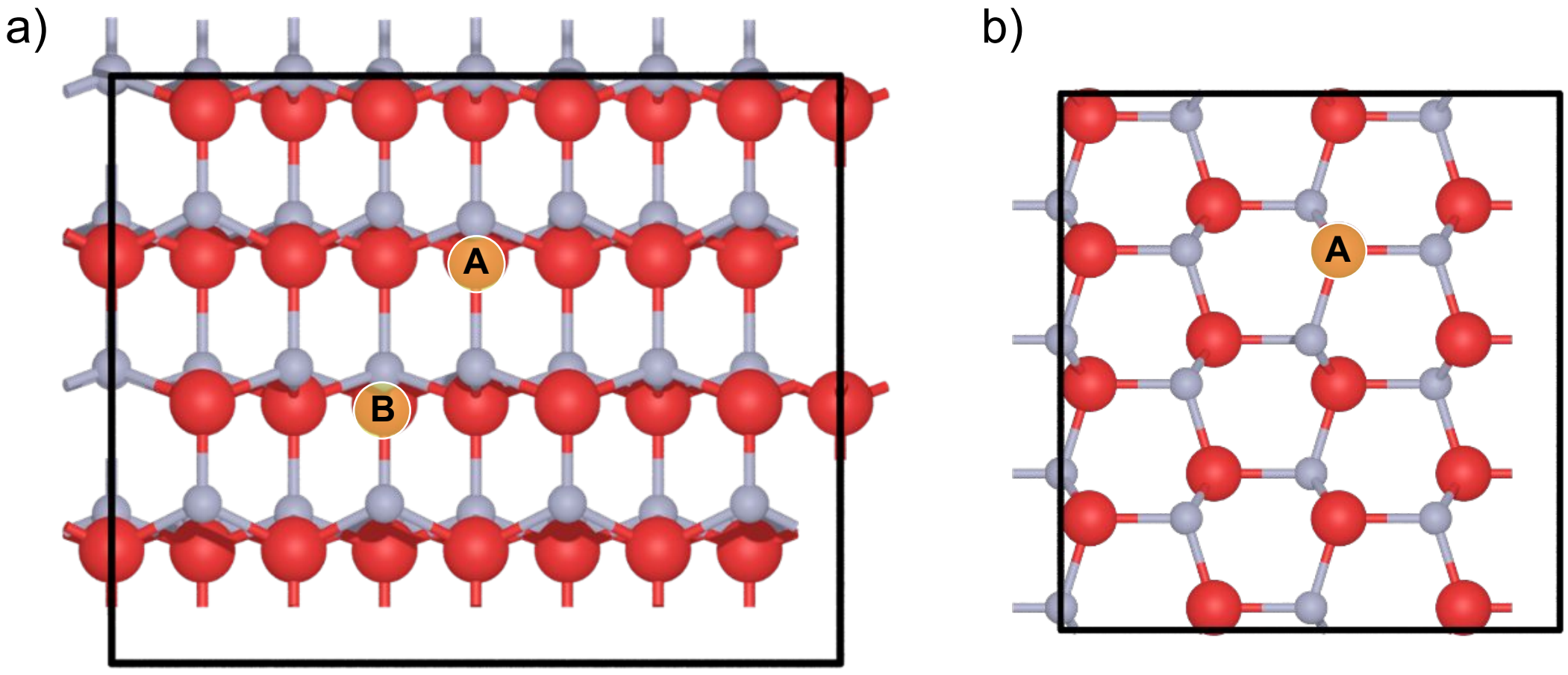

Figure S3. A ball and stick visualisation of slabs used for simulation of ZnO surfaces with vacancy sites considered in the study marked. a) ZnO($\mathbf{10\bar{1}0}$): A – lower surface oxygen vacancy, B – upper surface oxygen vacancy. b) ZnO($\mathbf{11\bar{2}0}$): A – surface oxygen vacancy. Grey and red spheres represent Zn and O atoms, respectively; in each case the view is down onto the surface *ab*-plane and black lines represent unit cell boundaries of the simulation cell. Atomic radii are Shannon crystal radii for tetracoordinate Zn^{2+} and O^{2-}.

S4 Reactivity results

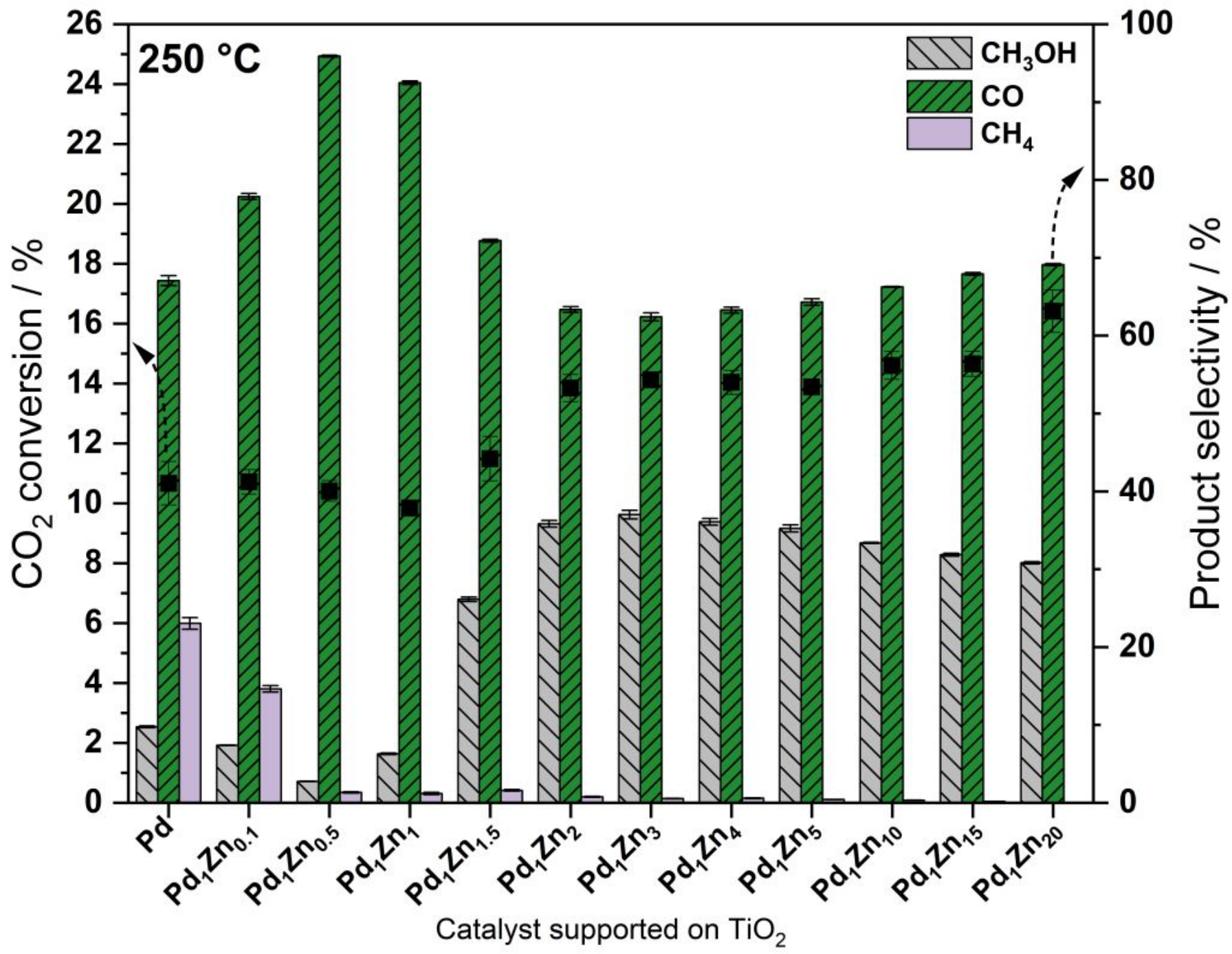

Figure S4. CO_2 conversion and product selectivity at 250 °C for Pd/TiO_2 and Pd_xZn_y/TiO_2 catalysts with varying Zn:Pd molar ratios. ■ represents CO_2 conversion (left y-axis), histogram bars represent selectivity (right y-axis) for the products indicated in key.

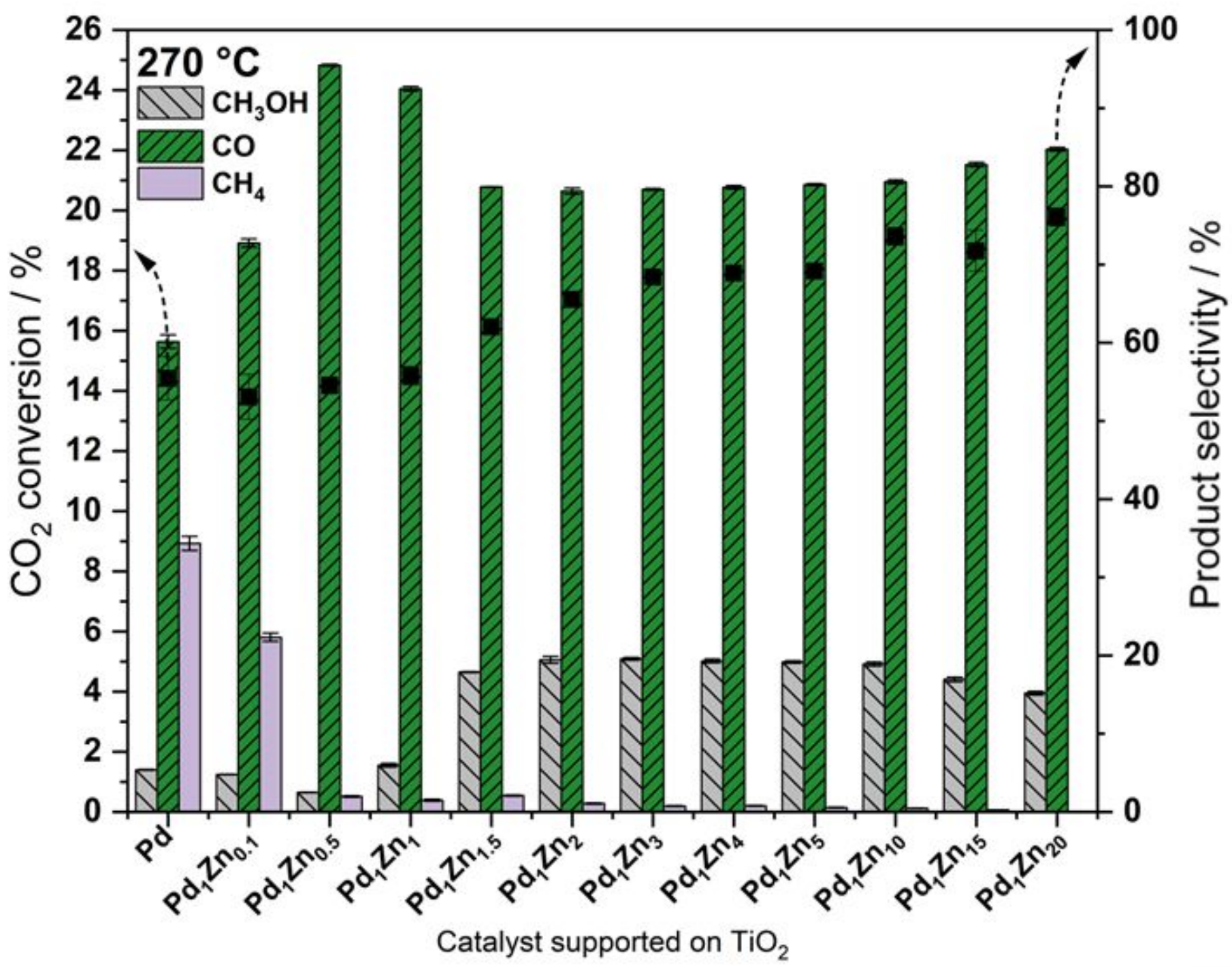

Figure S5. CO_2 conversion and product selectivity at 270 °C for Pd/TiO_2 and Pd_xZn_y/TiO_2 catalysts with varying Zn:Pd molar ratios. ■ represents CO_2 conversion (left y-axis), histogram bars represent selectivity (right y-axis) for the products indicated in key.

Table S2. Methane selectivity for all reduced TiO_2-supported catalysts under reaction conditions (230, 250 and 270 °C).

Catalyst	Zn:Pd ratio	Methane Selectivity / %		
		Reaction at 230 °C	Reaction at 250 °C	Reaction at 270 °C
Pd/TiO_2	0	16.7	23.0	34.3
$Pd_1Zn_{0.1}/TiO_2$	0.1	10.2	14.6	22.3
$Pd_1Zn_{0.5}/TiO_2$	0.5	1.1	1.3	2.0
Pd_1Zn_1/TiO_2	1	1.1	1.2	1.5
$Pd_1Zn_{1.5}/TiO_2$	1.5	1.3	1.6	2.1
Pd_1Zn_2/TiO_2	2	0.6	0.8	1.1
Pd_1Zn_3/TiO_2	3	0.4	0.5	0.7
Pd_1Zn_4/TiO_2	4	0.5	0.6	0.8
Pd_1Zn_5/TiO_2	5	0.3	0.4	0.6
Pd_1Zn_{10}/TiO_2	10	0.3	0.3	0.5
Pd_1Zn_{15}/TiO_2	15	0.1	0.2	0.2
Pd_1Zn_{20}/TiO_2	20	0.0	0.0	0.1

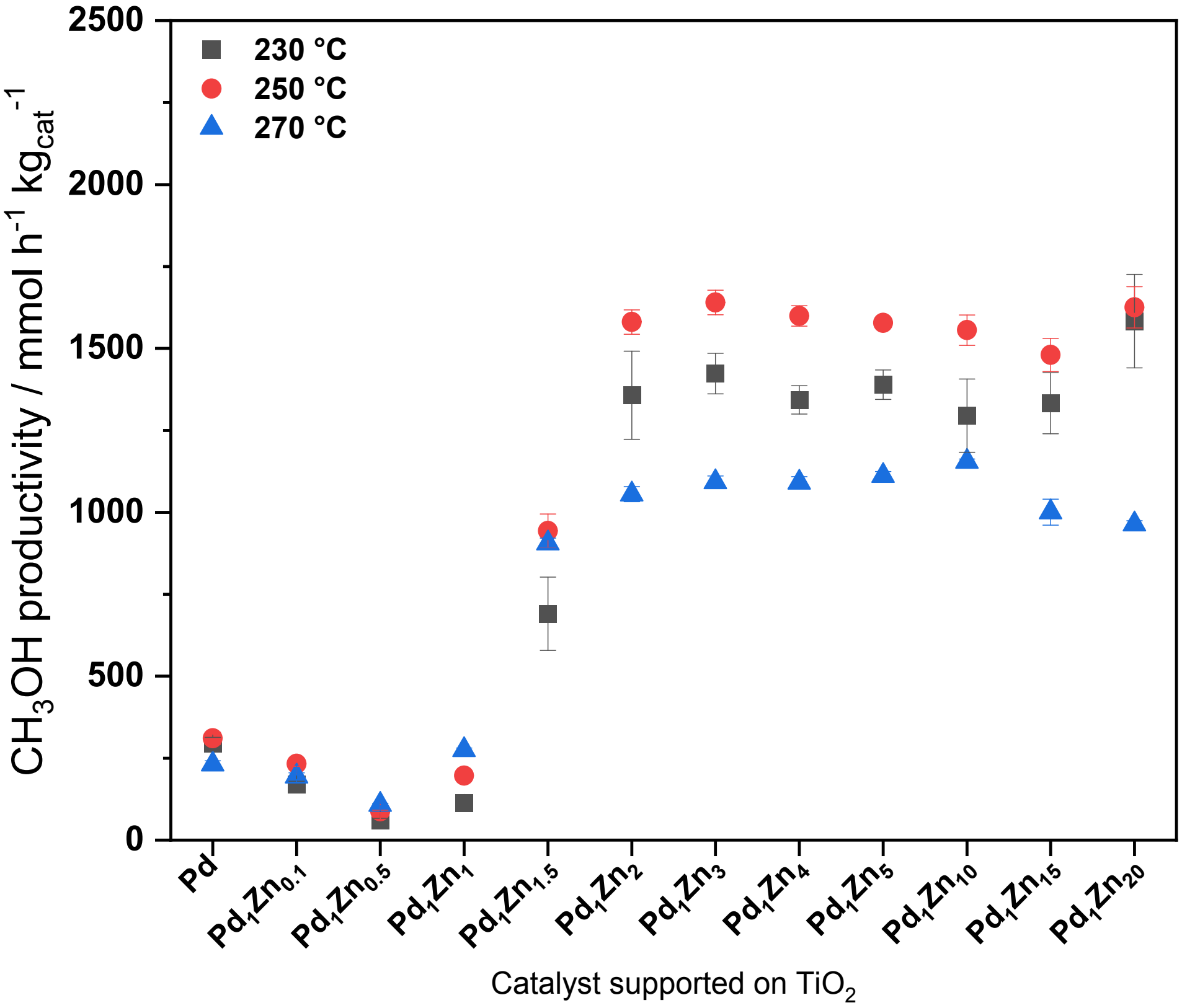

Figure S6. Methanol productivity (mmol h^{-1} kg^{-1}) for reduced Pd/TiO_2 with varying Zn (Pd_xZn_y/TiO_2) added during preparation. Catalysts were pre-reduced *in situ* (400 °C) and tested for CO_2 hydrogenation at 230, 250, and 270 °C (square, circle, triangle, respectively).

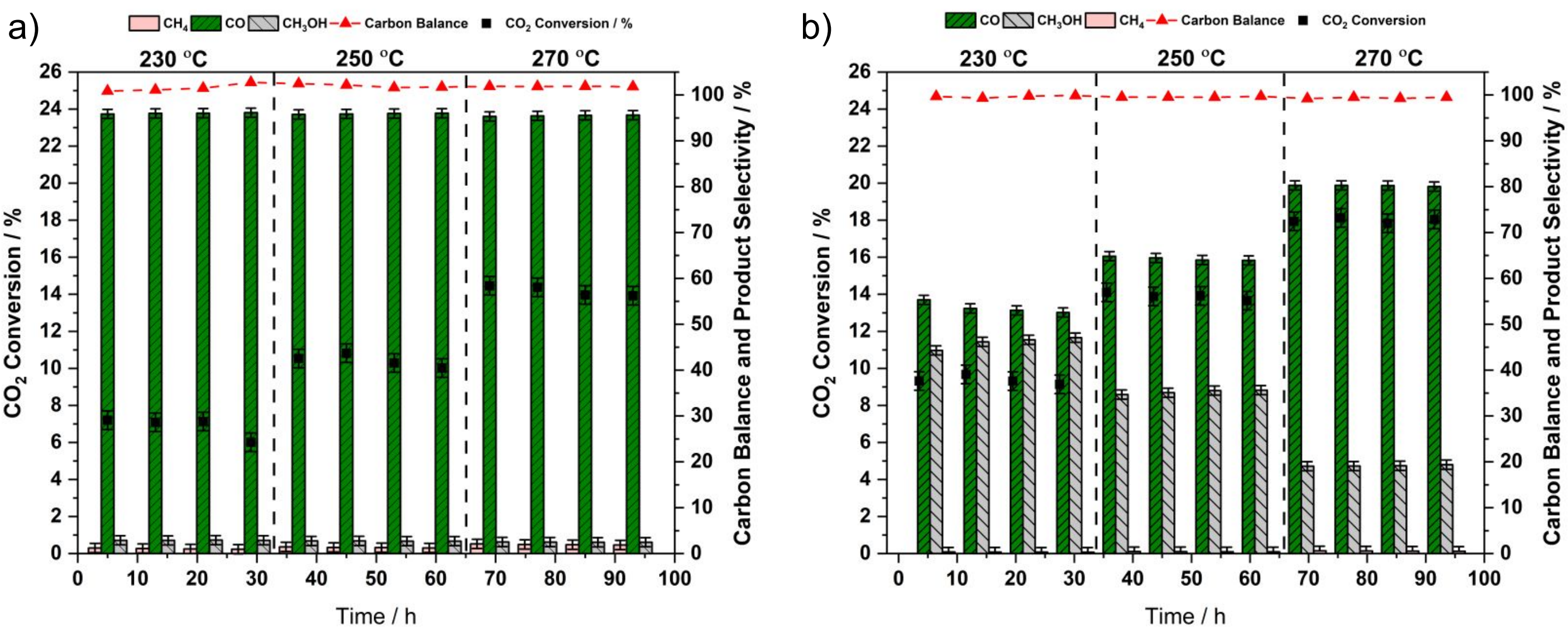

Figure S7. CO_2 conversion, product selectivity and carbon balance over time for a) $Pd_1Zn_{0.5}/TiO_2$ and b) Pd_1Zn_5/TiO_2 catalysts. Catalysts were pre-reduced *in situ* (400 °C) and tested for CO_2 hydrogenation at 230, 250, and 270 °C (square, circle, triangle, respectively). ■ represents CO_2 conversion (left y-axis), histogram bars represent selectivity (right y-axis) for the products indicated in key.

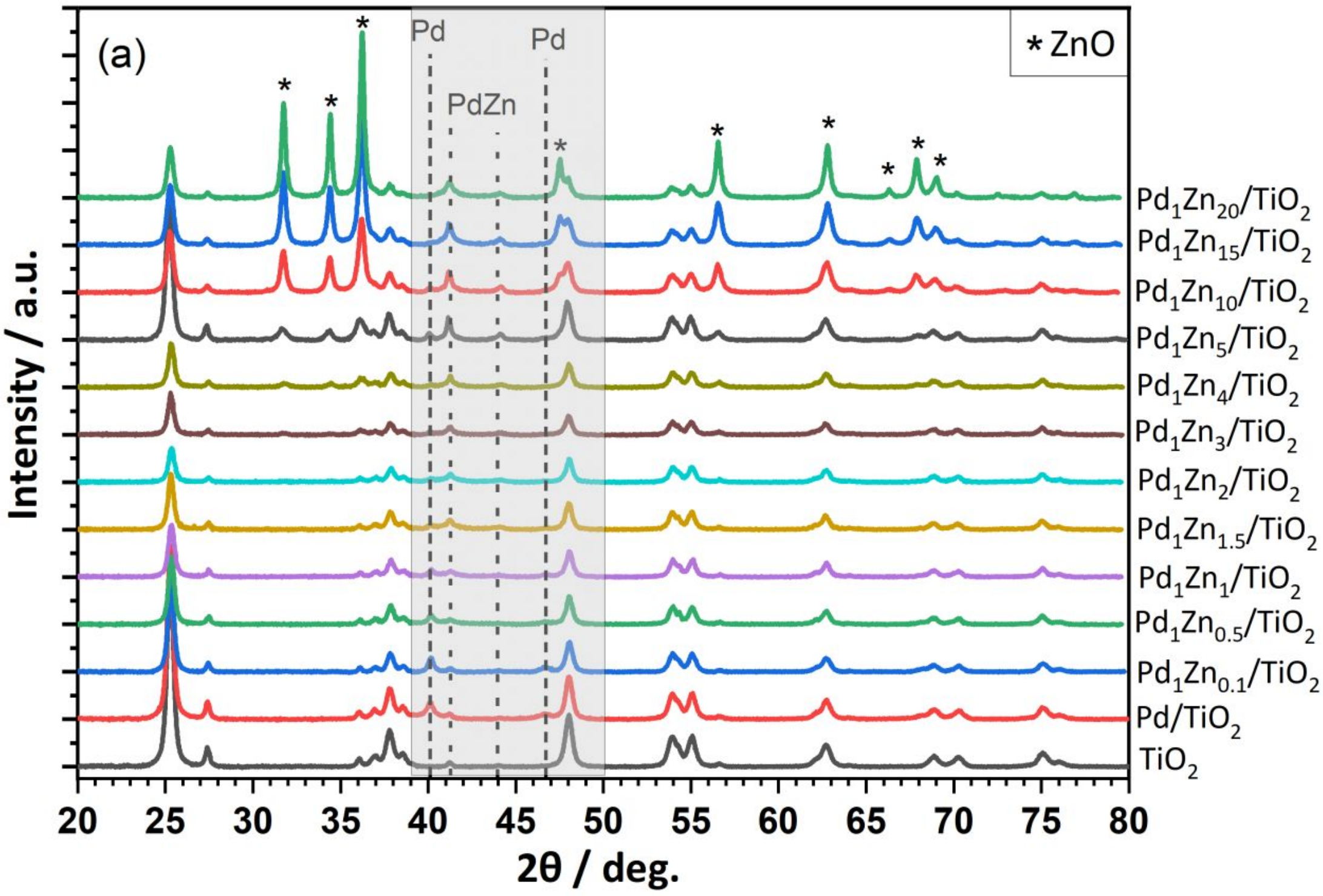

Figure S8. Powder X-ray diffraction patterns for TiO_2, Pd/TiO_2, and Pd_xZn_y/TiO_2 catalysts with varying Zn:Pd ratios after calcination at 500 °C for 16 h, followed by H_2 reduction at 400 °C.

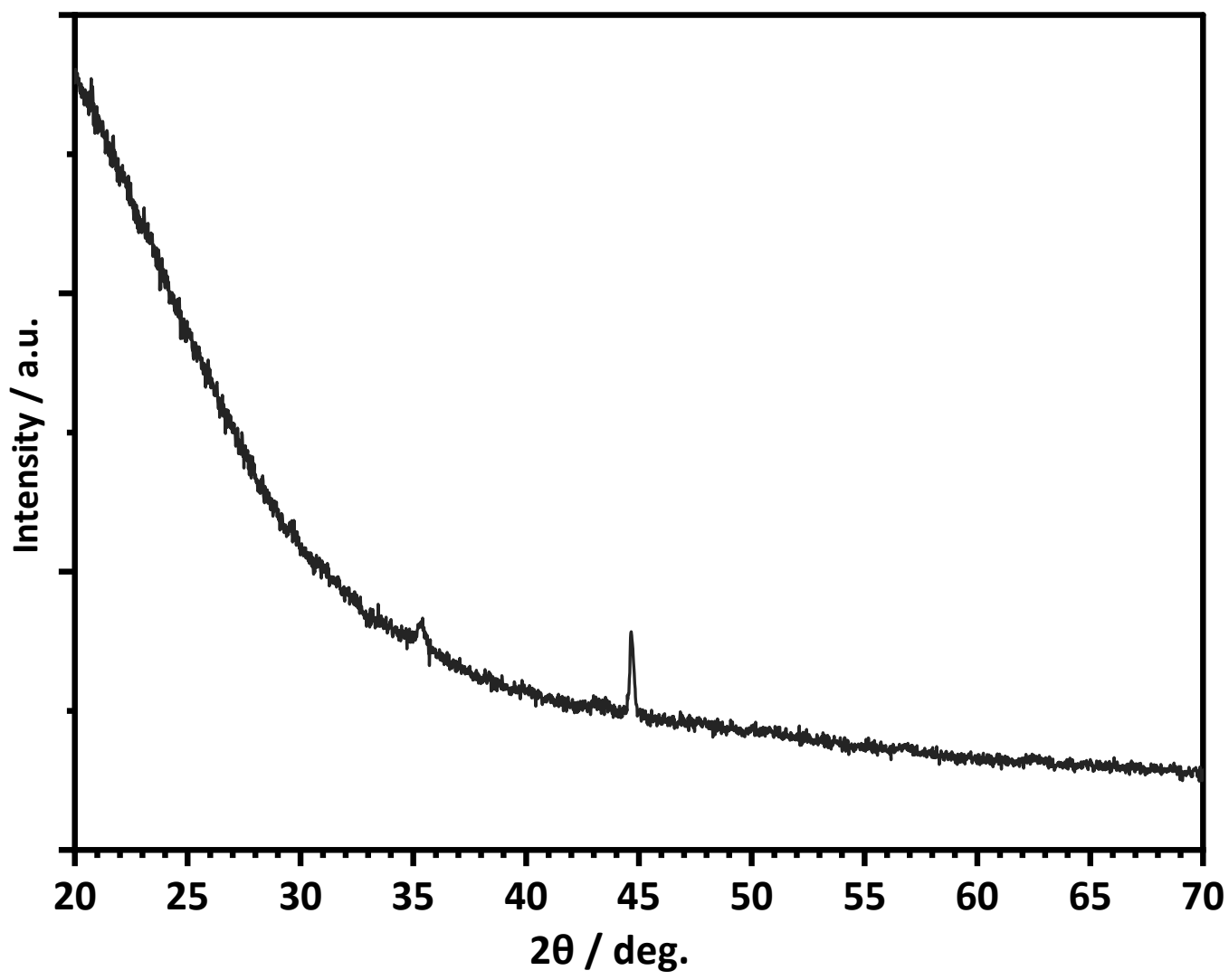

Figure S9. *In situ* XRD showing reflections from grid.

**Table S3.** Overlapping reflections at 2θ values for TiO_2 rutile, TiO_2 anatase, PdO, β-PdZn, and ZnO. All values in degrees.					
TiO_2 rutile (JCPDS 00-004-0551)	TiO_2 anatase (JCPDS 00-004-0477)	PdO (JCPDS 00-006-0515)	Pd^0 (JCPDS 00-005-0681)	PdZn (JCPDS 01-072-2936)	ZnO (JCPDS 00-005-0664)
36.0	36.8				36.3
39.2			40.1		
41.2		41.9		41.1	
44.1				44.1	

S5 XPS Analysis.

Pd 3d XPS spectra for Pd_1Zn_5/TiO_2 and Pd_1Zn_{15}/TiO_2 catalysts comparing the calcined, reduced, and post-reaction samples are presented in Figure S9. Comparing spectra after calcination (Figure 9a and d) with those for the same sample post reduction (Figure 9b and e) shows that a shift in binding energy after reduction occurs. This indicates changes in Pd localised charge due to the reduction of PdO to Pd metal and Pd alloying with Zn. The difference seen between Pd and PdZn alloy binding energy appears counter intuitive as Pd has the higher electronegativity. Indeed, DFT calculations for a PdZn alloy shows that overall electron density moves from Zn to Pd on alloy formation resulting in $Pd^{\delta-}$ $Zn^{\delta+}$.[6] Even so, the 4d-band in the alloy is moved to lower energy than in pure Pd and charge also moves to more diffuse 5s and 5p states, resulting in an overall decrease in electron repulsion and a correspondingly higher 3d binding energy.[15–17] A small peak remains at 336.3 eV, corresponding to surface PdO that arises from exposure to air before admittance to the XPS instrument. The presence of Pd metal might arise through surface restructuring whereby the soft X-rays damage the PdZn alloy, as seen previously by Rightor *et al.*[18] Samples were retrieved after catalytic testing (CO_2 hydrogenation at 230, 250, and 270 °C, 20 bar) and XPS characterisation are shown in Figure S9c and f, corresponding to Pd_1Zn_5/TiO_2 and Pd_1Zn_{15}/TiO_2. Interestingly, the ratio between Pd metal and Pd alloy after reduction and post-reaction remain similar for Pd_1Zn_5/TiO_2 but a slight increase in Pd alloy is observed for Pd_1Zn_{15}/TiO_2, suggesting that the PdZn alloy is favourably formed in CO_2 hydrogenation testing conditions.

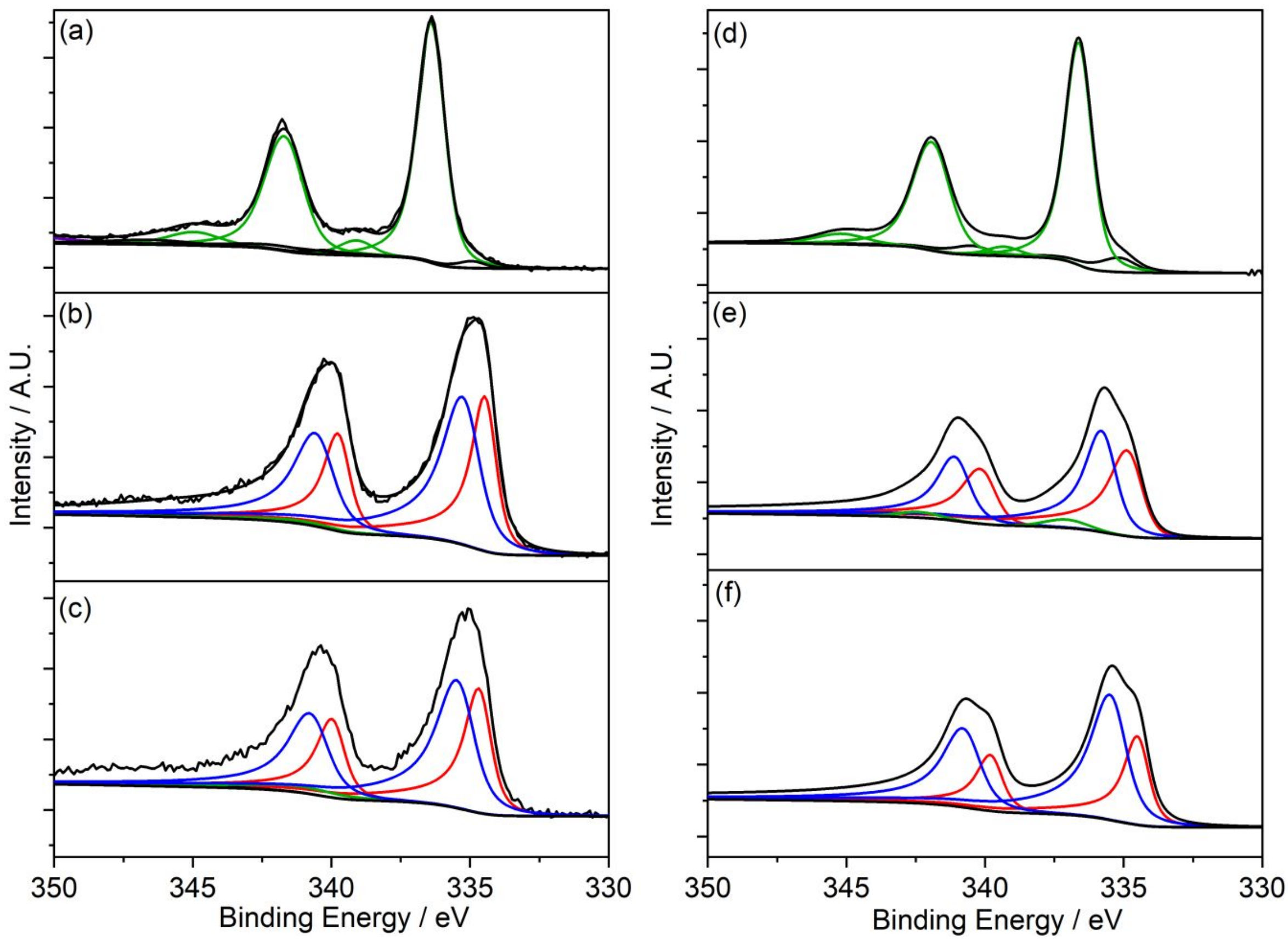

Figure S10. Pd 3d XPS of calcined, reduced, and post reactor Pd_1Zn_5/TiO_2 (a-c, respectively), and calcined, reduced, and post reactor Pd_1Zn_{15}/TiO_2 (d-f), respectively. Pd metal, alloyed Pd, and PdO in the peak fitting are represented in red (334.4 eV) and blue (335.4 eV), green (336.3 eV).

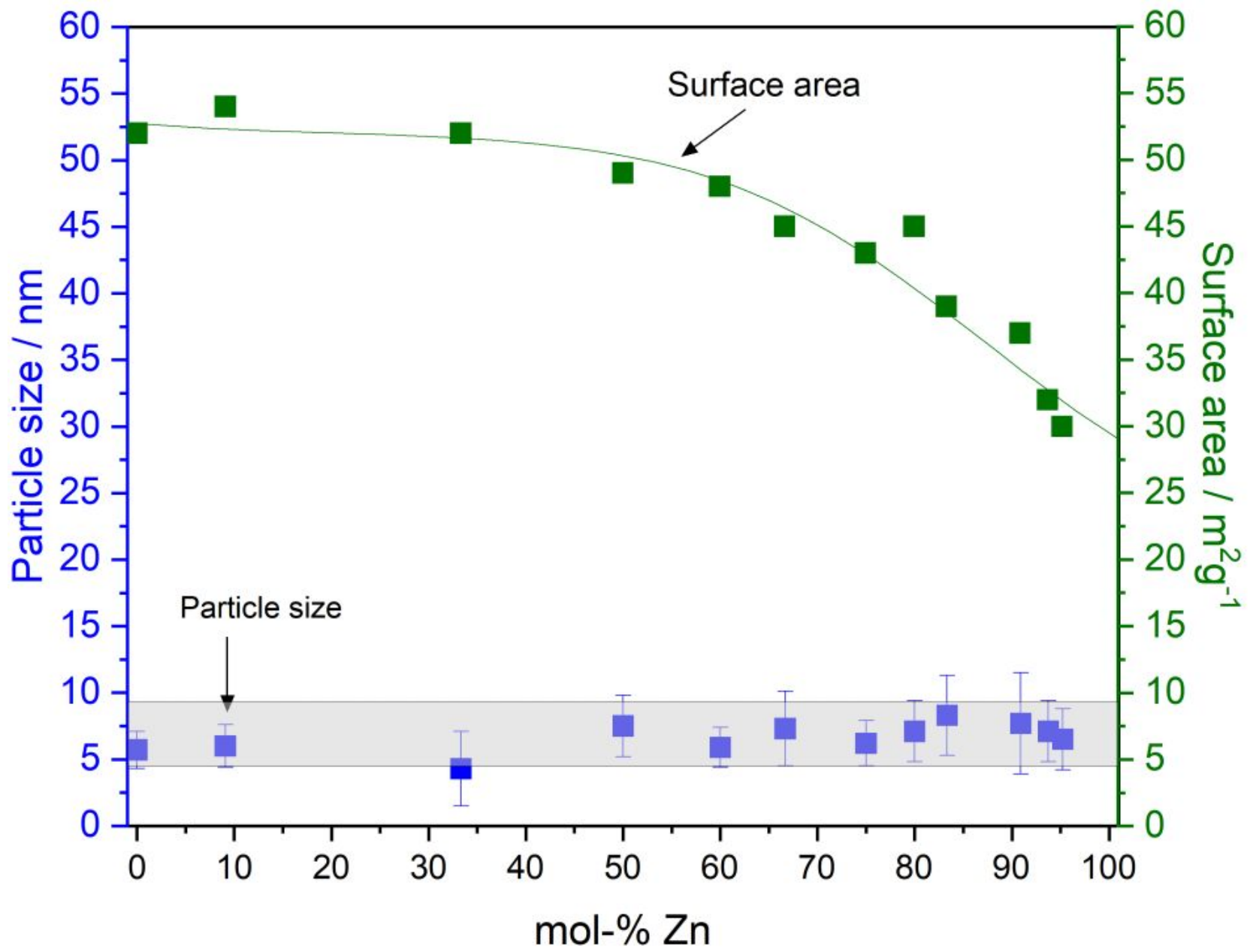

Figure S11. Pd or PdZn particle size (diameter) determined by TEM and catalyst surface area determined using the BET method by N_2 adsorption. Both measurements are plotted as a function of the moles of Zn in the total metal loading in Pd_xZn_y/TiO_2.

S6 Particle diameter distributions

Transmission electron microscopy (TEM) and high-angle annular dark field scanning transmission electron microscopy (HAADF-STEM) were used to determine Pd and PdZn particle size distributions for prepared catalysts by CVI after calcination (500 °C, 16 h) and reduction (400 °C, 5% H_2/Ar, 1 h). 100-200 particles were measured to obtain a representative particle population for each sample and then normalised to relative frequency and cumulative frequency.

Table S4. TEM or HAADF-STEM images and particle diameter distribution histograms for Pd/TiO_2 and Pd_xZn_y/TiO_2. (Zn:Pd 0-2[19])

Zn:Pd ratio	TEM / HAADF-STEM image	Particle diameter distribution histogram	Ref
0	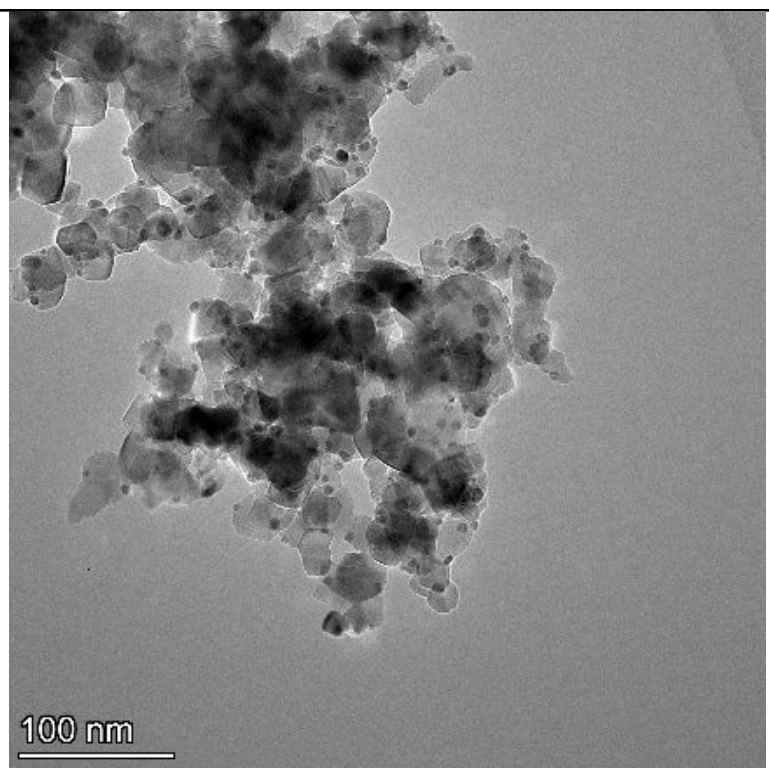	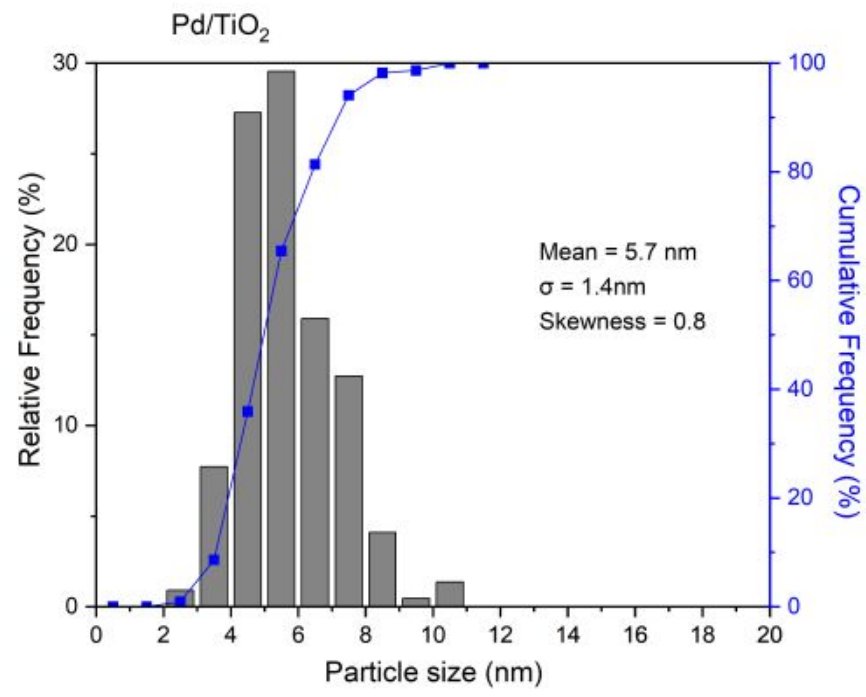	19
0.1	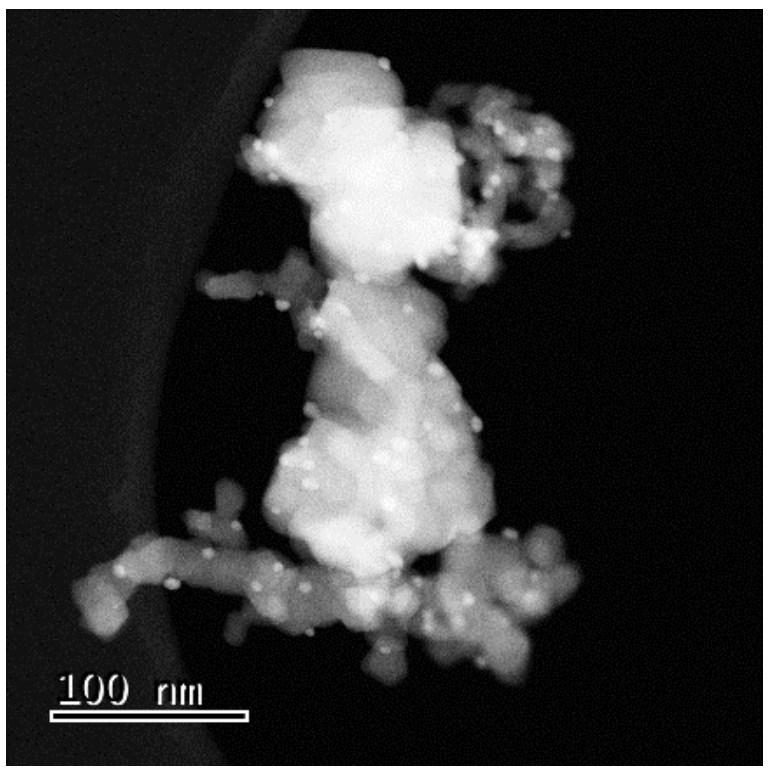	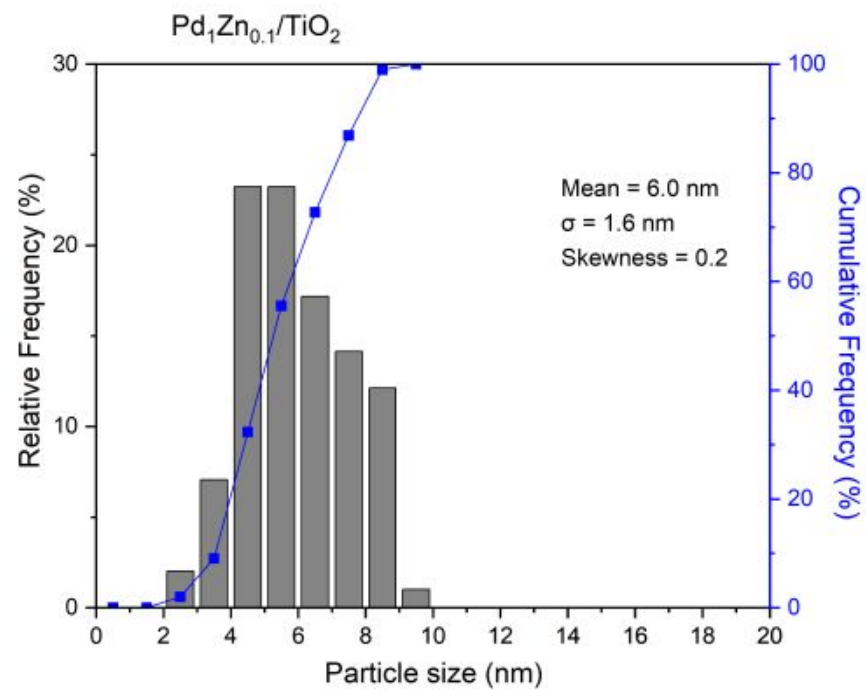	19
0.5	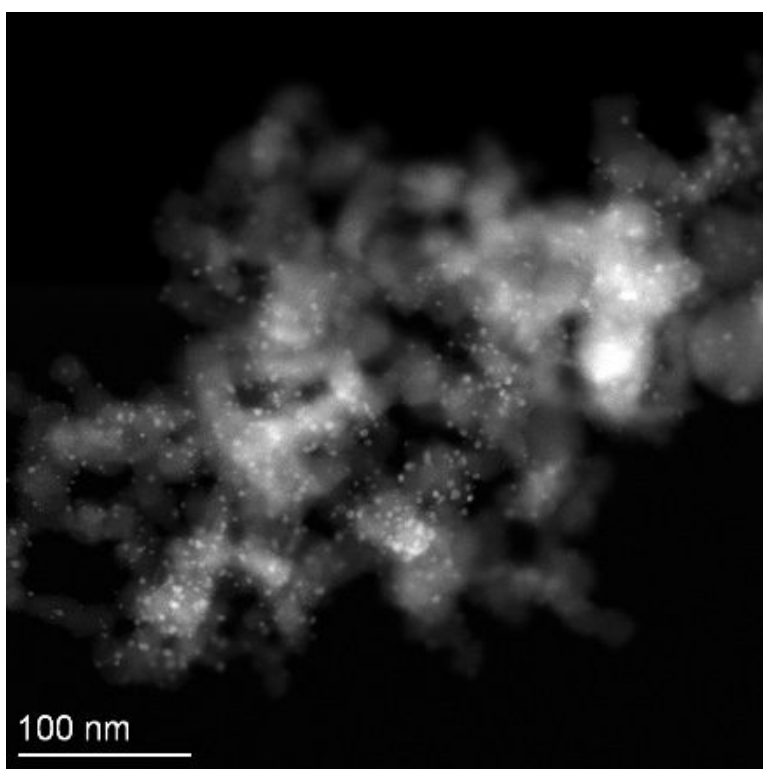	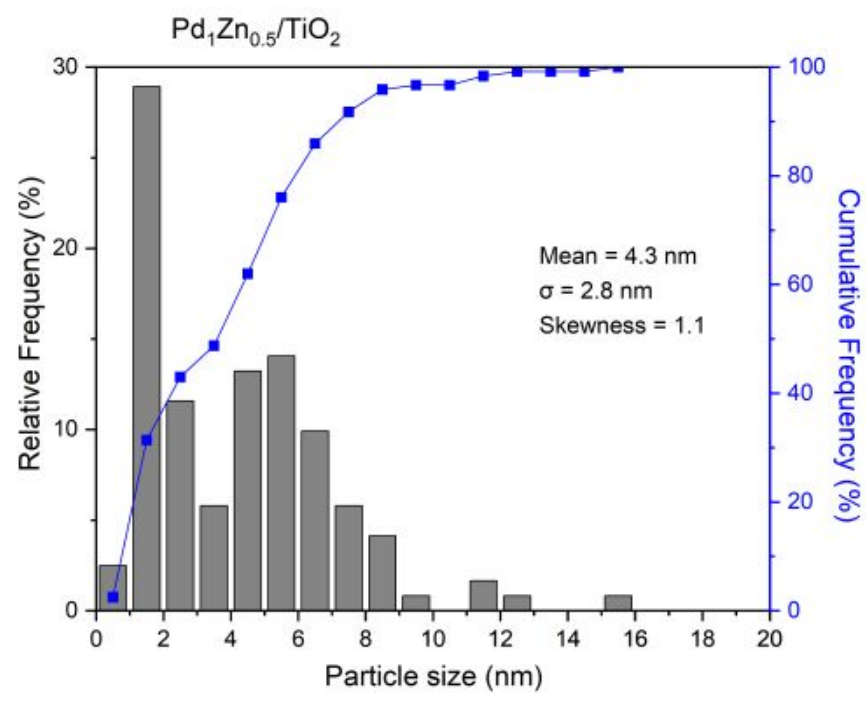	19

1

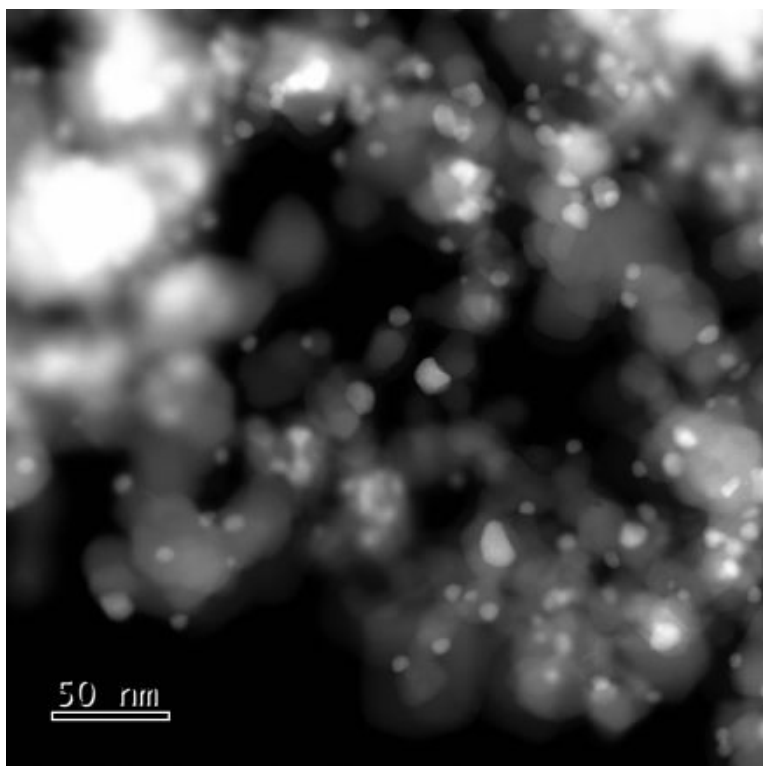

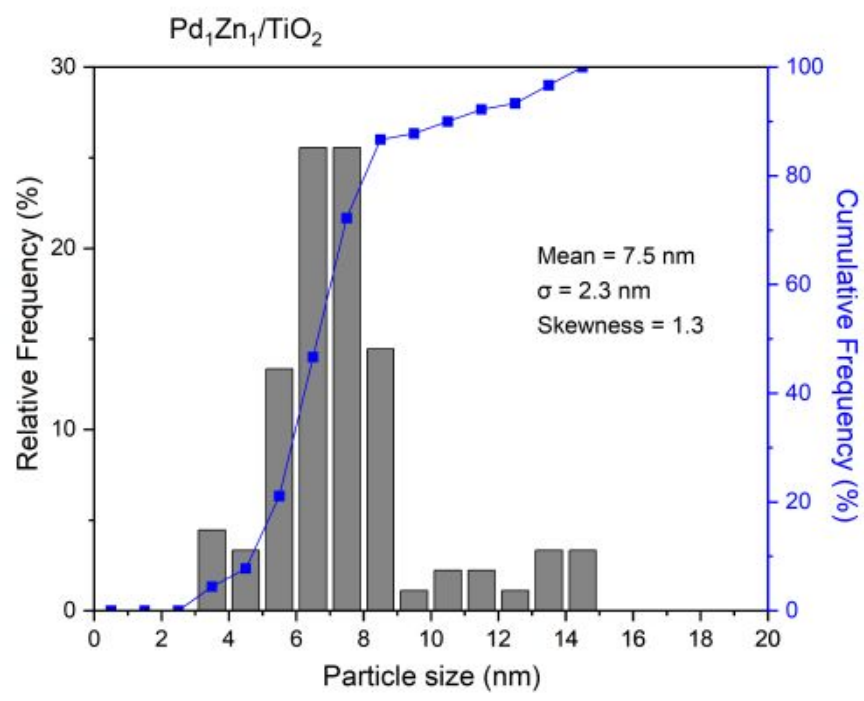

19

1.5

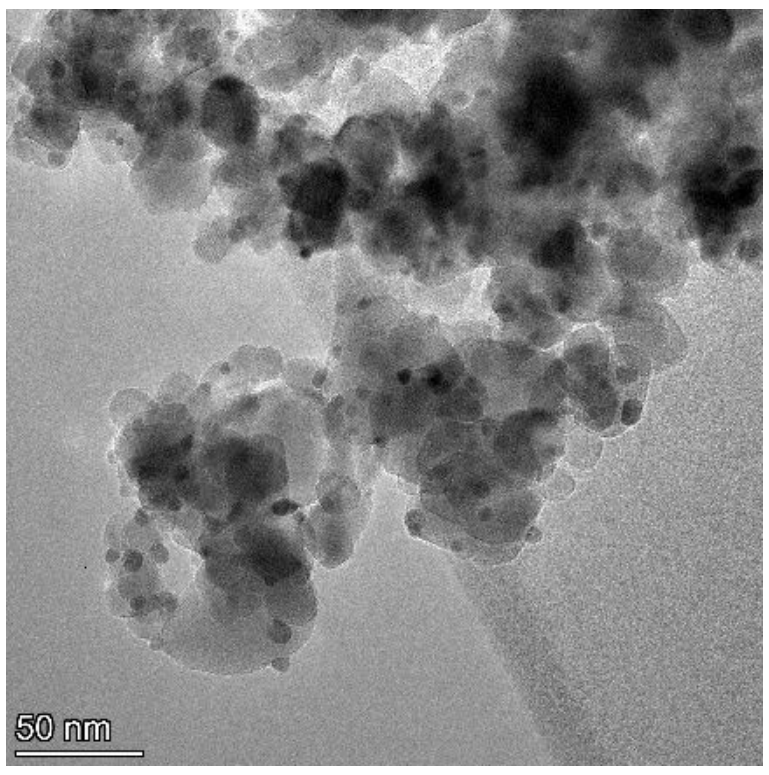

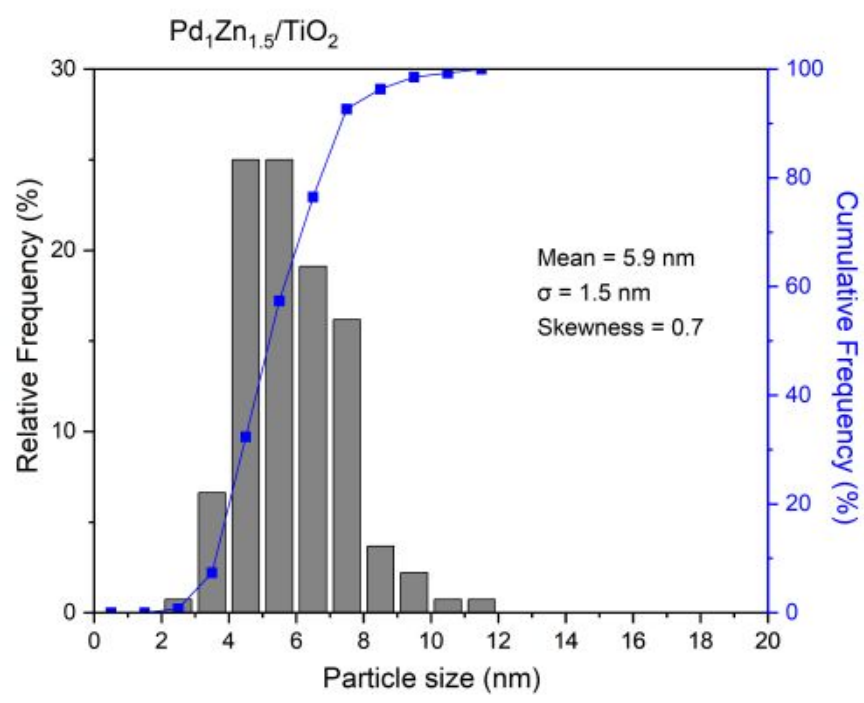

19

2

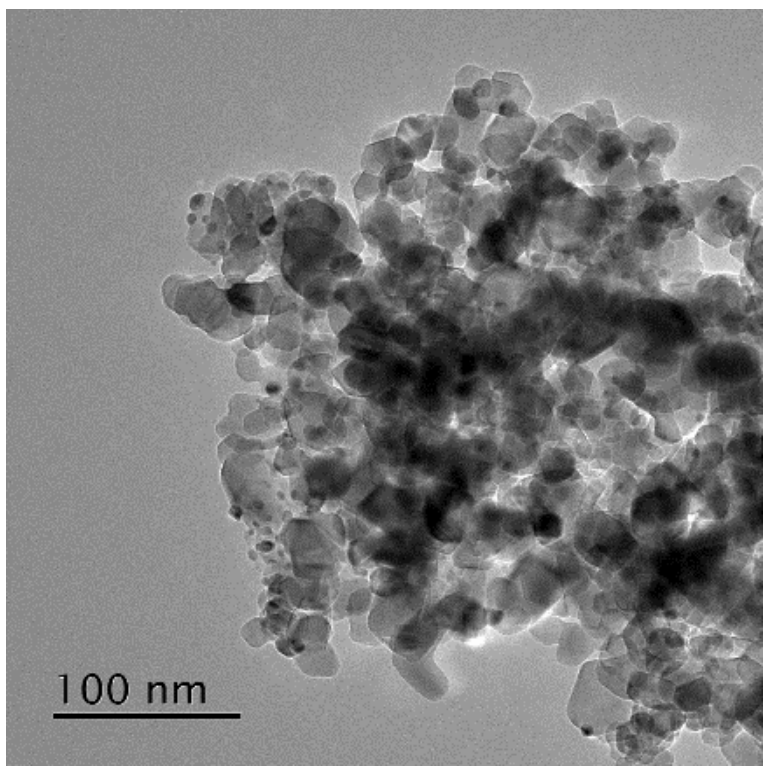

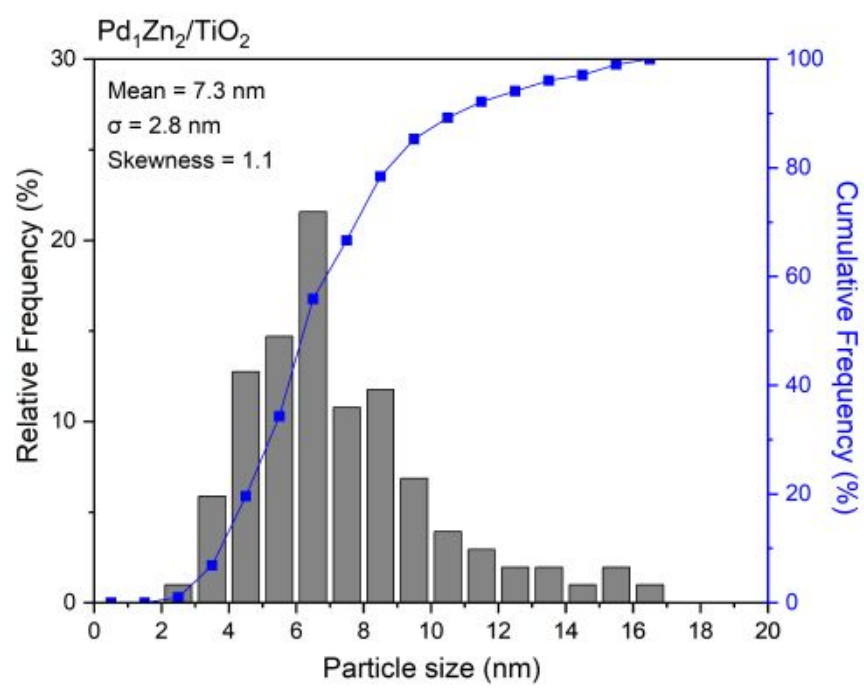

19

3

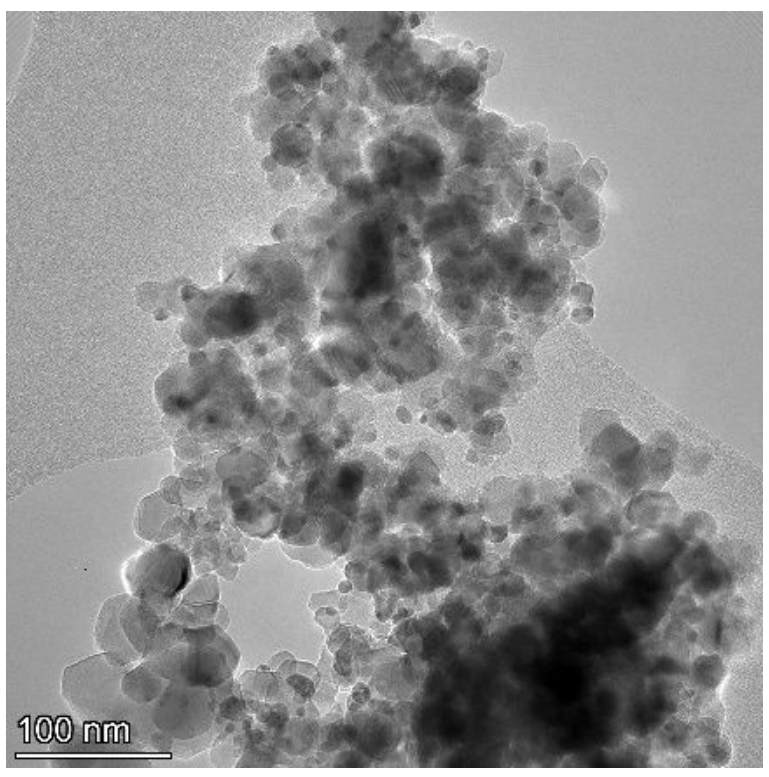

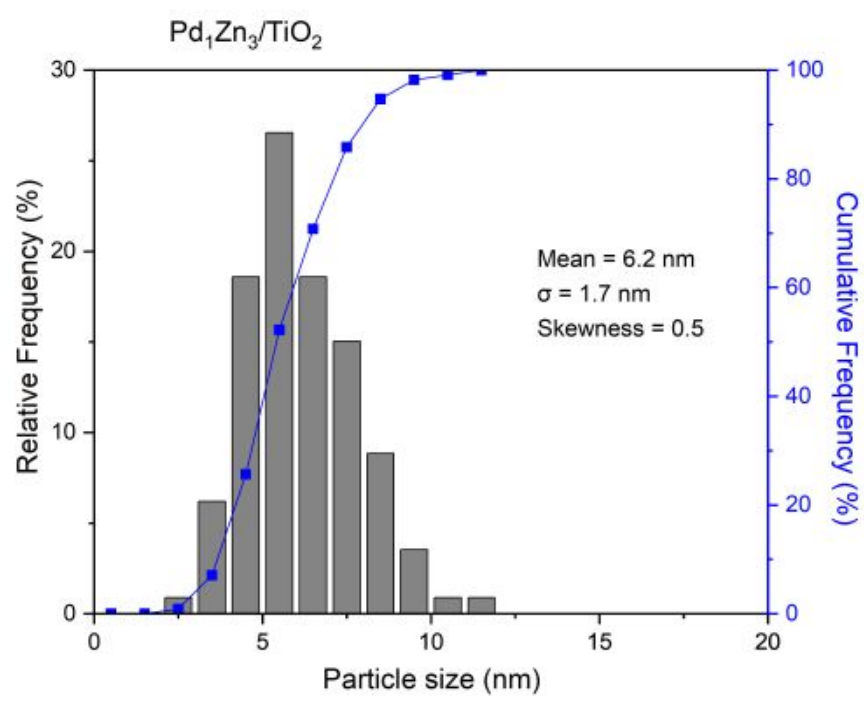

This work

4

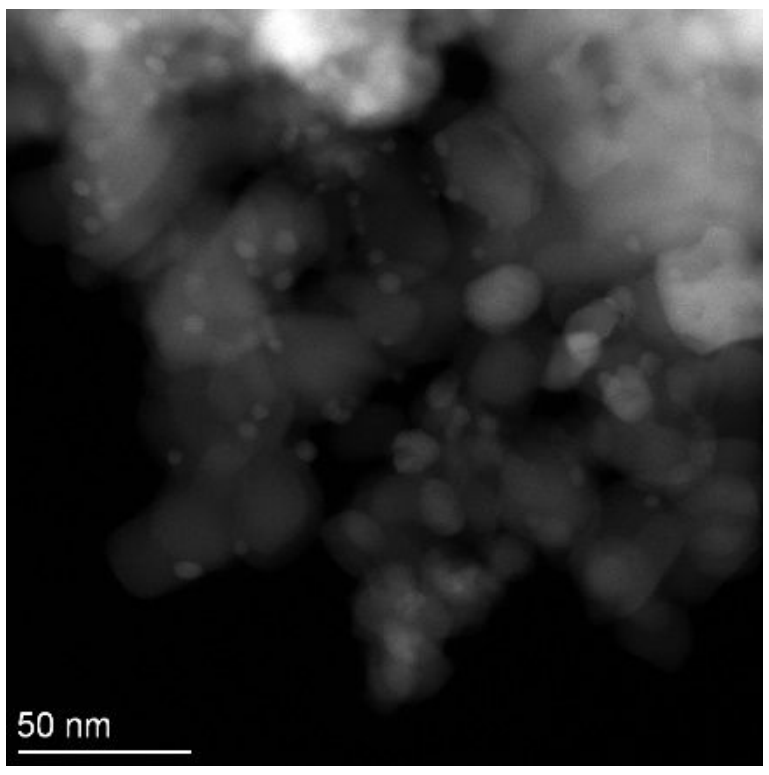

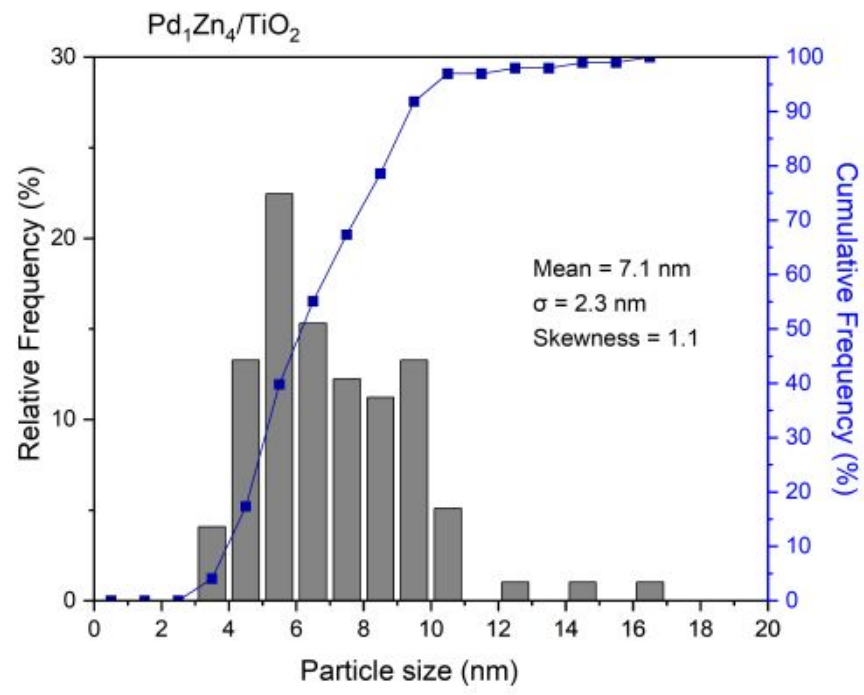

This work

5

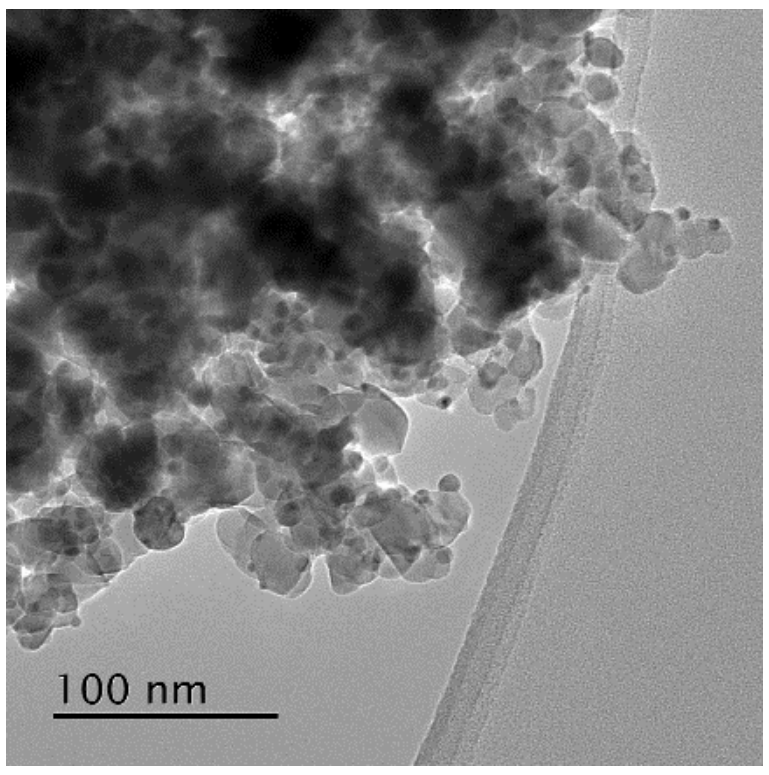

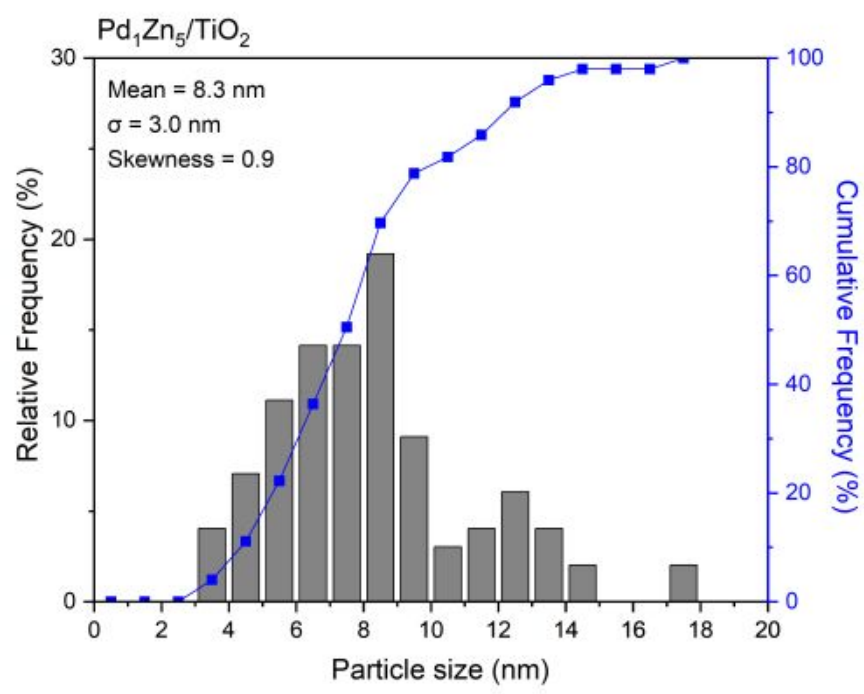

This work

10

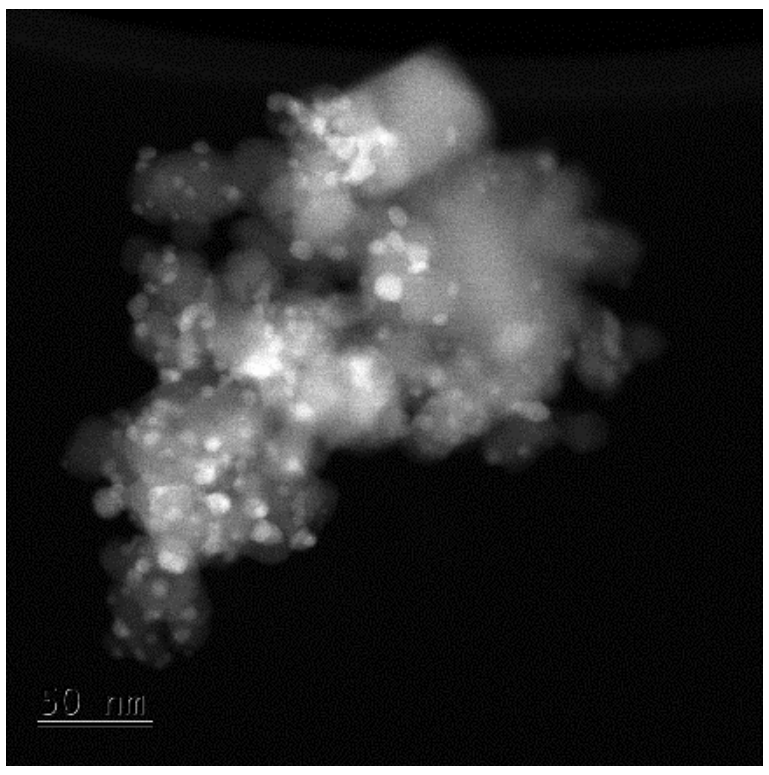

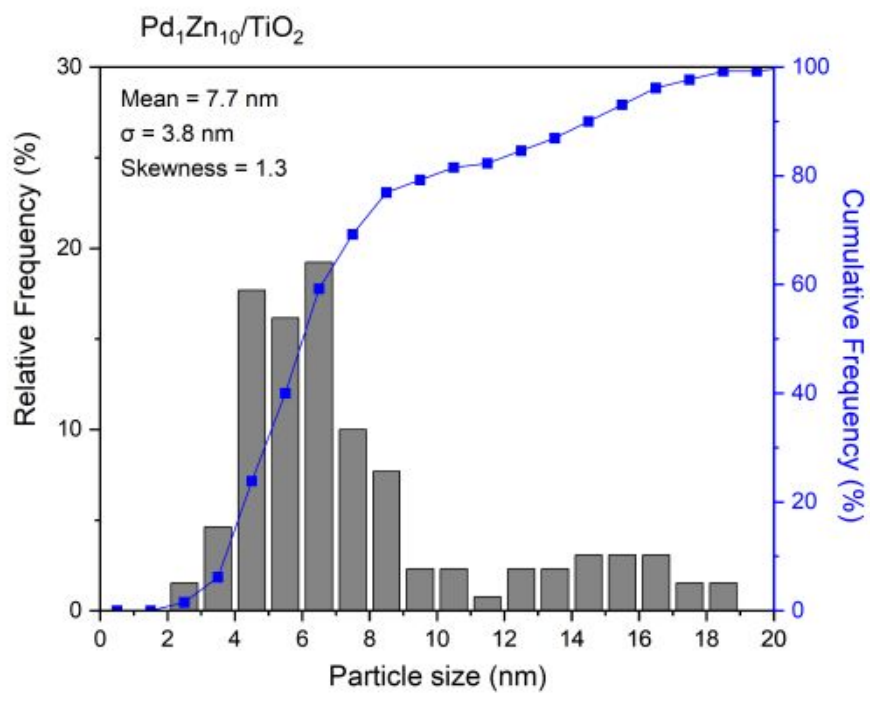

This work

15

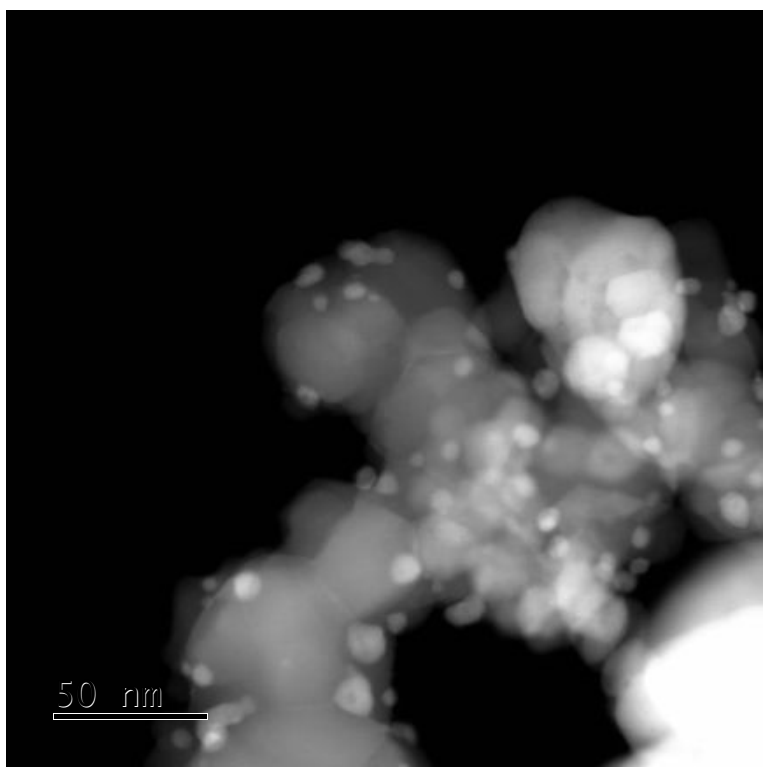

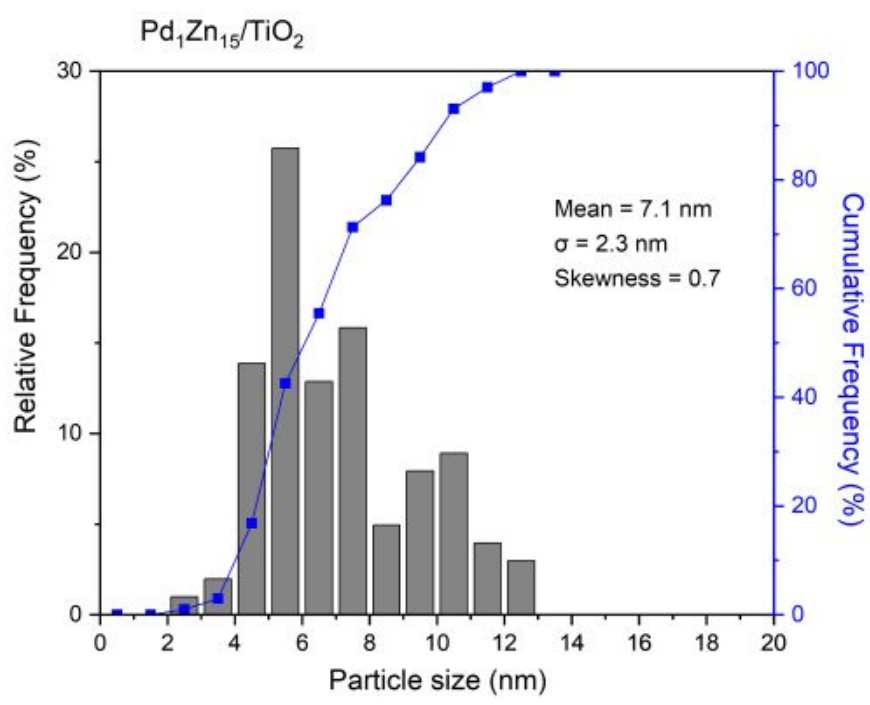

This work

20	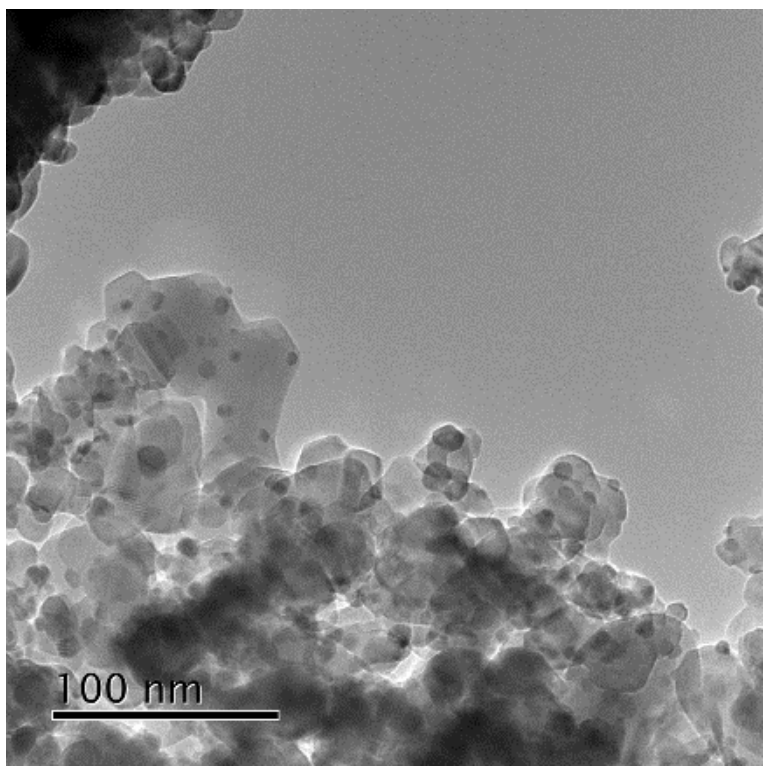	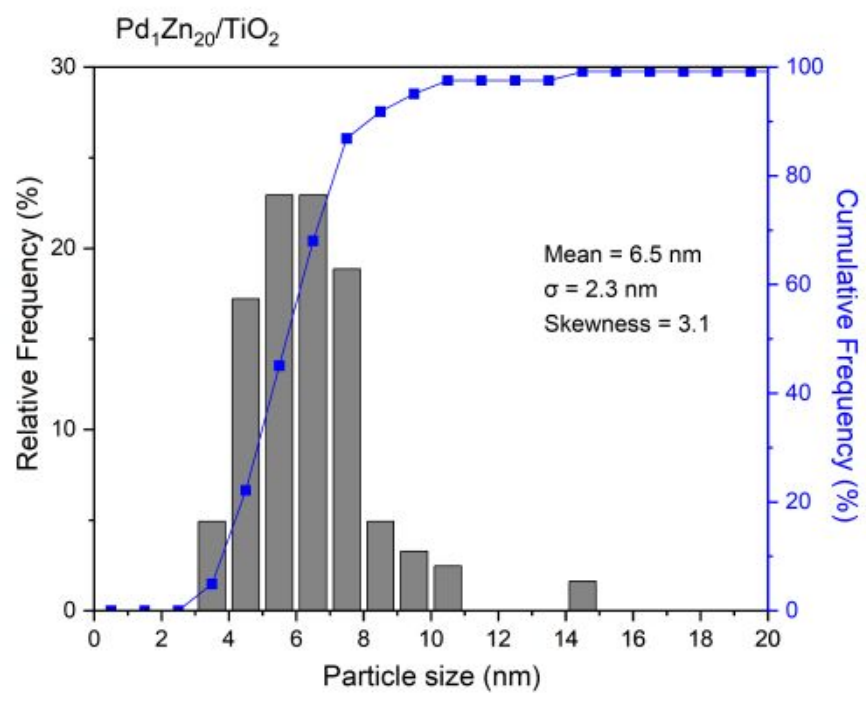	This work

S7 Computational Results

Hydrogen adsorption

The interaction of hydrogen with the Pd and PdZn surfaces was studied by calculating the adsorption strength of a hydrogen atom in symmetry unique adsorption sites shown in Figure 1 and 2. The symmetry unique adsorption sites and corresponding adsorption energies (E_{ads}) for atomic H on the FCC Pd(110) surface, which was previously highlighted as the catalytically most active low-index Pd facet for CO_2 hydrogenation,[14] are -0.06, -0.17, -0.29, -0.38 and -0.47 eV on atop, hollow, long-bridge, short-bridge and 'FCC' sites, respectively. Given the low affinity of Zn metal towards hydrogen, an extensive adsorption survey was not performed on the Zn surface; however, the highly endothermic E_{ads} of H on Zn(0001) was calculated as 0.72 eV, in agreement with a previously reported value of 0.76 eV.[20]

The adsorption of atomic hydrogen on the Pd(110) FCC site was calculated to be -0.47 eV, in good agreement with previous work by Dong *et al.* (-0.50 eV).[21] The E_{ads} calculated with the mBEEF exchange correlation functional compares well also to the result of -0.56 eV by Kowalec *et al.* when using the PBE exchange-correlation functional with a Tkatchenko-Scheffler van der Waals correction.[14]

Three metastable geometries of hydrogen atom adsorbed on β-PdZn(101) and six metastable geometries on β-PdZn(110) were found. On β-PdZn(101), the hydrogen atom is most stable in a Pd-Pd off-bridge position with a Zn atom in the sublayer (Table S.iii) with E_{ads} of -0.25 eV. On β-PdZn(110) the hydrogen atom adsorption is endothermic on all adsorption sites, least unstable on the Pd-Pd bridge site (Table S.vii) with E_{ads} of 0.05 eV.

In general, the preferred sites for hydrogen atom adsorption have high Pd-coordination and low Zn-coordination. Previously, the strong E_{ads} of H on Pd surfaces was highlighted as an inhibitor of CO_2 hydrogenation on Pd catalysts,[14] and therefore the considerably weaker adsorption observed on PdZn might help facilitate the reaction.

Table S5. E_{ads} of a hydrogen atom on β-PdZn(101) and (110) surfaces; blue, grey and white spheres represent Pd, Zn and H atoms, respectively; the black lines represent the simulation cell boundaries as viewed in the xy-plane.

**Structure**	**Surface**	**Visualization**	**Sites**	E_{ads} **/eV**
i	PdZn (101)	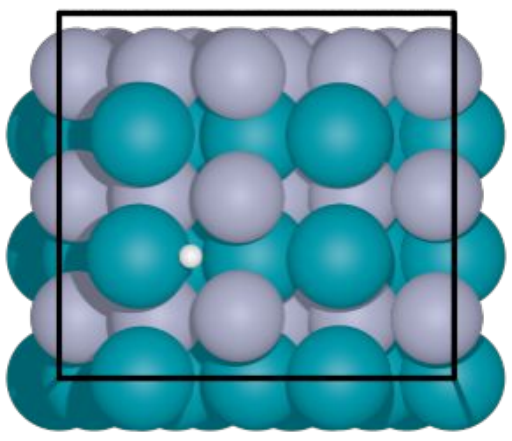	C	-0.01
ii	PdZn (101)	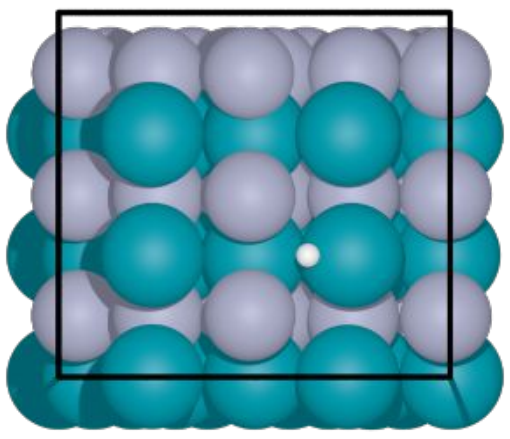	A	0.17
iii	PdZn (101)	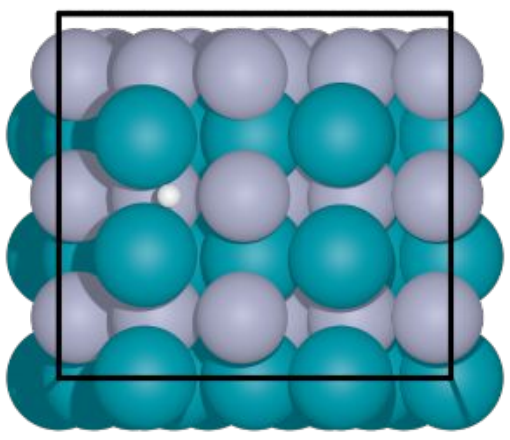	G	-0.25
iv	PdZn (110)	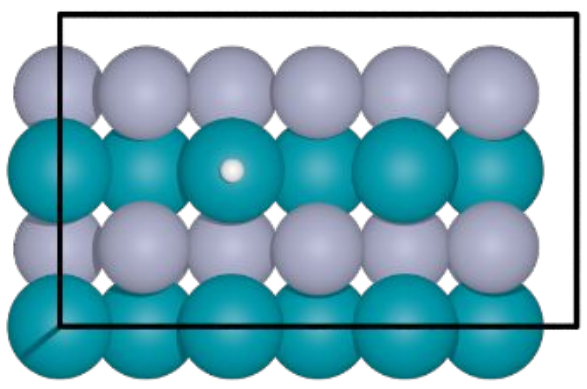	F	0.31
v	PdZn (110)	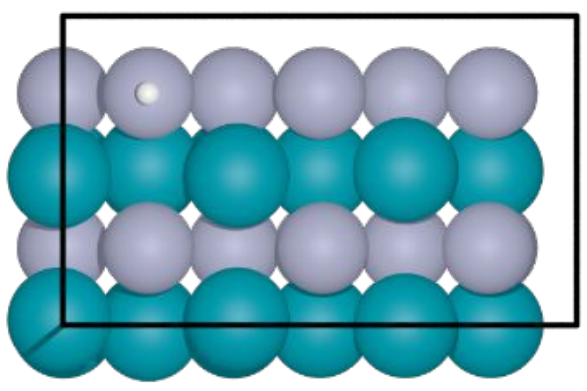	A	0.61

vi	PdZn (110)	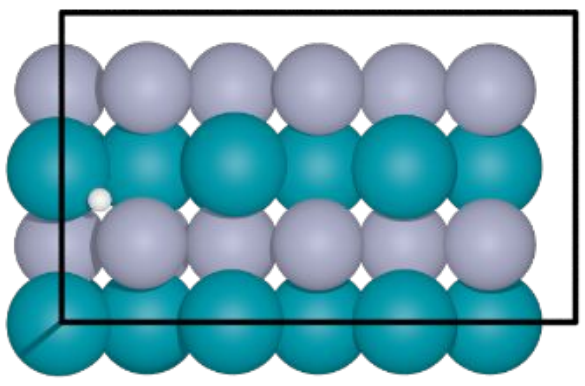	C	0.16
vii	PdZn (110)	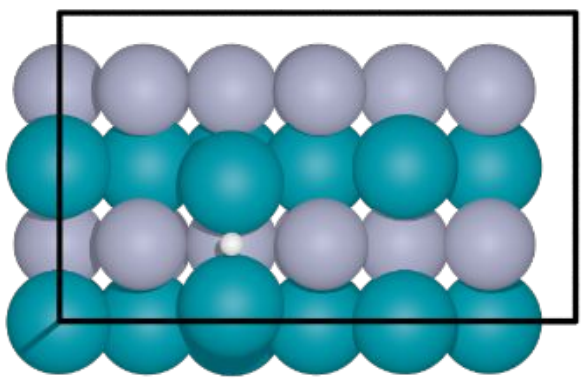	B	0.05
viii	PdZn (110)	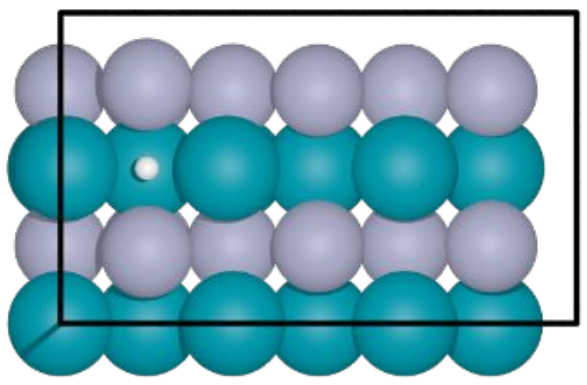	D	0.23
ix	PdZn (110)	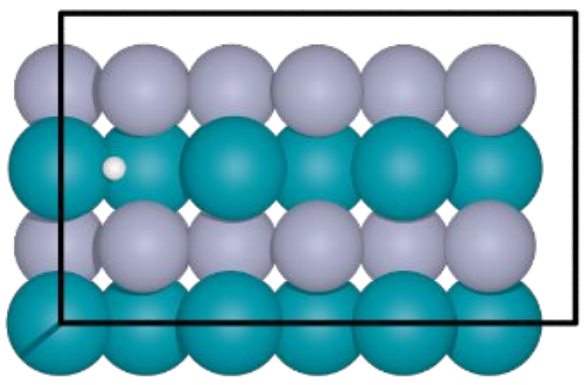	E	0.22

Table S6. E_{ads} of CO on FCC Pd (111), (100) and (110), and BCT PdZn(101) and (110) across the unique metastable adsorption sites; energy units are provided in eV.

surface	Site	E_{ads} /eV
**Pd (100)**	A – atop	-1.51
	C - hollow	-1.89
	**B - bridge**	**-1.99**
**Pd (110)**	E - hollow	-1.19
	B – atop	-1.50
	C - long-bridge	-1.74
	A - short-bridge	-1.99
	**D - ‘FCC’**	**-2.01**
**Pd (111)**	C - atop	-1.38
	B- HCP hollow	-1.98
	**A - FCC hollow**	**-2.02**
**PdZn (101)**	B - Pd atop	-1.08
	**F - Pd-Pd bridge**	**-1.10**
**PdZn (110)**	D – Pd_3Zn_2 hollow	-0.28
	B – Pd_2Zn_3 hollow (Pd-Pd distance shortened, akin to Pd-Pd bridge)	-0.88
	**F - Pd atop**	**-1.10**

S8 Quantification of Zn in alloy and in ZnO

The analytical data presented in this work suggests that at higher loading Zn is present both as part of a PdZn alloy with Zn:Pd ratio of 1 with excess Zn forming ZnO. In this section we quantify the moles of Zn required to form the alloy and the amount of excess required to form a monolayer coverage across the TiO_2 support. This will allow us to assess if ZnO is evenly distributed or present in clusters.

Table S1 shows that all catalysts used in this work contain close to the synthetic target of 5 wt.% Pd, so there are 4.8 x 10^{-4} moles of Pd per g of catalyst and the same number of moles of Zn are required to make the 1:1 PdZn alloy, which is supported by Figure 3a. At this composition EDX mapping shows Zn mostly co-located with Pd, indicative of PdZn alloy but little ZnO. Even so, a Pd metal reflection is still observed in XRD (Figure 2) and a corresponding signal is seen in XPS (Figure 9). Pd XANES also indicates that a mix of PdZn alloy and Pd metal are present (Figure 4).

The TiO_2 support has a surface area of 55 m^2 g^{-1} as obtained by N_2 adsorption (**Table S1**), From the Wurtzite surfaces studied here, ZnO has ~ 9 × 10^{19} atoms m^{-2} of Zn, a monolayer can be concluded to require 8.22 x 10^{-3} moles of Zn (Equation S5 and S6).

$$55 \times 9 \times 10^{19} = 4.95 \times 10^{21}\ atoms\ g^{-1}$$ Equation S5

$$\frac{4.95 \times 10^{20}}{6.022 \times 10^{23}} = 8.22 \times 10^{-3}\ mol_{Zn}\ g^{-1}$$ Equation S6

This means that a composition of Pd_1Zn_{18}/TiO_2 should be sufficient to form PdZn alloy and evenly distribute ZnO at a monolayer coverage.

When considering the formation of ZnO on TiO_2, there are three main growth modes that are reported for deposition of one material upon another.[22] In the equilibrium theory of heteroepitaxial growth, Frank-van der Merwe describes layer-by-layer growth whilst Volmer-Weber suggests separate island growth; and Stranski-Krastanov depicts a combination where initially a layer-by-layer growth is observed, often only one or two layers, followed by island growth. These growth modes are illustrated in Figure S11, and influenced by the interface energies and the lattice mismatch between the two substituent materials.[22]

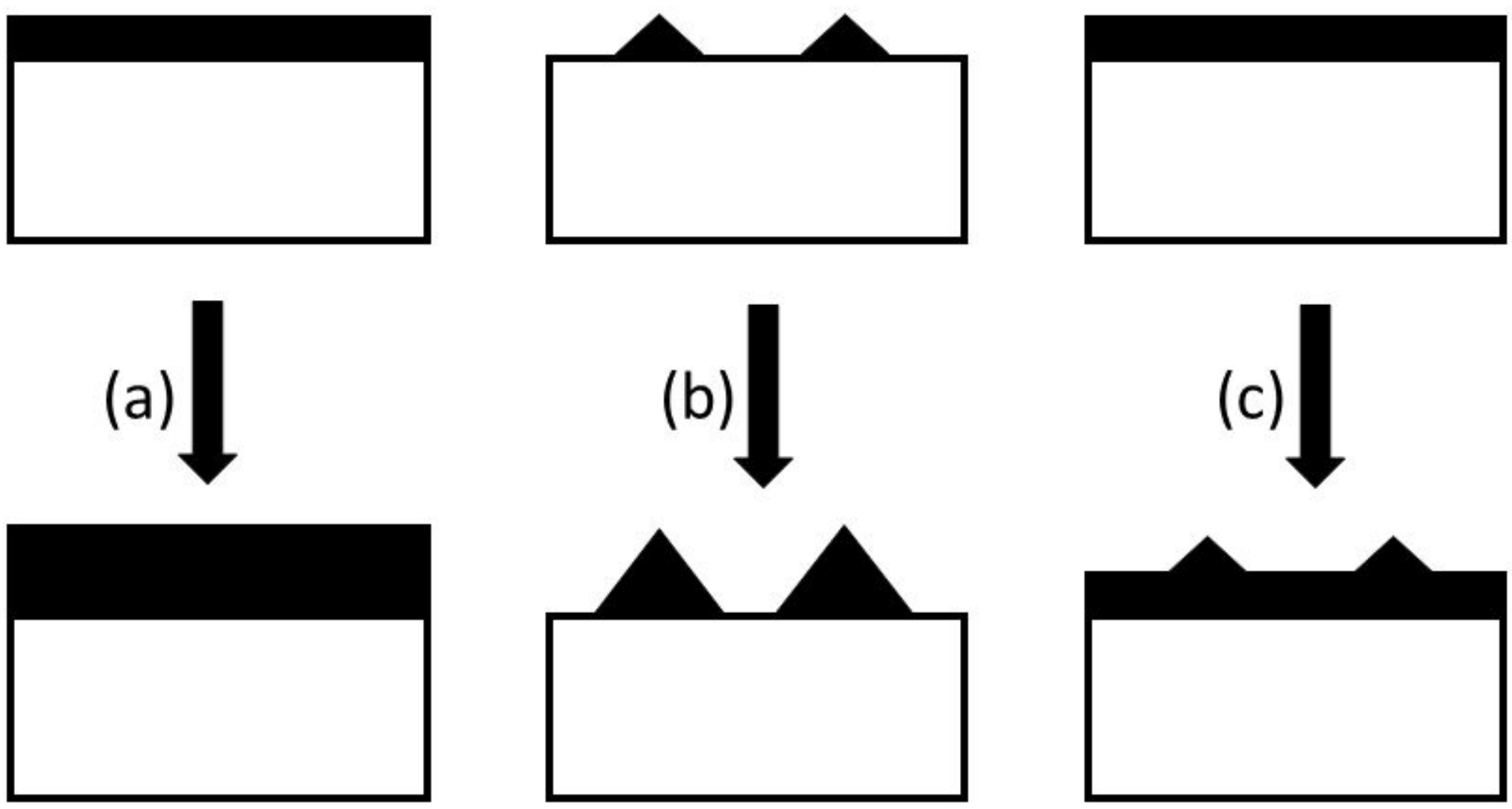

Figure S72. Schematic diagrams of deposition growth modes: (a) Frank-van der Merwe, (b) Volmer-Weber, (c) Stranski-Krastanov.

The growth of ZnO in our system follows closely to that of a Stranski-Krastanov (evidenced by STEM-EDX mapping of ZnO/TiO_2), as we see coverage of Zn on the surface of the TiO_2 (Figure 3) but also cluster-like formations. Additionally, from XRD we observe the ZnO reflections from ratios Zn:Pd > 3, evident of bulk-like ZnO.[23,24] At ratios of excess Zn, it is therefore likely that ZnO saturates the surface, and this correlates to the plateau in the catalysts reactivity, as discussed previously[25] (Figure 1).

References

(1) van Beek, W.; Safonova ,Olga V.; Wiker ,Geir; and Emerich, H. SNBL, a Dedicated Beamline for Combined in Situ X-Ray Diffraction, X-Ray Absorption and Raman Scattering Experiments. *Phase Transit.* **2011**, *84* (8), 726–732. https://doi.org/10.1080/01411594.2010.549944.
(2) Marshall, K. P.; Emerich, H.; McMonagle, C. J.; Fuller, C. A.; Dyadkin, V.; Chernyshov, D.; Beek, W. van. A New High Temperature, High Heating Rate, Low Axial Gradient Capillary Heater. *J. Synchrotron Radiat.* **2023**, *30* (1), 267–272. https://doi.org/10.1107/S1600577522009845.
(3) Ravel, B.; Newville, M. ATHENA, ARTEMIS, HEPHAESTUS: Data Analysis for X-Ray Absorption Spectroscopy Using IFEFFIT. *J. Synchrotron Radiat.* **2005**, *12* (4), 537–541. https://doi.org/10.1107/S0909049505012719.
(4) Blum, V.; Gehrke, R.; Hanke, F.; Havu, P.; Havu, V.; Ren, X.; Reuter, K.; Scheffler, M. *Ab Initio* Molecular Simulations with Numeric Atom-Centered Orbitals. *Comput. Phys. Commun.* **2009**, *180* (11), 2175–2196. https://doi.org/10.1016/j.cpc.2009.06.022.
(5) Hjorth Larsen, A.; Jørgen Mortensen, J.; Blomqvist, J.; Castelli, I. E.; Christensen, R.; Dułak, M.; Friis, J.; Groves, M. N.; Hammer, B.; Hargus, C.; Hermes, E. D.; Jennings, P. C.; Bjerre Jensen, P.; Kermode, J.; Kitchin, J. R.; Leonhard Kolsbjerg, E.; Kubal, J.; Kaasbjerg, K.; Lysgaard, S.; Bergmann Maronsson, J.; Maxson, T.; Olsen, T.; Pastewka, L.; Peterson, A.; Rostgaard, C.; Schiøtz, J.; Schütt, O.; Strange, M.; Thygesen, K. S.; Vegge, T.; Vilhelmsen, L.; Walter, M.; Zeng, Z.; Jacobsen, K. W. The Atomic Simulation Environment—a Python Library for Working with Atoms. *J. Phys. Condens. Matter* **2017**, *29* (27), 273002. https://doi.org/10.1088/1361-648X/aa680e.
(6) Kabalan, L.; Kowalec, I.; Catlow, C. R. A.; Logsdail, A. J. A Computational Study of the Properties of Low- and High-Index Pd, Cu and Zn Surfaces. *Phys. Chem. Chem. Phys.* **2021**, *23* (27), 14649–14661. https://doi.org/10.1039/D1CP01602D.
(7) Wellendorff, J.; Lundgaard, K. T.; Jacobsen, K. W.; Bligaard, T. mBEEF: An Accurate Semi-Local Bayesian Error Estimation Density Functional. *J. Chem. Phys.* **2014**, *140* (14), 144107. https://doi.org/10.1063/1.4870397.
(8) Lehtola, S.; Steigemann, C.; Oliveira, M. J. T.; Marques, M. A. L. Recent Developments in Libxc — A Comprehensive Library of Functionals for Density Functional Theory. *SoftwareX* **2018**, *7*, 1–5. https://doi.org/10.1016/j.softx.2017.11.002.
(9) Gómez-Jeria, J. S. An Empirical Way to Correct Some Drawbacks of Mulliken Population Analysis. *J. Chil. Chem. Soc.* **2009**, *54* (4), 482–485. https://doi.org/10.4067/S0717-97072009000400036.
(10) Peterson, E. J.; Halevi, B.; Kiefer, B.; Spilde, M. N.; Datye, A. K.; Peterson, J.; Daemen, L.; Llobet, A.; Nakotte, H. Aerosol Synthesis and Rietveld Analysis of Tetragonal (B1) PdZn. *J. Alloys Compd.* **2011**, *509* (5), 1463–1470. https://doi.org/10.1016/j.jallcom.2010.09.149.
(11) Nowicka, E.; Althahban, S. M.; Luo, Y.; Kriegel, R.; Shaw, G.; Morgan, D. J.; He, Q.; Watanabe, M.; Armbrüster, M.; Kiely, C. J.; Hutchings, G. J. Highly Selective PdZn/ZnO Catalysts for the Methanol Steam Reforming Reaction. *Catal. Sci. Technol.* **2018**, *8* (22), 5848–5857. https://doi.org/10.1039/C8CY01100A.
(12) Hansen, M. H.; Torres, J. A. G.; Jennings, P. C.; Wang, Z.; Boes, J. R.; Mamun, O. G.; Bligaard, T. An Atomistic Machine Learning Package for Surface Science and Catalysis. arXiv April 1, 2019. https://doi.org/10.48550/arXiv.1904.00904.
(13) Henkelman, G.; Uberuaga, B. P.; Jónsson, H. A Climbing Image Nudged Elastic Band Method for Finding Saddle Points and Minimum Energy Paths. *J. Chem. Phys.* **2000**, *113* (22), 9901–9904. https://doi.org/10.1063/1.1329672.
(14) Kowalec, I.; Kabalan, L.; Catlow, C. R. A.; Logsdail, A. J. A Computational Study of Direct CO_2 Hydrogenation to Methanol on Pd Surfaces. *Phys. Chem. Chem. Phys.* **2022**, *24* (16), 9360–9373. https://doi.org/10.1039/D2CP01019D.
(15) Grosvenor, A. P.; Kobe, B. A.; Biesinger, M. C.; McIntyre, N. S. Investigation of Multiplet Splitting of Fe 2p XPS Spectra and Bonding in Iron Compounds. *Surf. Interface Anal.* **2004**, *36* (12), 1564–1574. https://doi.org/10.1002/sia.1984.

(16) Zanatta, A. R.; Chambouleyron, I. Local Electronegativity and Chemical Shift in Si and Ge Based Molecules and Alloys. *Solid State Commun.* **1995**, *95* (4), 207–210. https://doi.org/10.1016/0038-1098(95)80086-7.
(17) Rodriguez, J. A. Interactions in Bimetallic Bonding: Electronic and Chemical Properties of PdZn Surfaces. *J. Phys. Chem.* **1994**, *98* (22), 5758–5764. https://doi.org/10.1021/j100073a031.
(18) Rightor, E. G.; Hitchcock, A. P.; Ade, H.; Leapman, R. D.; Urquhart, S. G.; Smith, A. P.; Mitchell, G.; Fischer, D.; Shin, H. J.; Warwick, T. Spectromicroscopy of Poly(Ethylene Terephthalate): Comparison of Spectra and Radiation Damage Rates in X-Ray Absorption and Electron Energy Loss. *J. Phys. Chem. B* **1997**, *101* (11), 1950–1960. https://doi.org/10.1021/jp9622748.
(19) Lawes, N.; Aggett, K. J.; Smith, L. R.; Slater, T. J. A.; Dearg, M.; Morgan, D. J.; Dummer, N. F.; Taylor, S. H.; Hutchings, G. J.; Bowker, M. Zn Loading Effects on the Selectivity of PdZn Catalysts for CO_2 Hydrogenation to Methanol. *Catal. Lett.* **2024**, *154* (4), 1603–1610. https://doi.org/10.1007/s10562-023-04437-5.
(20) Cremaschi, P.; Tantardini, G. F.; Muilu, J.; Pakkanen, T. A. Adsorption of Hydrogen on Zinc—Calculation of Potential Energy Surface and Dynamics. *Vacuum* **1990**, *41* (1), 260–264. https://doi.org/10.1016/0042-207X(90)90329-W.
(21) Vitos, L.; Ruban, A. V.; Skriver, H. L.; Kollár, J. The Surface Energy of Metals. *Surf. Sci.* **1998**, *411* (1), 186–202. https://doi.org/10.1016/S0039-6028(98)00363-X.
(22) Shchukin, V. A.; Ledentsov, N. N.; Bimberg, D. Spontaneous Formation of Nanostructures on Crystal Surfaces. *Phys. E Low-Dimens. Syst. Nanostructures* **2001**, *9* (1), 140–148. https://doi.org/10.1016/S1386-9477(00)00188-0.
(23) Farag, H. K.; Hanafi, Z. M.; Dawy, M.; Abd El Aziz, E. M. Characterization of ZnO Nanopowders Synthesized by the Direct Precipitation Method. *Can. J. Pure Appl. Sci.* **2010**, *4* (3), 1303–1309.
(24) Marcì, G.; Augugliaro, V.; López-Muñoz, M. J.; Martín, C.; Palmisano, L.; Rives, V.; Schiavello, M.; Tilley, R. J. D.; Venezia, A. M. Preparation Characterization and Photocatalytic Activity of Polycrystalline ZnO/TiO2 Systems. 2. Surface, Bulk Characterization, and 4-Nitrophenol Photodegradation in Liquid−Solid Regime. *J. Phys. Chem. B* **2001**, *105* (5), 1033–1040. https://doi.org/10.1021/jp003173j.
(25) Bowker, M.; Lawes, N.; Gow, I.; Hayward, J.; Esquius, J. R.; Richards, N.; Smith, L. R.; Slater, T. J. A.; Davies, T. E.; Dummer, N. F.; Kabalan, L.; Logsdail, A.; Catlow, R. C.; Taylor, S.; Hutchings, G. J. The Critical Role of β-PdZn Alloy in Pd/ZnO Catalysts for the Hydrogenation of Carbon Dioxide to Methanol. *ACS Catal.* **2022**, *12* (9), 5371–5379. https://doi.org/10.1021/acscatal.2c00552.
